# MBGC2: Boosting compression via efficient encoding of approximate matches in genome collections

**DOI:** 10.1093/gigascience/giag008

**Published:** 2026-01-21

**Authors:** Tomasz M Kowalski

**Affiliations:** Institute of Applied Computer Science, Lodz University of Technology, ul. Stefanowskiego 18, 90-537 Lodz, Poland

**Keywords:** software, data compression, multiple genome compression, FASTA

## Abstract

**Background:**

FASTA is the primary format for representing DNA, RNA, and protein sequences. While progress has been made, specialized FASTA collection compressors still struggle with practical limitations and inconsistent performance across different datasets, hindering effective storage and transfer of large genomic datasets.

**Results:**

We present an enhanced version of the Multiple Bacteria Genome Compressor (MBGC), a high-throughput, in-memory algorithm for compressing genome collections. It relies on information about maximum exact matches in the compressed set to identify possibly long approximate matches. It encodes them even when they partially overlap, boosting the compression ratio by an average of 14% across bacterial datasets, while the reengineered multithreaded decoding speeds up decompression compared to its predecessor by around 40%. The compression ratio improvement is even more pronounced on other collections, reaching 18% for *Homo sapiens* and up to 55% for *Saccharomyces paradoxus*. MBGC2 performs consistently across diverse datasets and introduces practical features to ease data management, such as archive appending, repacking, fast content listing, and flexible decompression options. Benchmark tests covering nucleotide-based bacterial, viral, and human genome collections show that MBGC2 combines compression efficiency and processing speed. The tool supports working with single genomes or amino acid collections but does not guarantee such high performance in these cases.

**Conclusions:**

MBGC2 addresses critical limitations in genome collection compression by delivering reliable performance, improved compression ratios, and enhanced usability features. The consistent efficiency across diverse genomic datasets makes it a versatile tool for managing the growing volume of genomic data in research and clinical settings. The balance between compression ratio and speed positions MBGC2 as a practical solution for the storage and transfer of large genomic collections.

Key Points

- High compression performance across diverse genomic datasets from bacterial collections to human genomes
- Enhanced usability with flexible decompression options and archive, appending, repacking, and fast content listing capabilities
- Compression ratio improved by 14% over MBGC1 on bacteria via carefully engineered approximate match encoding
- Multithreaded decompression delivered $\sim$40% speed improvement over MBGC1 while maintaining memory efficiency

## Introduction

The amount of genomic data is increasing at an exponential rate [1]. While tools for the bioinformatics domain are being rapidly developed, repositories still seem to be dominated by gzip format and tar archives for representing sets of files. Such an approach usually fails to exceed the 4-fold compression for the DNA stream [2, 3]. Efficient and reliable compression tools could reduce storage requirements and speed up the transfer, analysis, and processing of large collections of data.

Genomic information is a challenging subject for the development of compression methods. DNA sequences tend to be highly redundant but contain many single-nucleotide variations. They also contain various structural rearrangements (e.g., inversions, translocations, fusions, and inverted repeats) [2, 4, 5]. Many other phenomena that occur can be mentioned, such as copy number variation and clusters of structural variants that are formed by cut-and-paste and copy-and-paste mechanisms [5, 6]. In addition, various factors (e.g., exogenous DNA contamination and environmental causes) add additional heterogeneity or artifacts to sequencing reads, affecting sequence quality and downstream analyses. Effective modeling and compression require techniques that are capable of handling such dynamic and imperfect genomic data [7, 8].

Modern genomic data compressors usually target specific data formats. Most often, these are FASTA files oriented to store complete or partial sequences from 1 or many individuals or strains, and FASTQ files, which have become the standard for storing output from high-throughput sequencing instruments. Compressors can be broadly divided into 2 groups depending on whether they use an external reference sequence for compression and decompression. Both approaches have already been implemented in Biocompress, the first specialized genome compressor [9]. So-called reference-based (vertical) methods leverage the similarities in a compressed nucleotide stream to a selected reference, encoding only the differences to achieve high compression. Examples include tools targeting FASTQ datasets, such as LW-FQZip [10] and RBFQC [11], and tools for FASTA, such as DNAzip [12], GenomeZip [13], IDoComp [14], NRGC [15], RSS [16], HRCM [17], memRGC [18], LMSRGC [19], and many more [20]. Reference-free (horizontal) methods completely remove the requirement for an external reference sequence by exploiting intrasequence redundancies (e.g., palindromes and statistical properties) and across sequences in the dataset. These tools commonly employ statistical or dictionary-based models, bit encoding, context modeling, run-length encoding, and Huffman coding. Notable examples are XM [21], MFCompress [22], Deliminate [23], DSRC2 [24], and Leon [25]. A recent trend in reference-free compression is the integration of machine learning models, such as neural networks, to further improve compression ratios. GeCo2 [26], GeCo3 [27], Jarvis3 [28], and HMG [29] exemplify this direction. Most reference-free tools are designed for standalone nucleotide sequences or assembled genomes in FASTA format (of the above, the exception is DSRC2 for FASTQ files). However, several tools are also capable of compressing sequencing data with quality streams in FASTQ, for example, BEETL [30,31], FQZComp [32], SPRING [33], NAF [34], and Jarvis3 [28]. Some compressors, such as Mstcom [35] and PgRC [36, 37], focus on compressing only the DNA sequence stream from FASTQ files, offering high efficiency for applications where quality scores are not required. It is worth noting that some implementations leverage hardware acceleration, such as CUDA-based solutions, to further speed up the compression of FASTQ data [38, 39].

Beyond compression, efficient indexing and search in genomic collections are essential for practical applications. Compressed indexes such as MuGI [40], JST [41], PgSA [42], BIGSI [43], and Block Trees [44] have expanded the capabilities for fast pattern queries, scalable indexing, and efficient analysis across large-scale genomic databases. The development of such tools is closely linked to the ideas behind advanced compressors, as both fields benefit from exploiting redundancy and structure in genomic data. Other solutions, like copMEM [45,46] and bfMEM [47], provide robust and scalable maximal exact match (MEM) finding, which is crucial for comparative genomics and pan-genomic analyses. These methods also contribute to the implementation of efficient compression [18, 36]. A major recent development is the design of robust strategies for large-scale microbial genome searches. The approach by Břinda et al. [48] shows that taking advantage of phylogenetic relationships to reorder and compress data can enable efficient and robust search across millions of microbial genomes. Their pipeline allows BLAST-like queries against all sequenced bacteria using ordinary computer hardware, providing solutions that were previously unfeasible (e.g., for aligning genes, plasmids, or entire sequencing experiments). The AllTheBacteria initiative [49] is pursuing the above strategy. It employs phylogenetic compression in collecting and curating over 2.4 M publicly assembled bacterial genomes into a searchable and accessible resource. Further, beyond storage and search, compression-based features have found direct application in downstream analysis. Studies have shown that compression-derived metrics, such as normalized compression or normalized compression distance, can be utilized for taxonomic classification and metagenomic analysis [50, 51]. Quantifying the similarity of sequences without explicit alignment provides an alternative approach for clustering, classification, and detection of evolutionary relationships among complex microbial communities. In summary, the synergy between indexing and analytics and compression underlie many scalable and effective strategies for managing, querying, and interpreting genomic data at population and metagenomic scales [52–54].

In this work, we focus on tools developed specifically for compressing genomic collections in FASTA format. When compression addresses multiple genomes, a compressor can integrate and advance ideas from both reference-based and reference-free approaches. Early solutions for compressing collections of genomes adapted the LZ77 algorithm to leverage redundancy across multiple genomes. To this end, RLZ [55] relied on a single selected reference sequence. Subsequent tools either explored strategies to expand the set of reference sequences (e.g., GDC and its successor [56, 57]) or used second-order compression on already reference-compressed files (e.g., FRESCO [58]). Nevertheless, these tools assumed that genome collections are given as sets of complete chromosomes and processed the data chromosome by chromosome. In contrast, contemporary *de novo* assemblies typically yield sets of contigs (so-called multi-FASTA format) of varying lengths, with unknown chromosomal origins for each contig. This shift increases the complexity of the compression problem.

While numerous tools, such as FASTA or general-purpose compressors, can be utilized for compressing collections of assembled nucleotide sequences, they suffer from performance variability across different datasets. Their individual design and purpose make each solution suffer from certain drawbacks or limitations that impinge on their usability and versatility (cf. Table 1). Good results in the compression of multi-FASTA collections can be achieved by relatively simple specialized tools. For instance, NAF [34] strips end-of-line (EOL) symbols before packing 2 nucleotides into a single byte and performs zstd compression on the backend. By enabling long-distance matching in zstd, NAF can boost the ratio in the case of highly repetitive data  [59]. The most recent advance in genomic collections compression is AGC [60]. Its method involves identifying so-called splitters (i.e., unique *k*-mers evenly distributed in the reference), which divide sequences into segments. Then, corresponding segments are encoded using the LZSS algorithm [61] and the zstd compressor. AGC is exceptional because it offers high compression performance along with advanced features such as fast access to any contig and archive updates.

**Table 1:** Comparison of FASTA compressor features (the ✓ symbol indicates a supported function, — otherwise)

		Geno-				MBGC	General-purpose tools
	NAF	zip	GDC 2	HRCM	AGC	1 / 2	(7z, zstd, bsc, pigz)
Handles collection of genomes natively^a^	–	–	✓	✓	✓	✓ / ✓	– (except 7z)
Compresses without explicit reference	✓	✓	–	–	–	✓ / ✓	✓
Decompresses without reference	✓	✓	✓	–	✓	✓ / ✓	✓
Preserves sequence line length^b^	✓	✓	✓	✓	–	– / ✓	✓
Preserves base letter case	✓	✓	✓	✓	–	✓ / ✓	✓
Preserves original path structure	–	–	✓	–	–	✓ / ✓	– (except 7z)
Handles single multi-FASTA file	✓	✓	–	–	✓	✓ / ✓	✓
Decompresses selected FASTA files	–	–	✓	–	✓	– / ✓	– (except 7z)
Random access to individual FASTA	–	–	–	–	✓	– / –	✓ (if not lumped$^{1}$)
Can append genomes to archive	–	–	–	–	✓	– / ✓	– (except 7z)
Compresses gzipped FASTA	–	✓	–	–	✓	✓ / ✓	–

^a^Some tools (i.e., NAF, BSC, pigz) require lumping genomes together to compress them into a single archive (e.g., using tar or mumu.pl script, available at https://github.com/KirillKryukov/mumu) or produce tar of multiple archives (Genozip).

^b^Some specialized genome compressors correctly preserve line length format in sequences only if it is consistent throughout a whole file (e.g., in case of NAF, GDC 2, MBGC2) or an individual sequence (e.g., HRCM).

Here, we present Multiple Bacteria Genome Compressor 2 (MBGC2), the successor to the Multiple Bacteria Genome Compressor (MBGC) tool [59], a novel compressor designed to provide consistent, high-performance compression of genome collections. It improves the reliability of its predecessor by fixing critical errors reported in [60] concerning decompression of huge bacterial collections (i.e., Blackwell dataset of $\sim$661 K assemblies [62]) and compression of collections with large FASTA files (e.g., containing whole *Homo sapiens* genomes). MBGC2 compresses most FASTA files losslessly, and in the case of obsolete or irregular files, it allows for lossy compression. Achieving better compression ratios is made possible by carefully designed and engineered efficient approximate matches encoding, which was inspired by previous works [18, 20, 39]. The techniques we have introduced include dynamic determination of approximate match boundaries using a *mismatch scoring routine* and a simple yet effective static *exclusive mismatch encoding matrix*. Finally, MBGC2 uses multithreaded decoding for significant speed gains and introduces practical features, such as archive listing, appending, repacking, and flexible decompression options.

## Methods Overview and Implementation

MBGC2 and its predecessor are built upon the concept of relative compression, leveraging redundancy within genome collections. Similar to approaches employed in previous works [56, 63], in MBGC, we utilized a reference buffer to capture shared patterns among the genomes. Specifically, MBGC uses the first genome in the collection as the initial reference. Subsequently, it identifies and stores both direct and reverse-complemented MEMs between the reference and each genome within the collection. The consideration of reverse complements is particularly suited to bacterial datasets. To further enhance compression efficiency, MBGC dynamically extends the buffer. Sequences that exhibit limited similarity to the existing reference, as determined by the exact matching criteria, are incorporated into the reference, effectively expanding the reference base and boosting the overall compression ratio. This dynamic approach, similar to techniques aimed at improving the quality of the reference [63], ensures that the buffer adequately captures the diversity within the genome collection and leads to more effective compression.

**Figure 1:**
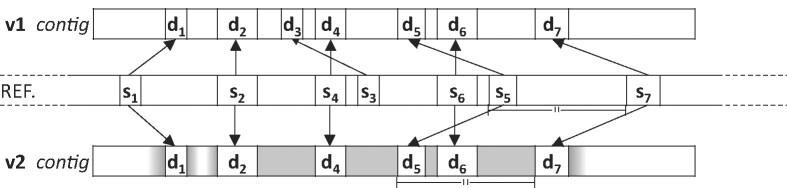
Toy example of contig encoding in MBGC1 and MBGC2. Regions encoded using the *encoding in gaps* technique are highlighted in solid gray (e.g., a region between second and fourth matches). A gray gradient in the spaces between matches (e.g., around $d_1$) indicates the span of *adjacent encoding*.

Unlike the original MBGC, which focused solely on MEMs, MBGC2 allows for a limited number of mismatches surrounding an identified MEM. This flexibility significantly improves the ability to capture similar regions even in the presence of minor variations between genomes. The contig coding example shown in Fig. 1 illustrates the evolution from MBGC1 to MBGC2. The subsequences $d_1$, $d_2$,..., $d_7$ in a contig represent MEMs in the reference denoted as $s_1$, $s_2$,..., $s_7$. Distances between $s_2$, $s_4$, and $s_6$ are equal to ones between $d_2$, $d_4$, and $d_6$, respectively. This applies also to matches with indexes 5 and 7. We denote all such groups of matches as *corresponding matches*, and nonempty regions between them are referred to as *gaps*. Note that, as in the example figure, groups of corresponding matches often overlap. In MBGC1, matches in the contig are replaced by a MATCH_MARK symbol (%), and the resulting sequence is appended to the literals stream. Information about the matches (i.e., lengths and beginning positions of $s_1$, $s_2$,..., $s_7$) are sent to separate streams. In MBGC version 2, there are 2 additional streams for more efficient encoding of matches and their adjacent surroundings. The final result is obtained by extending the original encoding schema with the following techniques, which allow individual matches or sets of corresponding matches to be treated more broadly as an approximate match.

**Figure 2:**
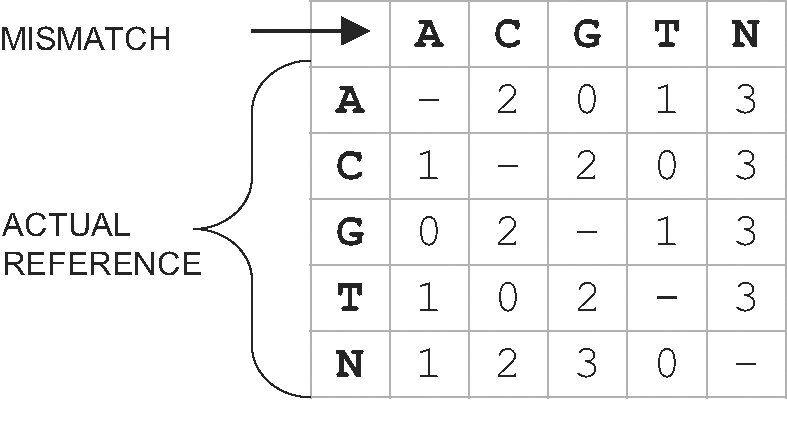
Mismatch encoding matrix.

**Figure 3:**
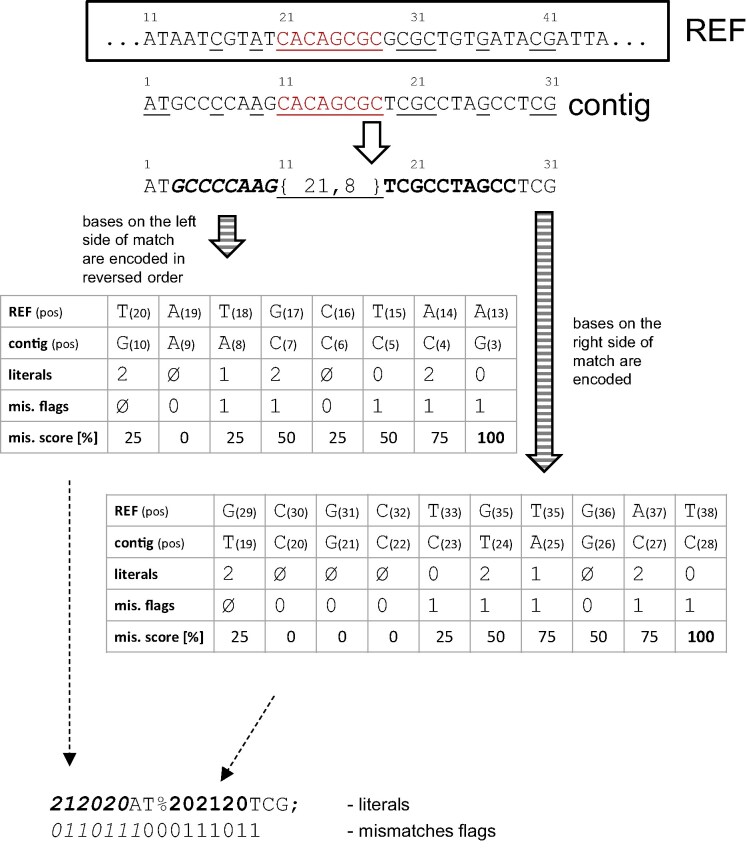
Toy example of encoding mismatches adjacent to a match. The maximum number of consecutive mismatches for the mismatch scoring routine is set to $x = 4$.

- Encoding in gaps: Bases within a gap are encoded relatively to the corresponding region in the reference. If corresponding bases are not equal (mismatch), a set flag is stored in the mismatches flags stream, and the mismatch is encoded in the literals stream. Mismatched bases are mapped by the *exclusive mismatch encoding matrix* (cf. Fig. 2) into values 0, 1, 2, and 3 and sent to the literals stream. This procedure aims to encode more likely point mutations as lower values. We experimented with a dynamic contextual approach (along the lines of the approach implemented in CURC [39]), but adjusting the matrix to the each genome did not yield a visible improvement in compression ratio and significantly complicated the compression and decompression process. Finally, we chose a static matrix favoring G$\leftrightarrow$A and C$\leftrightarrow$T transitions as well as higher overall AT content. Encoding using an exclusive mismatch matrix improves the compression ratio by up to 3.6 % (cf. Table 2).
- Adjacent encoding: If a direct neighborhood of match $d_i$ lies outside of a gap, a portion of the bases to the left and right of the match is encoded relatively to the neighborhood of $s_i$ in the reference. The encoding is stopped if relative frequency of mismatches exceeds a certain level. By introducing the *adjacent encoding* technique, we attempt to encode the sequence characters surrounding the MEM relatively to the corresponding characters in the reference. Bases adjacent to the MEM are encoded using the same 2 streams as in the case of encoding in gaps. Since the similarity between the sequence and the reference can deteriorate unpredictably, we decided to use a *mismatch scoring routine* to terminate the encoding of adjacent mismatches. The following parameters determine the behavior of this routine:
  -
$Y = 25\%$ is the initial score relative to the terminating threshold (i.e., 100%),
  -
$x = 10$ is the maximum number of consecutive mismatches causing termination of encoding, which translates into a 10% mismatch penalty, as well as an equal bonus (for each matching base).
  The scoring strategy with the above settings allows for longer approximate matches than the one proposed in memRGC [18], which was tailored to the genomes of *H. sapiens*. MEM expansion was terminated if the number of consecutive mismatching (resp. matching) bases was greater (resp. less) than 2 (resp. 3). The MBGC2 procedure for encoding the area adjacent to a MEM is explained in the toy example in Fig. 3 (note the nonstandard value of the *x* parameter). Eight bases of the contig, starting from the $11^{th}$ position, matched REF at position 21. The encoding mismatches routine starts from the left side of a match. As base G directly preceding the match is known to be a mismatch (otherwise, it would be incorporated into the MEM), there is no need for a mismatch flag, and the base is encoded relatively to base T ($20^{th}$ REF position) according to the exclusive mismatch encoding matrix into value 2 and pushed to the literals stream. At this point, the initial matching score (25%) is set. The following bases (in the reversed order) are processed sequentially. If a contig base matches a corresponding REF base (e.g., $9^{th}$ and $6^{th}$ contig positions), 0 flag is sent to the mismatches flags stream, and the mismatches score diminishes (25% bonus). In case of a mismatch (e.g., $8^{th}$ and $7^{th}$ contig positions), a mismatch flag is set, literals are extended with an exclusively encoded mismatch value, and the score is increased (25% penalty). Reaching a 100% mismatches score (at position 3 in a contig) terminates the encoding routine. Literals are appended with remaining bases AT (in the standard direction) and a match mark. Encoding of the right side of a match is done analogously but in the standard direction. Notice that the score cannot drop below 0 (contig position $20^{th}$ and following). Finally, the remaining bases TCG and a sequence mark are appended to the literals stream. Tests have shown that completely abandoning the improved coding of bases within gaps and adjacent to a match leads to a $\sim$10% average loss in ratio (cf. Table 2).
- Gap breaks filter: If matches adjacent to a short-enough match ($<$256 by default) are corresponding matches (e.g., $d_3$ between corresponding matches $d_2$ and $d_4$), then the match is ignored, and its bases are encoded with the encoding in gaps technique. Encoding bases in gaps in MBGC2 is now cheaper. We have observed that if a potential long gap is interrupted by a MEM that refers to a different reference region, it is sometimes beneficial to ignore it and rely on exclusive encoding of mismatches within the region of the match. Experimentally, we have determined that such MEMs should be discarded if they consist of fewer than $\sim$256 bases. This operation yields a $\sim$6% compression ratio improvement for large collections of bacteria (cf. Table 2).
- Gaps delta encoding: Not every beginning position of $s_i$ needs to be stored explicitly. Due to the similarity of genomic sequences, neighboring exact matches are likely to be mapped to corresponding locations in the reference. Specialized compressors exploit this to reduce the storage cost of their positions, for example, by employing delta encoding (e.g., GDC 2 [57] and HRCM [17]). In MBGC2, we took a different approach. Some of the beginning positions can be determined on the basis of the corresponding matches relationship (i.e., $s_4$, $s_6$, and $s_7$). For this purpose, in MBGC2, we added the *gaps delta* stream to store the number of *gaps* between the current match and its closest subsequent corresponding match, where a gap is a nonempty region between adjacent MEMs. This number is reduced by ignoring matches corresponding to other matches preceding the current match. MBGC2 detects corresponding matches within a contig if there are fewer than 64 other matches between them. If there is no corresponding match to the current match, a value of 0 is sent to the gaps delta stream. The last match cannot have a corresponding match following it and thus is ignored. On average, encoding matches offsets with the help of gaps delta stream yields a $\sim$5% overall ratio improvement. Let us consider gaps delta encoding using the example given in Fig. 1. Beginning positions of matches $s_1$, $s_2$, $s_4$, $s_5$, $s_6$, $s_7$ (assuming a match with index 3 was rejected by the gap breaks filter) are encoded now using 2 streams. The match offsets stream stores the positions of matches if they are not corresponding to the matches that precede them (i.e., $s_1$, $s_2$, and $s_5$), and the gap delta stream holds the values 0, 1, 2, 1, 0. The value for the $4^{th}$ match is 2 because its corresponding $6^{th}$ match is after the $5^{th}$ one. On the other hand, for a seemingly similar situation for the $5^{th}$ match, we obtain gap delta 1, because $s_6$ is not stored in the first stream. Such encoding allows for determining the beginning positions of the $s_4$, $s_6$, and $s_7$ matches during decoding.

**Table 2:** Boosting compression ratio with the implemented novel techniques. Ratio relative to MBGC2 max mode (-m3) in percentages (the ✓ symbol indicates an enabled technique, — otherwise).

	MBGC v2.0 -m3				v1.2.2
Options	-b	-X	-g0	-G0x0	-c3
Gaps delta encoding	✓	✓	–	✓	–
Gap breaks filter	–	✓	–	–	–
Encoding in gaps	✓	✓	–	–	–
Adjacent encoding	✓	✓	✓	–	–
Mismatch exclusion	✓	–	✓	–	–
*C. jejuni*	$-$ 7.9	$-$ 2.1	$-$ 1.1	$-$ 14.4	$-$ 10.3
*S. enterica*	$-$ 5.6	$-$ 1.1	$-$ 1.6	$-$ 7.7	$-$ 3.3
168,311 bacteria	$-$ 5.8	$-$ 1.7	$-$ 1.3	$-$ 11.3	$-$ 9.4
*C. jejuni* 1024	$-$ 3.6	$-$ 3.5	$-$ 7.6	$-$ 20.5	$-$ 25.0
*S. enterica* cluster	$-$ 0.3	$-$ 0.9	$-$ 2.9	$-$ 3.4	$-$ 4.0
*S. cerevisiae*	$-$ 1.1	$-$ 1.0	$-$ 8.6	$-$ 5.9	$-$ 13.7
*S. paradoxus*	$-$ 2.0	$-$ 2.3	$-$ 11.4	$-$ 19.9	$-$ 26.6
HGSVCu	$-$ 1.0	$-$ 1.7	$-$ 4.3	$-$ 4.1	$-$ 12.2
HPRC	$-$ 2.3	$-$ 2.1	$-$ 5.3	$-$ 5.9	$-$ 22.8

See supplementary material for more details on adjacent encoding, the matching scoring routine (cf. Supplementary Fig. S4), and considerations on encoding in gaps.

Finally, the resulting data streams representing approximate matches (offsets, lengths, gaps delta, mismatch flags), literals, headers, and filename data are compressed with the LZMA and PPMd algorithms. The combination of these new techniques implemented in MBGC2 yields a substantial reduction in the overall size of genome collections (cf. Table 2).

## Results

The benchmark was run on a Linux workstation equipped with a 14-core Intel Core i9-10940X 3.3 GHz CPU, 128 GB of DDR4-RAM (CL 16, 2666 MHz) and an SSD (ADATA 4 TB M.2 PCIe Legend 960). The test collection consists of several real genome datasets, used in prior works on FASTA collection compression. MBGC1 uses 8 threads, AGC 14 threads, NAF is single-threaded, and the rest of the tools use 28 threads. MBGC1 and AGC limit multithreading by their default settings. The detailed results of experiments on pathogens, *H. sapiens* genome collections, yeasts, and ribosomes are presented in Tables 3–6. The supplementary material provides more results (including Supplementary Table S8 with protein datasets and Supplementary Tables S11 and S12 presenting experiments with imposed limits on the number of threads), details on datasets, and the test methodology. Compression ratios are given as a ratio of input to output sizes. Compression (resp. decompression) times, presented in rows or columns as “ctime” (resp. “dtime”), are given in seconds. Peak memory usage, i.e., maximum resident set size of the process during its lifetime, “cmem” (resp. “dmem”) for compression (resp. decompression) and dataset sizes are specified in GB, where $\textrm {G} = 10^{9}$. The best three results are marked with a number in parentheses.

**Table 3:** Compression results—collections of pathogens

		zstd -3	Genozip	NAF -3	NAF -19	AGC	AGC -a	MBGC1	MBGC1	MBGC2	MBGC2
		–long=31	best	–long=31	–long=31	-a	adjusted^a^	default	max	default	max
	ratio	12.4	44.3	137.1	176.6	27.2	228.4	416.2	$^{(3)}$ 451.0	$^{(2)}$ 459.4	$^{(1)}$ 502.8
*C. jejuni*	ctime	$^{(3)}$ 233.3	2,152.7	440.1	2,332.8	3,117.5	804.8	$^{(2)}$ 76.4	322.8	$^{(1)}$ 61.0	257.6
55,627 genomes	dtime	116.5	1,553.6	241.5	241.0	113.3	261.1	$^{(3)}$ 49.0	70.0	$^{(1)}$ 32.2	$^{(2)}$ 34.9
98.38 GB	cmem	$^{(2)}$ 2.35	105.52	$^{(1)}$ 2.31	$^{(3)}$ 2.66	5.09	6.21	8.50	6.18	7.40	6.36
	dmem	$^{(2)}$ 2.15	63.86	2.70	$^{(3)}$ 2.69	3.95	$^{(1)}$ 0.78	5.65	5.06	4.20	4.93
*S. enterica*	ratio	27.5	65.9	1,981.5	2,175.6	100.0	3,325.2	7,516.8	$^{(3)}$ 7,671.2	$^{(2)}$ 7,804.9	$^{(1)}$ 7,990.6
Cluster	ctime	84.9	346.2	200.3	271.0	458.0	168.6	$^{(2)}$ 22.1	79.5	$^{(1)}$ 19.9	$^{(3)}$ 32.2
14,003 genomes	dtime	74.7	82.2	162.5	161.8	36.3	48.6	$^{(3)}$ 19.1	19.3	$^{(1)}$ 16.3	$^{(1)}$ 16.3
67.12 GB	cmem	$^{(2)}$ 2.36	99.15	$^{(1)}$ 2.31	2.66	4.07	3.90	6.33	$^{(3)}$ 2.46	2.59	2.48
	dmem	2.15	62.92	2.27	2.27	0.99	$^{(1)}$ 0.34	0.75	0.75	$^{(2)}$ 0.58	$^{(3)}$ 0.60
Bacteria	ratio	15.8	48.5	369.0	434.0	36.1	628.2	1,329.5	$^{(3)}$ 1,408.0	$^{(2)}$ 1,429.3	$^{(1)}$ 1,554.1
Mixed	ctime	1,009.2	28,698.0	2,101.6	6,511.0	9,540.0	3,895.0	$^{(2)}$ 280.3	1,117.5	$^{(1)}$ 225.3	$^{(3)}$ 660.6
168,311 genomes	dtime	683.5	26,590.0	1,431.9	1,428.5	545.1	1,337.5	$^{(3)}$ 274.7	307.1	$^{(1)}$ 240.3	$^{(2)}$ 261.5
587.26 GB	cmem	$^{(2)}$ 2.36	130.06	$^{(1)}$ 2.31	$^{(3)}$ 2.66	6.70	9.15	21.05	13.07	16.84	12.85
	dmem	$^{(2)}$ 2.15	65.07	$^{(3)}$ 4.51	4.53	16.86	$^{(1)}$ 1.61	9.11	9.50	8.09	8.78
	ratio	6.1	29.2	43.0	54.2	18.9	40.3	63.6	$^{(3)}$ 73.0	$^{(2)}$ 88.2	$^{(1)}$ 97.4
*C. jejuni*	ctime	$^{(3)}$ 5.0	61.5	8.7	121.6	52.5	17.7	$^{(1)}$ 3.8	10.7	$^{(2)}$ 4.4	10.0
1,024 genomes	dtime	$^{(2)}$ 1.7	6.2	4.5	4.5	2.4	2.7	2.6	3.3	$^{(1)}$ 1.5	$^{(3)}$ 2.1
1.78 GB	cmem	1.96	12.23	1.91	2.25	3.04	2.65	1.63	$^{(2)}$ 1.20	$^{(3)}$ 1.29	$^{(1)}$ 1.06
	dmem	1.79	5.99	1.76	1.76	$^{(2)}$ 0.37	$^{(1)}$ 0.31	1.26	1.18	$^{(3)}$ 0.90	1.00
*S. enterica*	ratio	20.6	60.4	900.4	1,024.3	62.3	1,314.5	2,174.6	$^{(3)}$ 2,197.3	$^{(2)}$ 2,229.0	$^{(1)}$ 2,252.7
Cluster part	ctime	6.4	47.0	15.7	25.2	44.6	12.2	$^{(2)}$ 2.5	6.0	$^{(1)}$ 1.7	$^{(3)}$ 2.8
1,024 genomes	dtime	3.1	4.7	11.8	11.8	2.7	3.4	$^{(3)}$ 1.3	1.4	$^{(1)}$ 1.0	$^{(2)}$ 1.1
4.87 GB	cmem	2.32	30.71	2.30	2.64	4.03	3.34	2.06	$^{(1)}$ 1.14	$^{(3)}$ 1.38	$^{(2)}$ 1.15
	dmem	2.15	16.22	2.16	2.16	$^{(2)}$ 0.35	$^{(1)}$ 0.28	0.62	0.62	$^{(3)}$ 0.48	0.51
Bacteria	ratio	14.1	47.6	177.1	214.6	11.2	172.0	301.0	$^{(3)}$ 351.5	$^{(2)}$ 378.1	$^{(1)}$ 456.8
Mixed	ctime	28.6	151.7	56.2	298.6	315.4	73.2	$^{(1)}$ 11.6	33.1	$^{(1)}$ 11.6	$^{(3)}$ 22.4
$4 \times {1024}$ gen.	dtime	10.2	59.9	36.2	36.2	20.5	14.4	$^{(3)}$ 7.3	8.2	$^{(1)}$ 4.9	$^{(2)}$ 6.1
14.94 GB	cmem	2.32	94.86	$^{(3)}$ 2.31	2.66	4.44	4.77	3.86	$^{(2)}$ 2.23	3.30	$^{(1)}$ 2.19
	dmem	2.15	49.72	2.24	2.24	$^{(2)}$ 1.77	$^{(1)}$ 0.51	2.53	2.25	$^{(3)}$ 1.90	2.03
	ratio	35.38	$^{(1)}$ 114.98	59.58	$^{(2)}$ 74.74	10.18	40.11	45.24	52.58	53.69	$^{(3)}$ 60.71
Influenza	ctime	$^{(3)}$ 6.39	224.29	$^{(2)}$ 6.23	121.01	913.09	957.96	$^{(1)}$ 5.78	12.60	6.48	11.78
817,587 seq.	dtime	$^{(1)}$ 0.69	2.43	$^{(3)}$ 1.11	$^{(2)}$ 1.09	5.90	858.58	5.54	5.16	3.83	5.02
1.43 GB	cmem	$^{(2)}$ 1.59	17.93	$^{(1)}$ 1.46	$^{(3)}$ 1.81	3.85	4.48	3.14	2.60	2.48	2.55
	dmem	$^{(3)}$ 1.43	2.97	$^{(3)}$ 1.43	$^{(3)}$ 1.43	2.77	4.84	1.65	2.31	$^{(2)}$ 1.08	$^{(1)}$ 0.96
	ratio	354.28	410.71	412.55	519.14	501.63	$^{(1)}$ 914.22	510.85	529.40	$^{(3)}$ 590.52	$^{(2)}$ 617.29
COVID-19	ctime	42.35	143.18	58.46	127.59	1,990.31	8,434.00	$^{(2)}$ 15.42	43.76	$^{(1)}$ 13.24	$^{(3)}$ 29.18
620,304 seq.	dtime	$^{(1)}$ 7.92	16.55	$^{(2)}$ 9.97	$^{(3)}$ 9.98	15.59	186.57	13.76	25.24	13.62	14.15
18.83 GB	cmem	$^{(1)}$ 2.31	105.87	$^{(1)}$ 2.31	$^{(3)}$ 2.65	22.24	22.15	21.34	21.46	20.04	19.80
	dmem	$^{(3)}$ 2.15	62.26	2.24	2.24	41.09	41.57	2.18	28.60	$^{(1)}$ 0.42	$^{(2)}$ 0.56

^a^AGC options adjusted for influenza and COVID-19 datasets are -s3000 -b10000 and -s1500 -b500 otherwise.

**Table 4:** Compression results—collections of *H. sapiens* genomes. NAF failed to compress the largest dataset within the 100,000- second limit (denoted with “—”).

	HGSVCu (36 genomes, 102.88 GB)					HPRC (95 genomes, 290.13 GB)				
	ratio	ctime	dtime	cmem	dmem	ratio	ctime	dtime	cmem	dmem
zstd -3 –long=31	3.3	$^{(1)}$ 180.7	154.9	$^{(2)}$ 2.35	$^{(1)}$ 2.15	3.4	$^{(2)}$ 514.9	466.5	$^{(2)}$ 2.35	$^{(1)}$ 2.15
zstd -19 –long=31	4.1	9,408.0	157.3	5.08	$^{(1)}$ 2.15	4.4	25,549.0	410.0	$^{(3)}$ 5.10	$^{(1)}$ 2.15
Genozip default	4.5	502.6	134.7	5.40	4.37	4.8	1,355.5	548.9	5.54	3.62
Genozip -b best	4.7	645.5	175.8	101.31	67.33	4.9	1,554.5	445.4	99.34	66.68
NAF -3 –long=31	4.2	740.0	337.2	$^{(1)}$ 2.30	$^{(1)}$ 2.15	4.1	2246.5	1,018.8	$^{(1)}$ 2.31	$^{(1)}$ 2.15
NAF -19 –long=31	5.2	49,906.0	333.2	$^{(3)}$ 2.64	$^{(1)}$ 2.15	–	–	–	–	–
AGC default	96.6	$^{(2)}$ 194.0	$^{(1)}$ 52.9	24.14	16.53	$^{(2)}$ 201.3	$^{(3)}$ 539.0	$^{(1)}$ 157.3	27.69	23.33
MBGC1 max	$^{(3)}$ 101.2	634.0	141.0	40.72	33.88	160.6	1611.3	398.6	42.49	40.58
MBGC2 default	$^{(2)}$ 101.4	$^{(3)}$ 205.4	$^{(2)}$ 82.1	46.41	37.00	$^{(3)}$ 180.5	$^{(1)}$ 248.2	$^{(2)}$ 162.7	51.67	43.28
MBGC2 max	$^{(1)}$ 115.2	631.4	$^{(3)}$ 106.2	38.48	36.94	$^{(1)}$ 208.1	1499.4	$^{(3)}$ 199.3	42.31	29.70

**Table 5:** Compression results—collections of yeast genomes

	*S. cerevisiae* (39 genomes, totaling 493.98 MB)					*S. paradoxus* (36 genomes, totaling 436.43 MB)				
	ratio	ctime	dtime	cmem	dmem	ratio	ctime	dtime	cmem	dmem
GDC 2	$^{(1)}$ 109.8	3.78	0.56	$^{(1)}$ 0.52	$^{(1)}$ 0.15	$^{(2)}$ 80.7	20.52	0.83	$^{(2)}$ 0.52	$^{(1)}$ 0.18
zstd -3 –long=31	4.2	$^{(2)}$ 0.90	0.56	$^{(2)}$ 0.56	0.50	3.8	$^{(1)}$ 0.71	$^{(3)}$ 0.52	$^{(1)}$ 0.50	0.44
zstd -19 –long=31	23.5	76.60	$^{(2)}$ 0.38	1.55	0.50	17.3	77.98	$^{(2)}$ 0.38	1.40	0.44
Genozip default	5.0	4.05	1.11	3.85	1.89	4.9	3.80	1.04	3.42	1.66
Genozip -b best	35.4	49.41	3.56	3.43	1.68	27.8	39.53	3.20	3.08	1.50
NAF -3 –long=31	67.0	2.61	1.04	0.64	$^{(3)}$ 0.49	43.2	2.33	0.92	$^{(3)}$ 0.58	$^{(3)}$ 0.43
NAF -19 –long=31	77.0	26.94	1.03	0.97	$^{(3)}$ 0.49	43.2	29.84	0.93	0.92	$^{(3)}$ 0.43
AGC default	70.3	1.50	$^{(1)}$ 0.32	$^{(3)}$ 0.59	$^{(2)}$ 0.33	43.4	$^{(3)}$ 1.63	$^{(1)}$ 0.28	0.80	$^{(2)}$ 0.32
MBGC1 default	86.6	$^{(3)}$ 1.21	0.75	1.94	0.81	43.9	1.64	1.06	1.94	0.75
MBGC1 max	91.0	3.07	0.92	1.49	0.88	61.4	3.00	1.09	1.42	0.76
MBGC2 default	$^{(3)}$ 101.5	$^{(1)}$ 0.88	$^{(3)}$ 0.47	1.84	0.53	$^{(3)}$ 76.4	$^{(2)}$ 1.36	0.54	1.82	0.51
MBGC2 max	$^{(2)}$ 105.5	2.69	0.75	1.40	0.75	$^{(1)}$ 83.6	2.82	0.81	1.42	0.74

**Table 6:** Compression results—collections of RNA

	SILVA 132 LSURef (610.3 MB)					SILVA 132 SSURef (3.28 GB)				
	ratio	ctime	dtime	cmem	dmem	ratio	ctime	dtime	cmem	dmem
zstd -3 –long=31	17.85	$^{(1)}$ 2.51	$^{(2)}$ 0.37	$^{(1)}$ 0.70	0.62	15.51	$^{(1)}$ 14.85	$^{(3)}$ 2.09	$^{(2)}$ 2.34	$^{(3)}$ 2.15
zstd -19 –long=31	37.20	28.68	$^{(1)}$ 0.34	2.05	0.62	32.44	167.61	$^{(1)}$ 1.72	4.75	$^{(3)}$ 2.15
Genozip default	37.31	18.91	1.07	9.49	1.43	33.97	111.00	$^{(2)}$ 1.97	14.21	2.67
Genozip -b best	$^{(1)}$ 51.98	207.56	1.93	8.09	1.34	$^{(1)}$ 42.74	316.30	3.59	39.36	6.72
NAF -3 –long=31	31.90	$^{(2)}$ 2.68	0.52	$^{(2)}$ 0.74	$^{(3)}$ 0.61	25.92	$^{(3)}$ 16.69	2.87	$^{(1)}$ 2.31	2.45
NAF -19 –long=31	41.49	43.78	$^{(3)}$ 0.50	$^{(3)}$ 1.08	$^{(3)}$ 0.61	34.97	421.16	2.72	$^{(3)}$ 2.66	2.45
AGC -a	12.71	40.42	2.20	1.66	1.47	11.56	7,839.00	16.34	8.92	8.59
AGC -a -s3000 -b10000	28.39	60.19	154.97	1.95	1.73	22.81	7,820.00	1957.78	9.70	7.88
MBGC1 default	37.91	$^{(3)}$ 2.90	2.24	1.37	0.67	31.45	$^{(2)}$ 15.73	14.34	5.51	2.49
MBGC1 max	$^{(3)}$ 45.54	5.43	2.24	1.23	0.99	34.06	44.17	14.60	5.41	4.93
MBGC2 default	44.49	2.95	1.61	1.21	$^{(1)}$ 0.50	$^{(3)}$ 36.99	20.79	10.23	5.41	$^{(2)}$ 2.00
MBGC2 max	$^{(2)}$ 50.82	5.33	2.28	1.22	$^{(2)}$ 0.52	$^{(2)}$ 37.62	43.47	14.11	5.42	$^{(1)}$ 1.83

The pathogen datasets are mostly the same as in our previous work [59], while the set of competitors now comprises zstd, Genozip, NAF, AGC, and the previous major public version of MBGC, v1.2. We limited the selection to reference-free compressors. The motivation behind presenting zstd as a representative of general-purpose compressors is to establish a benchmark for fast compression (in -3 mode) and decompression. NAF results are intended to show how high a ratio can be reached by combining the power of the aforementioned tool with relatively simple preprocessing [3]. Genozip [64] is a popular commercial compressor in the bioinformatics community. It targets various formats, but the authors do not describe the details of the reference-free FASTA file compression method. Unlike NAF, it offers support for multithreading and is actively developed. The last group of tested tools includes specialized ones—AGC, and MBGC—that are tailored for large multi-FASTA file collections and handle them natively. Except for AGC and MBGC1, which do not preserve the sequences’ line length, all tools performed lossless compression in all presented experiments. Information regarding preliminary experiments with other reference-free genome compressors (e.g., DELIMINATE, GeCo3, Leon, and MFCompress), as well as results for 7z, BSC, and a recent Jarvis3 [28], can be found in the supplementary material. Furthermore, supplementary material (subsection 4.1) presents results concerning boosting compression through leveraging phylogenetic relationships [48].

The results confirm the dominance of MBGC variants on bacterial collections (cf. Table 3). The next best tool in terms of compression ratio is usually AGC, which, however, requires adjusting its internal parameters. As compared to the default mode of MBGC2, it is about 2 times weaker in ratio and slower by up to 17.3 (resp. 8.1) times in compression (resp. decompression). Of the other tools, only NAF remains a viable choice. AGC and MBGC2 are the only tools that can take advantage of redundancy in *H. sapiens* genome collections (cf. Table 4) comprising large (more than 3 GB) FASTA files. Note that MBGC1’s default mode could not compress such large files due to a critical error. Both tools show high compression efficiency but slightly faster decompression, and around a 2-fold reduction in memory usage makes AGC the preferred option when using mid-range workstations or laptops with limited memory resources. In the experiment with 2 yeast genome collections (Table 5), we added results for the GDC tool. It could not be executed on other datasets as it assumes that the genomes are given as sets of chromosomes. MBGC2 and GDC 2 are quite fast and superior in the compression ratio. The former in the slowest max mode is faster in compression by a factor of 1.4–7.3, while the latter needs 25% less time to decompress for the *Saccharomyces cerevisiae* dataset. AGC offers a slightly worse ratio (as well as NAF) but has a noticeable lead in decompression speed. In the RNA compression ratio (cf. Table 6), MBGC2 is second only to Genozip. In the default mode, it loses up to 15%, with compression speeds only slightly weaker than the fast zstd and NAF variants. Unsatisfactory, however, is the inferior decompression performance, being up to 6 times worse than zstd.

One way to evaluate compressor performance is by looking at the worst-case scenarios and comparing its results with the best in the category. The strongest indicator of MBGC2 is the compression ratio. The archive size for a collection of *Influenza* virus sequences (cf. Table 3) is just over twice as large (2.14 to be exact) compared to the best competitor (namely, Genozip), which, however, required >30 times longer to compress. The next best tool that offers attractive compression levels (within an order of magnitude) is AGC. Specifically, with the adaptive mode (-a option designed for highly divergent species) enabled and settings suggested by the authors for the COVID-19 virus collection, in the worst-case scenario (i.e., also with *Influenza* dataset), the compression ratio is 2.9 times worse than Genozip. Yet, this is paid for with as much as 3 orders of magnitude slower decompression compared to zstd. Tuning the AGC for decompression speed degrades either the compression speed (by up to 3 orders of magnitude) or the compression ratio (sometimes by more than 1 order of magnitude). In terms of speed, MBGC2 performs worst with protein sequences (cf. Supplementary Table S8). It is slower in compression (resp. decompression) by a factor of 37 (resp. 20) than its fastest competitor (i.e., zstd). Excluding the protein collections, decompression is at most 7.6 times slower than the competition (for a small *Mitochondrion* dataset). Some general-purpose compressors can be tuned to provide high and stable speed (in both compression and decompression) across all datasets. In particular, zstd used with compression level 3 and long-distance matching enabled (–long=31 option) is at worst only 4.5 (resp. 4.7) times slower in compression (resp. decompression) than the best competitor (cf. 168,311 pathogens and *Salmonella enterica* cluster results in Table 3). However, the high speed comes at the cost of sometimes being more than 2 orders of magnitude less ratio-efficient than the most ratio-efficient tool.

In summary, the presented experiments (including additional ones in supplementary material conducted on various DNA datasets) show that MBGC2 is a tool offering a Pareto-optimal solution for the compression of genome collections, while being a strong practical compromise in terms of reliable speed and a high compression ratio.

## Conclusion

MBGC2 is a significantly enhanced version of the highly efficient MBGC tool for compressing collections of genomes stored in multiple FASTA files. Although it is capable of handling single or only a few genomes, its efficiency may be unsatisfactory. The implementation of MBGC2 is not tailored for collections stored in a single file, for which decompression can sometimes be several times slower than the competition. MBGC2 also supports amino acid sequences, but for protein datasets, it can be more than an order of magnitude slower than general-purpose compressors such as zstd in fast settings. With these exceptions, our tool achieves high compression ratios while maintaining reasonably high compression and decompression speeds. Unlike previous methods that may be sensitive to the composition or diversity of the input data, MBGC2 consistently delivers strong performance across a wide range of genome collections. It improves the compression ratio of its previous major version (v1.2) by 14% (across bacterial datasets), achieved through incorporating an encoding scheme that efficiently captures approximate matches between genomic sequences. Compared to recent FASTA file compression tools, such as GDC 2, HRCM, NAF, or AGC, which encode mismatches as literals, MBGC2 employs the exclusive mismatch encoding matrix that maps mismatches relative to the reference into 2-bit codes, prioritizing evolutionary transitions (G$\leftrightarrow$A, C$\leftrightarrow$T). This dedicated encoding is supplemented by a separate mismatches flags stream for efficient compression of sequence divergence from the reference. Furthermore, MBGC2 has been reengineered to use threads to decode different genomes, resulting in around 40% speedup. The release offers several new features such as more flexible decompression options, quick listing archive contents, repacking, and adding new files to already existing archives. It also fixes essential issues of MBGC1, such as those concerning the processing of extra-large files and collections. All of these enhancements make MBGC2 a highly efficient, reliable, and easy-to-use tool for the management, storage, and transfer of large collections of genomes stored in multiple FASTA files.

## Availability of Source Code and Requirements

- Project name: MBGC: Multiple Bacteria Genome Compressor
- Project homepage: https://github.com/kowallus/mbgc
- Operating system(s): Linux, MacOS, Windows
- Programming language: C++
- Other requirements: C++17 standard or higher, cmake 3.5 or higher
- License: e.g., GNU GPL v3.0
- biotools ID: mbgc
- RRID:SCR_021875

## Supplementary Material

giag008_Supplemental_File

giag008_Authors_Response_To_Reviewer_Comments_original_submission

giag008_Authors_Response_To_Reviewer_Comments_Revision_1

giag008_GIGA-D-25-00291_Original_Submission

giag008_GIGA-D-25-00291_Revision_1

giag008_GIGA-D-25-00291_Revision_2

giag008_Reviewer_1_Report_original_submission Divon Lan -- 9/7/2025

giag008_Reviewer_2_Report_original_submission Jorge Miguel Ferreira da Silva -- 9/10/2025

giag008_Reviewer_3_Report_original_submission Zexuan Zhu -- 9/18/2025

giag008_Reviewer_3_Report_revision_1 Zexuan Zhu -- 1/4/2026

## Data Availability

All supporting data are available in the *GigaScience* repository, GigaDB [65].

## Additional Files

**Supplementary Fig. S1**. Time of a dataset fraction decompression. Files are selected by evenly sampling all content; for example, when decompressing a 0.125 fraction of a dataset, files 1, 9, 17, 25, and so on are selected (according to the order of compression).

**Supplementary Fig. S2**. Time of appending an archive. The input archives for each dataset contain 1,024 files. The x-axes indicate a number of files added relatively to the input size (e.g., 0.125 dataset augmentation factor means adding 128 files to an archive).

**Supplementary Fig. S3**. Steps of the MBGC2 compression, on a high level, for a toy example. The basic parameters used for this example are seed length 6, $k = 8,\ m = 2,\ u = u_{RC} = 4\, s = 5$. (i) REF is created from the first genome, G1, of length 28 (REF is now the concatenation of G1 and rc(G1) and thus twice longer), and the hash table HT is populated with seeds taken from REF. (ii) Substrings from the first contig are sought in HT, and the found matches are left- and right-extended. Note how the skip margin idea is applied. The space between matches is sent to the literals stream, but it is partially encoded relatively to the surroundings of the matches (adjacent encoding), with additional information written into the mismatches flags stream (the details are explained in Supplementary Fig. S4). After the contig is processed, the decision is made if REF should be extended (affirmative here). (iii) REF is appropriately extended, and HT is updated; newer seeds overwrite older ones in case of a collision. The new seeds in HT are marked red and additionally with a bold typeface when overwriting. (iv) Processing another contig. Note how the skip margin idea allows for swallowing the previously found match (thus reducing the number of output matches). The decision of whether REF should be extended is negative this time. (v) The final stream of literals and matches, where matches are only marked with % symbol and contig separators are marked with ; symbol, plus supplemental mismatches flags stream. The prefix of this stream of length 28 is the G1 genome.

**Supplementary Fig. S4**. The encoding of mismatches adjacent to a match for a toy example. The parameters used for this example determine the behavior of the mismatch scoring routine: $x = 4,\ Y = 25\%$. (i) The case from Fig. 3(iv). Seventeen bases of the contig prefix match REF at position 9. The encoding mismatches routine is applied only to the right side of a match. As base G directly succeeding the match is known to be a mismatch (otherwise, it would be incorporated into the match), there is no need for a mismatch flag, and the base is encoded relatively to base C (26th REF position) according to the exclusive mismatches encoding matrix into value 2 and pushed to the literals stream. At this point, the initial matching score (25%) is set. The following bases are processed sequentially. Encoding of mismatches is terminated by reaching the end (or beginning) of the contig, a set flag is sent to the mismatches flags stream, and the contig separator is sent to the literals stream. (ii) The case from Fig. 3(ii). Another special case. Encoding of mismatches reaches an uninitialized part of REF (the current end). Four bases are encoded as mismatches in order to terminate the mismatches routine (by reaching the mismatches score limit). In the literals stream, contig bases are stored to avoid referring to an uninitialized REF region (during encoding and decoding).

**Supplementary Table S1**. Compression results—pathogen collections.

**Supplementary Table S2**. Compression results—collection of mixed pathogens.

**Supplementary Table S3**. Compression results—*S. enterica* cluster.

**Supplementary Table S4**. Compression results—661k pathogens collection.

**Supplementary Table S5**. Compression results—collections of human genomes.

**Supplementary Table S6**. Compression results—yeast collection.

**Supplementary Table S7**. Compression results for single-file inputs (single Multi-Multi-FASTA); RNA data.

**Supplementary Table S8**. Compression results for single-file inputs (single Multi-Multi-FASTA); protein data.

**Supplementary Table S9**. Compression results for single-file inputs (single Multi-Multi-FASTA); DNA data.

**Supplementary Table S10**. Boosting compression ratio with 6 new MBGC2 techniques of encoding matches and their adjacent surroundings relative to the reference sequence and technique for eliminating RC redundancy in the literals stream (available only in MBGC2 max mode -m3). Change in ratio relative to MBGC2 max mode in percentages.

**Supplementary Table S11**. Compression results—single-thread limit.

**Supplementary Table S12**. Compression results—6-thread limit.

## Abbreviations

AGC: Assembled Genomes Compressor; BIGSI: Bacterial and Viral Genomic Sequence Index; BLAST: Basic Local Alignment Search Tool; BSC: Block Sorting Compressor; CURC: CUDA-based Reference-Free Read Compressor; DSRC2: DNA Sequence Read Compressor 2; EOL: end-of-line (character); GCC: GNU Compiler Collection; GDC 2: Genome Differential Compressor 2; GeCo2: Genomic Compressor 2; GeCo3: Genomic Compressor 3; HGSVC: Human Genome Structural Variation Consortium; HPRC: Human Pangenome Reference Consortium; HRCM: Hybrid Referential Compression Method; JST: Journaled String Tree; LW-FQZip: Light-Weight Reference-Based Compression of FASTQ Data; LZ: Lempel-Ziv; LZMA: Lempel-Ziv-Markov chain algorithm; MBGC: Multiple Bacteria Genome Compressor; MEM: maximal exact match; MFCompress: Multi-FASTA Compressor; MuGI: Multi-Genome Index; NAF: Nucleotide Archival Format; NCBI: National Center for Biotechnology Information; NRGC: Novel Referential Genome Compression; PgRC: Pseudogenome-based Read Compressor; PgSA: Pseudogenome Suffix Array; PPMd: Prediction by Partial Matching (variant by Dmitry Shkarin); RBFQC: Referential-Based FASTQ Compressor; TAR: Tape Archive (file format); zstd: Zstandard.
