# Supplementary material for: MBGC2: Boosting compression via efficient encoding of approximate matches in genome collections
Source: Gigascience. 2026 Jan 21;15:giag008. doi: 10.1093/gigascience/giag008 (PMC12934354; doi:10.1093/gigascience/giag008)
Supplement: giag008_Supplemental_File [file giag008_supplemental_file.pdf]

Supplementary Material to: "MBGC2: Boosting compression via efficient encoding of approximate matches in genome collections"

Tomasz M. Kowalski

January 2026

1 Datasets

All the bacterial datasets used in the experiment are taken from the NCBI Pathogen Detection project (`https://www.ncbi.nlm.nih.gov/pathogens`) and include the following species:

- Campylobacter jejuni
  `https://www.ncbi.nlm.nih.gov/pathogens/isolates/#taxgroup_name%3A%22Campylobacter%20jejuni%22`
  (55,627 genomes, totalling 27,755 MB in gzip),
- E.coli and Shigella
  `https://www.ncbi.nlm.nih.gov/pathogens/isolates/#taxgroup_name%3A%22E.coli%20and%20Shigella%22`
  (22,523 genomes, totalling 33,708 MB in gzip),
- Listeria monocytogenes
  `https://www.ncbi.nlm.nih.gov/pathogens/isolates/#taxgroup_name%3A%22Listeria%20monocytogenes%22`
  (36,448 genomes, totalling 32,775 MB in gzip),
- Salmonella enterica:
  `https://www.ncbi.nlm.nih.gov/pathogens/isolates/#taxgroup_name%3A%22Salmonella%20enterica%22`
  (53,713 genomes, totalling 77,239 MB in gzip).

The full list of 168,311 bacterial genomes (URLs) used in our experiments is available at:

- `https://coach.iis.p.lodz.pl/mbgc2-datasets/lists/`

The experiment presented in Table 3 uses a subset of the largest pathogens cluster of Salmonella enterica genomes:

- `https://www.ncbi.nlm.nih.gov/pathogens/tree/#Salmonella/PDG000000002.3262/PDS000065758.1180`
  (14,003 genomes, totalling 67,118 MB in FASTA).

Additionally, we have conducted an experiment (cf. Table 4) with 661k pathogens collection:

- `https://ftp.ebi.ac.uk/pub/databases/ENA2018-bacteria-661k/`
  (661,405 genomes, totalling 2,640,685 MB in FASTA).

For experiments presented in Table 5 we use the following H. sapiens collections:

- Unphased haploid human genome assemblies generated by the Human Genome Structural Variation Consortium (HGSVC), phase 2
  `https://ftp.1000genomes.ebi.ac.uk/vol1/ftp/data_collections/HGSVC2/release/v1.0/assemblies/20200612_HHU_assembly-results_CLR_v12/assemblies/unphased/`
  (36 genomes, totalling 102,882 MB in FASTA),

- Phased haploid human genome assemblies generated by the Human Genome Structural Variation Consortium (HGSVC), phase 2
  `https://ftp.1000genomes.ebi.ac.uk/vol1/ftp/data_collections/HGSVC2/release/v1.0/assemblies/20200612_HHU_assembly-results_CLR_v12/assemblies/phased/`
  (36 genomes, totalling 104,385 MB in FASTA),
- Human genome haploid assemblies, including: CHM13 v2 assembly, and 94 haploid human assemblies released by the Human Pangenome Reference Consortium (HPRC) in 2021
  `https://github.com/human-pangenomics/HPP_Year1_Assemblies`
  `https://zenodo.org/records/7694116`
  (95 genomes, totalling 290,134 MB in FASTA).

We also use the following yeast collections (downloaded on Nov. 25, 2020):

- Saccharomyces cerevisiae
  `https://ftp.sanger.ac.uk/pub/users/dmc/yeast/latest/cere_assemblies.tgz`
  (39 genomes, totalling 494 MB in FASTA),
- Saccharomyces paradoxus
  `https://ftp.sanger.ac.uk/pub/users/dmc/yeast/latest/para_assemblies.tgz`
  (36 genomes, totalling 436 MB in FASTA).

Finally, in single FASTA file mode experiments (Tables 7–9) we use the following RNA, protein, and DNA datasets:

- SILVA 132 LSURef (downloaded on Oct. 7, 2021)
  `https://ftp.arb-silva.de/release_132/Exports/SILVA_132_LSURef_tax_silva.fasta.gz`
  (198,843 RNA sequences, totalling 610 MB in FASTA),
- SILVA 132 SSURef (downloaded on Oct. 7, 2021)
  `https://ftp.arb-silva.de/release_132/Exports/SILVA_132_SSURef_tax_silva.fasta.gz`
  (2,090,668 RNA sequences, totalling 3,282 MB in FASTA),
- H. sapiens GRCh38 peptides all (downloaded on Oct. 7, 2021)
  `https://ftp.ensembl.org/pub/release-96/fasta/homo_sapiens/pep/Homo_sapiens.GRCh38.pep.all.fa.gz`
  (109,914 protein sequences, totalling 73 MB in FASTA),
- PDB (downloaded on Oct. 7, 2021)
  `https://ftp.ncbi.nih.gov/blast/db/FASTA/pdbaa.gz`
  (132,163 protein sequences, totalling 98 MB in FASTA),
- UniProtKB Reviewed (Swiss-Prot) (downloaded on Oct. 7, 2021)
  `https://ftp.uniprot.org/pub/databases/uniprot/current_release/knowledgebase/complete/uniprot_sprot.fasta.gz`
  (562,254 protein sequences, totalling 280 MB in FASTA),
- Influenza (downloaded on Oct. 7, 2021)
  `https://www.ncbi.nlm.nih.gov/labs/virus/vssi/#/virus?SeqType_s=Genome&VirusLineage_ss=taxid:197911&VirusLineage_ss=taxid:197912&VirusLineage_ss=taxid:197913&VirusLineage_ss=taxid:1511083`
  (817,587 DNA sequences, totalling 1,429 MB in FASTA),
- Mitochondrion 1 (downloaded on Oct. 7, 2021)
  `https://ftp.ncbi.nlm.nih.gov/refseq/release/mitochondrion/mitochondrion.1.1.genomic.fna.gz`
  (7,819 DNA sequences, totalling 229 MB in FASTA),
- SARS-CoV-2 (downloaded on Sep. 10, 2022)
  `https://www.ncbi.nlm.nih.gov/datasets/coronavirus/genomes/`
  `https://zenodo.org/records/7694116/files/sars-cov-2_ncbi-620k.fa.xz?download=1`
  (619,750 DNA sequences, totalling 18,832 MB in FASTA).

For convenience, all collections used in our experiments are made available in various compressed formats: `https://coach.iis.p.lodz.pl/mbgc2-datasets`

2 Tested programs

The following programs, with the set parameters (e.g., for number of threads set to 6 in case of 7-zip), were used in our experiments, with their results presented either in the main paper or in Section 4 of the Supplementary Material.

GDC 2 (Genome Differential Compressor 2)
(version from 2020-Jun-24, https://github.com/refresh-bio/GDC2):

collection of FASTA files compression:
```
gdc2 c <archive-file> @<sequences-list-file>
```

collection of FASTA files decompression:
```
gdc2 d <archive-file>
```

BSC (Block Sorting Compressor)
(version 3.3.5 from 2025-Feb-07, https://github.com/IlyaGrebnov/libbsc/releases/tag/3.3.5):

single file (e.g., FASTA or tar) compression:
```
bsc e <in-file> -o <archive-file> -p -b2047
```

single file (e.g., FASTA or tar) decompression:
```
bsc d <archive-file> <out-file>
```

The number of worker threads used by BSC was OS-limited (up to 12).

7-Zip (x64)
(version 24.09 from 2024-Nov-29, https://www.7-zip.org/):

single file (e.g., FASTA or tar) compression:
```
7zz a -t7z -m0=lzma2 -mmt6 -mx=9 -md=4g <archive-file> <in-file>
```

single file (e.g., FASTA or tar) decompression:
```
7zz x -o<out-path> <archive-file>
```

zstd (64-bit)
(version 1.5.7 from 2025-Feb-19, https://github.com/facebook/zstd/releases/tag/v1.5.7):

single file (e.g., FASTA or tar) compression (level -3):
```
zstd -3 --long=31 -T0 <in-file> -o <archive-file>
```

single file (e.g., FASTA or tar) compression (level -19):
```
zstd -19 --long=31 -T0 <in-file> -o <archive-file>
```

single file (e.g., FASTA or tar) decompression:
```
zstd -d --long=31 -T0 <archive-file> -o <out-file>
```

Genozip
(version 15.0.68 from 2024-Oct-13, https://github.com/divonlan/genozip/releases/tag/genozip-15.0.68):

single FASTA file compression (default level):
```
genozip <in-file> --input fasta -o <archive-file>
```

single FASTA file compression (best level):
```
genozip <in-file> -b --input fasta -o <archive-file>
```

single FASTA file decompression:
```
genounzip <archive-file> -o <out-file>
```

JARVIS3
(version 3.7 from 2024-Oct-28, https://github.com/cobilab/jarvis3/releases/tag/v3.7):

single FASTA file compression (default level):
```
JARVIS3.sh --input <in-file> --fasta --level 7 --threads 28
```

single FASTA file decompression:
```
JARVIS.sh --decompress --input <archive-file> --fasta --threads 28
```

The name of the *<archive-file>* is *<in-file>* with added `tar` extension and the name of decompressed output is *<archive-file>* with added `out` extension (i.e., *<in-file>*`.tar.out`).

NAF (Nucleotide Archival Format)
(version 1.3.0 from 2021-May-17, https://github.com/KirillKryukov/naf):

single FASTA file compression (level -3):
```
ennaf <in-file> -3 --long 31 --text -o <archive-file>
```

single FASTA file compression (level -19):
```
ennaf <in-file> -19 --long 31 --text -o <archive-file>
```

single FASTA file decompression:
```
unnaf <archive-file> -o <out-file>
```

AGC (Assembled Genomes Compressor)
(version 3.2.1 from 2024-Nov-25, https://github.com/refresh-bio/agc/releases/tag/v3.2.1):

collection of FASTA files compression (default mode):
```
agc create -i <sequences-list-file> -o <archive-file> <ref-file>
```

collection of FASTA files compression (bacteria profile):
```
agc create -a -b 500 -s 1500 -i <sequences-list-file> -o <archive-file> <ref-file>
```

single FASTA file compression (adaptive mode):
```
agc create -a -o <archive-file> <ref-file> <in-file>
```

decompression:
```
agc getcol -l <sequence-line-length> -o <out-path> <archive-file>
```

MBGC1
(version 1.2.2 from 2022-Feb-22, https://github.com/kowallus/mbgc/releases/tag/v1.2.2):

collection of FASTA files compression (default level, -c 1):
```
mbgc -c 1 <sequences-list-file> <archive-file>
```

collection of FASTA files compression (max level, -c 3):
`mbgc -c 3 <sequences-list-file> <archive-file>`

single FASTA file compression (default level, -c 1):
`mbgc -c 1 -i <in-file> <archive-file>`

single FASTA file compression (max level, -c 3):
`mbgc -c 3 -i <in-file> <archive-file>`

decompression:
`mbgc -d <archive-file> <out-path>`

Note that the default MBGC compression (-c 1) can be run with a simplified syntax:
`mbgc <sequences-list-file> <archive-file>`

MBGC2
(version 2.1 from 2025-Dec-05, `https://github.com/kowallus/mbgc/releases/tag/v2.1`):

collection of FASTA files compression (default level, -c 1):
`mbgc c -m1 <sequences-list-file> <archive-file>`

collection of FASTA files compression (max level, -c 3):
`mbgc c -m3 <sequences-list-file> <archive-file>`

single FASTA file compression (default level, -c 1):
`mbgc c -m1 -i <in-file> <archive-file>`

single FASTA file compression (max level, -c 3):
`mbgc c -m3 -i <in-file> <archive-file>`

decompression:
`mbgc d <archive-file> <out-path>`

Note that the default MBGC compression (-m 1) can be run with a simplified syntax:
`mbgc c <sequences-list-file> <archive-file>`

2.1 FASTA files preprocessing

BSC, zstd, NAF, and Jarvis3 tools do not directly support compression of a FASTA files collection, so conversion to a single file is required. Genozip can compress multiple FASTA files directly into a standard tar file, but each file is compressed independently, without taking advantage of redundancy between genomes. For NAF, Jarvis3, and Genozip, to efficiently process multiple FASTA files, we combined them into a single Multi-Multi-FASTA file using mumu.pl (script and more information can be found at `https://github.com/KirillKryukov/mumu`).

Packing multiple FASTA files into a Multi-Multi-FASTA file:
`mumu.pl --stdin < <sequences-list-file> > <Multi-Multi-FASTA-file>`

Unpacking a Multi-Multi-FASTA file:
`mumu.pl --unpack --dir <out-path> < <Multi-Multi-FASTA-file>`

When experimenting with human collections, Genozip additionally required the use of the `--all` option to tag all sequences with file names, as it cannot handle compression of contigs with the same identifiers. For the remaining general-purpose compressors (i.e., BSC and zstd) we combined the input into a TAR archive. We have omitted the time of packing and unpacking in the results of our experiments.

To thoroughly test the general-purpose tools (zstd, BSC, and 7zip), we implemented an additional scenario so as not to impede their compression. In our data, possible long LZ-matches in DNA sequences are broken in "random" positions

with the EOL characters. To avoid this, we first remove these characters. The results obtained using this approach are presented separately in the bottom part of Table 2, Table 3, and Tables 5–9.

Table 1: Compression results – pathogen collections.

	zstd -3 –long=31	NAF -3 –long=31	NAF -19 –long=31	AGC -a	AGC -a -b500 -s1500	MBGC1 default	MBGC1 max	MBGC2 default	MBGC2 repo	MBGC2 max
					C. jejuni (55,627 genomes, totalling 98.38 GB)					
ratio	12.4	137.1	176.6	27.2	228.4	416.2	451.0	$^{(3)}$459.4	$^{(2)}$488.2	$^{(1)}$502.8
ctime	233.3	440.1	2332.8	3117.5	804.8	$^{(3)}$76.4	322.8	$^{(1)}$61.0	$^{(2)}$75.3	257.6
dtime	116.5	241.5	241.0	113.3	261.1	49.0	70.0	$^{(2)}$32.2	$^{(1)}$30.9	$^{(3)}$34.9
cmem	$^{(2)}$2.35	$^{(1)}$2.31	$^{(3)}$2.66	5.09	6.21	8.50	6.18	7.40	8.30	6.36
dmem	$^{(2)}$2.15	2.70	$^{(3)}$2.69	3.95	$^{(1)}$0.78	5.65	5.06	4.20	4.94	4.93
					E. coli (22,523 genomes, totalling 114.67 GB)					
ratio	15.9	460.9	458.5	11.8	632.7	1893.0	$^{(3)}$2057.0	1970.0	$^{(2)}$2123.5	$^{(1)}$2158.8
ctime	212.8	408.5	1218.2	2311.3	386.6	$^{(3)}$49.9	190.1	$^{(1)}$41.1	$^{(2)}$43.5	94.8
dtime	154.8	277.4	277.8	145.4	177.4	40.1	44.7	$^{(2)}$36.6	$^{(1)}$35.5	$^{(3)}$37.6
cmem	$^{(2)}$2.35	$^{(1)}$2.31	$^{(3)}$2.66	4.52	4.71	9.41	4.47	7.51	7.36	4.34
dmem	2.15	3.10	3.10	10.19	$^{(1)}$0.68	2.39	2.07	1.71	$^{(2)}$1.69	$^{(3)}$1.70
					L. monocyt. (36,448 genomes, totalling 112.0 GB)					
ratio	12.5	274.9	323.9	83.0	583.4	1092.0	$^{(3)}$1156.5	1108.3	$^{(2)}$1209.4	$^{(1)}$1258.1
ctime	224.0	406.3	1655.5	1448.3	740.9	$^{(3)}$53.2	224.6	$^{(1)}$47.6	$^{(2)}$53.0	148.2
dtime	139.9	271.6	271.6	92.1	231.6	43.8	53.0	$^{(2)}$38.7	$^{(1)}$33.8	$^{(3)}$39.5
cmem	$^{(2)}$2.35	$^{(1)}$2.31	$^{(3)}$2.66	4.50	5.27	8.33	4.87	7.73	7.58	5.08
dmem	$^{(3)}$2.15	2.39	2.39	$^{(2)}$1.77	$^{(1)}$0.59	3.35	2.93	2.72	2.99	2.94
					S. enterica (53,713 genomes, totalling 262.21 GB)					
ratio	20.1	1205.7	1312.0	88.8	2073.9	5794.9	5868.9	$^{(3)}$5962.3	$^{(2)}$5979.2	$^{(1)}$6071.8
ctime	370.9	835.8	1317.3	2457.1	832.4	$^{(3)}$90.3	331.4	$^{(1)}$79.5	$^{(2)}$80.8	164.8
dtime	300.4	632.5	633.8	160.1	488.4	110.8	115.5	$^{(2)}$93.8	$^{(1)}$91.8	$^{(3)}$97.4
cmem	$^{(2)}$2.35	$^{(1)}$2.31	$^{(3)}$2.66	4.33	4.37	10.74	4.11	5.42	5.74	3.98
dmem	2.15	2.79	2.79	3.47	$^{(1)}$0.65	1.95	1.77	$^{(2)}$1.38	1.48	$^{(3)}$1.47
					C. jejuni (1024 genomes, 1.78 GB)					
ratio	6.1	43.0	54.2	18.9	40.3	63.6	73.0	$^{(3)}$88.2	$^{(2)}$96.5	$^{(1)}$97.4
ctime	$^{(3)}$5.0	8.7	121.6	52.5	17.7	$^{(1)}$3.8	10.7	$^{(2)}$4.4	7.2	10.0
dtime	$^{(3)}$1.7	4.5	4.5	2.4	2.7	2.6	3.3	$^{(2)}$1.5	$^{(1)}$1.4	2.1
cmem	1.96	1.91	2.25	3.04	2.65	1.63	$^{(2)}$1.20	$^{(3)}$1.29	1.39	$^{(1)}$1.06
dmem	1.79	1.76	1.76	$^{(2)}$0.37	$^{(1)}$0.31	1.26	1.18	$^{(3)}$0.90	1.00	1.00
					E. coli (1024 genomes, 4.87 GB)					
ratio	43.5	495.2	530.0	5.4	485.0	1404.1	$^{(3)}$1451.6	1439.0	$^{(2)}$1466.5	$^{(1)}$1499.5
ctime	14.7	18.7	53.1	142.9	13.7	$^{(3)}$2.6	7.0	$^{(1)}$2.0	$^{(2)}$2.1	3.5
dtime	3.0	11.9	11.9	10.4	3.9	1.4	1.5	$^{(1)}$1.1	$^{(1)}$1.1	$^{(3)}$1.2
cmem	2.32	2.30	2.65	3.93	3.59	2.10	$^{(2)}$1.23	$^{(3)}$1.92	2.02	$^{(1)}$1.22
dmem	2.15	2.21	2.21	1.07	$^{(1)}$0.30	0.64	0.64	$^{(2)}$0.49	0.50	$^{(2)}$0.49
					L. monocyt. (1024 genomes, 3.09 GB)					
ratio	8.2	131.1	163.3	48.1	134.4	244.7	274.2	$^{(3)}$300.8	$^{(2)}$365.1	$^{(1)}$375.3
ctime	6.6	12.2	91.6	46.6	23.6	$^{(2)}$2.8	8.3	$^{(1)}$2.7	$^{(3)}$4.7	6.0
dtime	2.5	7.6	7.6	2.8	3.6	1.9	2.2	$^{(1)}$1.2	$^{(2)}$1.4	$^{(3)}$1.6
cmem	2.32	2.30	2.64	4.21	4.00	1.86	$^{(2)}$1.25	$^{(3)}$1.62	1.72	$^{(1)}$1.21
dmem	2.15	2.15	2.15	$^{(2)}$0.47	$^{(1)}$0.41	1.18	1.06	$^{(3)}$0.91	1.02	0.96
					S. enterica (1024 genomes, 5.20 GB)					
ratio	18.2	547.5	607.1	43.3	679.4	1315.4	1355.2	$^{(3)}$1385.0	$^{(2)}$1432.2	$^{(1)}$1457.3
ctime	7.3	18.0	33.2	63.4	10.0	$^{(3)}$2.7	7.0	$^{(1)}$2.1	$^{(2)}$2.2	4.1
dtime	3.3	12.6	12.6	3.1	3.5	1.6	1.7	$^{(2)}$1.3	$^{(1)}$1.2	$^{(3)}$1.4
cmem	2.33	2.30	2.65	4.08	1.93	2.22	$^{(2)}$1.23	$^{(3)}$1.83	1.99	$^{(1)}$1.22
dmem	2.15	2.17	2.17	$^{(2)}$0.42	$^{(1)}$0.32	0.71	0.71	$^{(3)}$0.56	0.57	0.57

The rows "ratio" show the ratio of the input to the output size. Compress / decompress times (as "ctime" / "dtime") are given in seconds, memory usages ("cmem" / "dmem") given in GB ($G = 10^9$). The best three results are marked with a number in parentheses.

Table 2: Compression results – collection of mixed pathogens.

	168,311 genomes (587.26 GB)					4 × 1024 genomes (14.94 GB)				
	ratio	ctime	dtime	cmem	dmem	ratio	ctime	dtime	cmem	dmem
BSC -p -b2047	28.9	4301.0	1717.1	128.84	128.84	28.9	142.0	44.1	74.71	74.73
7z -md4g	—	—	—	—	—	101.4	4676.0	13.1	122.58	15.09
zstd -3 –long=31	15.8	(5)1009.2	683.5	(2)2.36	(2)2.15	14.1	(5)28.6	10.2	(5)2.32	2.15
zstd -19 –long=31	63.2	35988.0	610.0	(5)4.85	(2)2.15	61.4	810.8	9.3	4.82	2.15
Genozip default	6.3	26716.0	27549.0	27.81	5.47	6.3	88.2	41.2	7.42	4.22
Genozip -b best	48.5	28698.0	26590.0	130.06	65.07	47.6	151.7	59.9	94.86	49.72
Jarvis3	—	—	—	—	—	92.6	756.0	222.7	(1)0.34	(1)0.34
NAF -3 –long=31	369.0	2101.6	1431.9	(1)2.31	(4)4.51	177.1	56.2	36.2	(4)2.31	2.24
NAF -19 –long=31	434.0	6511.0	1428.5	(3)2.66	(5)4.53	214.6	298.6	36.2	2.66	2.24
AGC default	5.6	73775.0	2145.2	(4)4.43	107.60	4.8	1691.8	49.0	4.33	4.77
AGC -a	36.1	9540.0	545.1	6.70	16.86	11.2	315.4	20.5	4.44	(3)1.77
AGC -a -b500 -s1500	628.2	3895.0	1337.5	9.15	(1)1.61	172.0	73.2	14.4	4.77	(2)0.51
MBGC1 default	(5)1329.5	(3)280.3	(4)274.7	21.05	9.11	(5)301.0	(1)11.6	(4)7.3	3.86	2.53
MBGC1 max	(4)1408.0	1117.5	(5)307.1	13.07	9.50	(4)351.5	33.1	(5)8.2	(3)2.23	2.25
MBGC2 default	(3)1429.3	(1)225.3	(1)240.3	16.84	8.09	(3)378.1	(1)11.6	(2)4.9	3.30	(4)1.90
MBGC2 repo	(2)1522.8	(2)245.5	(2)240.5	17.49	8.66	(2)450.7	(3)14.0	(1)4.7	3.43	2.06
MBGC2 max	(1)1554.1	(4)660.6	(3)261.5	12.85	8.78	(1)456.8	(4)22.4	(3)6.1	(2)2.19	(5)2.03
BSC* -p -b2047	105.1	3883.0	2484.6	128.85	129.01	85.0	125.9	40.5	73.80	73.82
7z* -md4g	—	—	—	—	—	**245.0**	3491.1	11.7	122.49	14.82
zstd* -3 –long=31	212.3	**852.5**	**511.1**	**2.36**	**2.15**	130.4	**29.4**	**9.1**	**2.32**	**2.15**
zstd* -19 –long=31	**376.7**	17263.0	548.2	4.75	**2.15**	226.1	345.0	**9.1**	4.71	**2.15**

The best five results are marked with a number in parentheses.
7z failed to compress the larger dataset in 100000 seconds limit (denoted with "—").
Compression of the larger dataset using Jarvis3 resulted in multiple error messages ("JARVIS3.sh: line 157: 33263 Killed JARVIS3 -l $LEVEL $file 2 .tmp_report_$file") and in discrepancies in decompressed FASTA files (denoted with "—").
* The results obtained with the general-purpose tools in the bottom four rows concern the scenario in which End-Of-Line (EOL) symbols were removed from sequences prior to the experiment. The best results in columns are marked in bold.

3 Test setup

All experiments, except for the compression of the 661k pathogen collection (due to excessive memory usage by the genome collection compressors) presented in Table 4, were run on a Linux (Debian) machine equipped with a 14-core Intel Core i9-10940X 3.3 GHz CPU, 128 GB of DDR4-RAM (CL 16, clocked at 2666 MHz) and an SSD (ADATA 4 TB M.2 PCIe Legend 960) with bandwidth limited to PCIe 3.0 x4 (Gen 3 expansion card with a four-lane configuration), running Linux (Debian 11) OS. MBGC2 is written in C++17 and was compiled with gcc 10.2.1 -O3 -DNDEBUG -mavx2. The disk cache was flushed between runs, to ensure raw reads of the input files from the disk. If not stated otherwise, BSC uses 12 threads, 7zip (up to) 6 threads, MBGC1 8 threads, AGC 14 threads, NAF is single-threaded, and the rest of the tools use 28 threads. Restrictions on the above general-purpose compressors have been imposed due to RAM limitations in order to maximize compression ratio. Specialized tools are limited by their design or lack of multithreading support in the case of NAF.

4 Additional tables and figures

While working on MBGC1, we tried to compare our software with many other genome compression tools: DELIMINATE, HRCM, memRGC, iDoComp, MtGC, GeCo3, Leon, and MFCompress. For various reasons, we found them to be inadequate or impractical for most scenarios of compressing large genome collections. The most common were numerous technical problems or limitations of the tools, such as issues with multi-FASTA format handling, errors during compression or decompression, very long running times, or significantly poorer compression or decompression performance. In some cases, additional data preprocessing was necessary, and even then, these tools proved uncompetitive. A detailed analysis is available in the supplement to the original MBGC article (Grabowski and Kowalski, 2022). In supplementary benchmarks, we have added Jarvis3 (Sausa *et al.*, 2023). This GeCo3-like tool represents state-of-the-art reference-free genomic compressors that achieve exceptional compression ratios through sophisticated probabilistic modeling and context mixing techniques. Jarvis3, in particular, pushes the boundaries of compression performance through its pioneering integration of specialized models, neural networks for context mixing, and probabilistic lookup tables in repeat models, enabling it to

Table 3: Compression results – S. enterica cluster.

	S. enterica cluster (14,003 genomes, 67.12 GB)					S. enterica cluster (1,024 genomes, 4.87 GB)				
	ratio	ctime	dtime	cmem	dmem	ratio	ctime	dtime	cmem	dmem
BSC -p -b2047	45.4	516.1	107.8	128.84	128.84	38.6	104.0	28.9	24.35	21.49
7z -md4g	339.9	22830.0	49.9	122.50	17.24	247.2	2830.3	5.4	47.07	4.89
zstd -3 –long=31	27.5	84.9	74.7	(3)2.36	2.15	20.6	6.4	3.1	2.32	2.15
zstd -19 –long=31	191.5	2729.3	50.4	4.76	2.15	148.1	238.7	3.0	4.72	2.15
Genozip default	6.3	307.0	112.7	7.52	4.27	6.1	20.7	4.6	6.03	4.10
Genozip -b best	65.9	346.2	82.2	99.15	62.92	60.4	47.0	4.7	30.71	16.22
Jarvis3	152.5	3546.2	1100.2	(1)0.34	(1)0.34	146.5	232.4	65.3	(1)0.34	(2)0.34
NAF -3 –long=31	1981.5	200.3	162.5	(2)2.31	2.27	900.4	15.7	11.8	2.30	2.16
NAF -19 –long=31	2175.6	271.0	161.8	2.66	2.27	1024.3	25.2	11.8	2.64	2.16
AGC default	95.6	465.6	36.5	3.96	1.01	60.5	43.0	2.7	4.02	(4)0.36
AGC -a	100.0	458.0	36.3	4.07	0.99	62.3	44.6	2.7	4.03	(3)0.35
AGC -a -b500 -s1500	3325.2	168.6	48.6	3.90	(1)0.34	1314.5	12.2	3.4	3.34	(1)0.28
MBGC1 default	(5)7516.8	(3)22.1	(3)19.1	6.33	0.75	(5)2174.6	(2)2.5	(4)1.3	2.06	0.62
MBGC1 max	(4)7671.2	(5)79.5	(5)19.3	(4)2.46	0.75	(4)2197.3	(5)6.0	(5)1.4	(2)1.14	0.62
MBGC2 default	(3)7804.9	(2)19.9	(2)16.3	2.59	(3)0.58	(3)2229.0	(1)1.7	(1)1.0	(4)1.38	(5)0.48
MBGC2 repo	(2)7868.1	(1)19.8	(1)16.0	2.59	(4)0.59	(2)2231.8	(1)1.7	(1)1.0	(5)1.72	0.49
MBGC2 max	(1)7990.6	(4)32.2	(2)16.3	(5)2.48	(5)0.60	(1)2252.7	(4)2.8	(3)1.1	(3)1.15	0.51
BSC* -p -b2047	212.6	459.3	123.1	128.84	128.84	172.3	95.5	25.8	24.05	21.48
7z* -md4g	958.6	16885.0	**47.2**	122.47	17.21	668.3	1959.6	4.8	45.99	4.82
zstd* -3 –long=31	1123.8	**80.9**	47.8	**2.35**	**2.15**	707.1	**7.1**	**3.0**	**2.31**	**2.15**
zstd* -19 –long=31	**1474.1**	779.1	47.6	4.64	**2.15**	**858.8**	62.8	**3.0**	4.62	**2.15**

The best five results are marked with a number in parentheses.
* The results obtained with the general-purpose tools in the bottom four rows concern the scenario in which End-Of-Line (EOL) symbols were removed from sequences prior to the experiment. The best results in columns are marked in bold.

Table 4: Compression results – 661k pathogens collection.

	AGC -a	AGC -a -b500 -s1500	MBGC1 default	MBGC1 -t 32	MBGC2 default	MBGC2 repo	MBGC2 max
	661,405 genomes (2640.69 GB)						
ratio	35.7	†95.0	82.4	55.1	(3)105.5	(2)117.9	(1)141.6
ctime	145482	†92770	(2)18427	(3)20154	(1)13609	23223	30262
dtime	9442	32585	*4034	*5875	(2)3394	(1)2919	(3)3681
cmem	(1)134.1	(2)†189.0	209.4	335.6	227.2	(3)197.9	330.1
dmem	(2)75.1	(1)35.0	*162.7	*256.2	153.9	(3)138.2	181.0

The best three results are marked with a number in parentheses. MBGC1 default uses 8 threads, and the rest of the tests were executed using 32 threads.
Test platform: 2 × Intel Xeon Platinum 8368 (38 cores) 2.4–3.4 GHz CPU, 1024 GB of DDR4-RAM (3200 MHz, CL 22) and an SSD (Intel 6.4 TB SSD NVMe U.2 P4610), running Windows Server 2019 Standard OS. MBGC1 and MBGC2 codes written in C++ and compiled with gcc 13.1.0 -O3. MBGC2 in version 2.0 was used for this experiment.
* In case of MBGC1 experiments, decompression was handled by MBGC2 due to errors during decompression of huge collections with MBGC1.
†AGC v3.2.1 failed to compress the dataset within 300000 seconds limit, therefore, AGC v3.0 was used for the compression instead.

achieve a record-breaking compression ratio on human genome[1].

In the tables, the compression ratios are given as the ratio of the input to the output size. Compress / decompress times (denoted as "ctime" / "dtime") are in seconds, and memory usages ("cmem" / "dmem") are in GB (G = 10^9).

Tables 1–4 show the results of compression and decompression for different bacterial datasets, ranging from homogeneous small clusters (cf. Salmonella enterica cluster of 1,024 genomes in Table 3) to large mixed collections of numerous pathogen species (cf. 661k pathogens collection in Table 4). All the general-purpose compressors tested are impractical, especially in terms of compression ratio, which is 4.5–24,6 times worse than the best (specialized) contender while being 2–3 orders of magnitude slower. The ratio could be improved by preprocessing EOL symbols, making zstd a viable choice. For bacterial collections, Genozip in the best mode offers a similar compression ratio to universal compressors, but in most cases, it is substantially slower in decompression. Compared to MBGC2, it is slower by at least an order of magnitude in compression. Jarvis3 tested in the default mode excels in memory consumption (below 0.4 GB and its compression performance is comparable to the stronger zstd mode, offering about twice a better compression ratio than Genozip. It is more than an order of magnitude slower than MBGC in compression and, due to its symmetrical nature, in decompression. The NAF tool, not being competitive with MBGC in terms of ratio and speed, has the advantage of low and constant memory consumption (as does zstd, which serves as its backend), never exceeding 3 GB. AGC default internal parameters are tuned for handling human genomes, which results in poor performance in the case of bacteria. Although setting recommended parameters (i.e., `-a -b500 -s1500`) yields a significant improvement, MBGC2 in its default mode beats AGC 1.7–3.1 times in ratio and 4.0–17.3 (resp. 1.8–8.1) times in speed of compression (resp. decompression). The exception is the experiment with the highly diverse 661k collection (cf. Table 4) where the compression ratio is only 1.1 times higher. The strength of AGC is its relatively low memory demand during decompression. All tools achieved the highest ratio compressing the Salmonella enterica cluster dataset (cf. Table 3). MBGC variants squeezed 14k genomes (totalling 67 GB) into archives of size smaller than 2 genomes, reaching nearly 8,000 times compression ratio for MBGC2 max mode.

Compared to MBGC1, for bacterial datasets, the new version of MBGC improves the default mode ratio by an average of 12% (without sacrificing speed, and beating MBGC1 max mode in most cases), and in the max mode it gains an additional 11% at the cost of almost doubling the compression time.

Table 5 presents results for compressing collections of whole *H. sapiens* genomes stored in separate FASTA files. Such representation requires a large dictionary to find distant LZ-matches between genomes. General-purpose compressors and Genozip could not exceed 5-fold compression ratio. Removal of EOL symbols from chromosome sequences did not result in a drastic improvement for universal compressors. AGC and MBGC2 in their default mode produce archives of comparable size. Nearly twice the reduction in memory usage and two times faster decompression of HGSVCp dataset make AGC the winner. Note that Jarvis3 results were ignored due to erroneous compression and decompression, similarly to the case of the large bacterial collection (cf. Table 2).

For the next experiment, we took two yeast genome collections, *S. cerevisiae* and *S. paradoxus* (Table 6). Since AGC fails to compress files with the same name, it was necessary to rename `genome.fa` FASTA in folders representing yeast strains prior to the experiments. MBGC2 and GDC 2 are superior in the compression ratio. In terms of ratio, the next tool is MBGC1, succeeded by NAF and AGC (in the default mode), the latter being quite fast. In decompression, AGC is the fastest, followed by zstd, then by MGBC2 (default and repo modes), and then by GDC 2. The remaining tools are noticeably slower, especially Jarvis3.

In the following experiments (Tables 7–9), tools compress collections of much shorter sequences stored in individual files (Multi-Multi-FASTA) and include RNA, protein, and DNA data ranging in size from 73MB to nearly 19 GB. MBGC is able to compress such FASTA, via the `-i` switch, which is useful if the input is stored in such form. Note that AGC has a dedicated -c option for Multi-Multi-FASTA processing, but then it decompresses sequences in the lexicographic order of their identifiers, which does not suit our usage scenario. Preliminary experiments have shown that using this option has an ambiguous effect on AGC performance. The tool does not provide lossless compression of protein datasets, since it supports only IUPAC nucleotide codes in the input data. AGC requires a separate reference dataset, i.e., a separate file. To test it, we created reference files from the first contigs of the compressed files and applied adaptive mode (option -a). Noteworthy are the high compression ratios achieved by general-purpose compression tools despite such diverse data. In half of the cases (4 out of 8 datasets), the best ratios were reached by either 7z or BSC after EOL symbols were removed from sequences. The most spectacular result belongs to BSC, which compressed the 18.8 GB COVID collection to 13.8 MB. The single-file experiments show a weaker spot of MBGC2, which is not among the tools with the best ratios (usually, Genozip in its strongest mode). On the other hand, MBGC is among the faster contenders in compression; it is usually beaten by NAF and zstd in mode `-3`, which are not superior in the compression ratio. To perform well, AGC requires parameters to be adjusted for a given dataset. In extreme cases, the difference in ratio can be 4-fold (see Influenza results in Table 9). On the other hand, tuning the compression ratio may sometimes result in a rather drastic, up to two

[1]https://github.com/cobilab/humangenome

Table 5: Compression results – collections of human genomes.

	HGSVCp (36 genomes, 104.38 GB)					HGSVCu (36 genomes, 102.88 GB)					HPRC (95 genomes, 290.13 GB)				
	ratio	ctime	dtime	cmem	dmem	ratio	ctime	dtime	cmem	dmem	ratio	ctime	dtime	cmem	dmem
BSC -p -b2047	4.4	989.1	576.4	128.85	128.84	4.4	921.0	593.6	128.84	128.84	4.6	2450.9	945.3	128.84	128.84
7z -md4g	4.9	59302.0	316.1	125.49	17.59	5.0	69605.0	282.1	125.89	20.72	—	—	—	—	—
zstd -3 –long=31	3.3	(1)191.2	158.7	(2)2.35	(1)2.15	3.3	(1)180.7	154.9	(2)2.35	(1)2.15	3.4	(3)514.9	466.5	(2)2.35	(1)2.15
zstd -19 –long=31	4.1	9465.0	170.0	5.08	(1)2.15	4.2	9408.0	157.3	5.08	(1)2.15	4.4	25549.0	410.0	(3)5.10	(1)2.15
Genozip default	4.6	553.8	141.8	4.75	4.22	4.5	502.6	134.7	5.40	4.37	4.8	1355.5	548.9	5.54	3.62
Genozip -b best	4.7	638.6	136.7	102.59	67.47	4.7	645.5	175.8	101.31	67.33	4.9	1554.5	445.4	99.34	66.68
NAF -3 –long=31	3.8	846.3	392.6	(1)2.30	(1)2.15	4.2	740.0	337.2	(1)2.30	(1)2.15	4.1	2246.5	1018.8	(1)2.31	(1)2.15
NAF -19 –long=31	4.7	56246.0	383.2	(3)2.64	(1)2.15	5.2	49906.0	333.2	(3)2.64	(1)2.15	—	—	—	—	—
AGC default	89.9	(2)208.5	(1)53.6	24.07	21.36	96.6	(2)194.0	(1)52.9	24.14	16.53	(2)201.3	539.0	(2)157.3	27.69	23.33
MBGC1 max	(3)95.4	653.0	150.8	49.10	44.45	101.2	634.0	141.0	40.72	33.88	160.6	1611.3	398.6	42.49	40.58
MBGC2 default	95.1	(3)222.7	(3)105.2	47.16	33.24	(3)101.4	(3)205.4	(3)82.1	46.41	37.00	180.5	(1)248.2	(3)162.7	51.67	43.28
MBGC2 repo	(2)97.3	441.2	(2)93.9	60.33	34.74	(2)103.3	269.1	(2)71.3	56.19	38.16	(3)188.1	(2)386.3	(1)154.3	59.56	43.40
MBGC2 max	(1)106.2	628.7	135.9	47.36	33.92	(1)115.2	631.4	106.2	38.48	36.94	(1)208.1	1499.4	199.3	42.31	29.70
BSC* -p -b2047	4.8	923.5	301.7	128.84	128.84	4.8	920.1	302.4	128.84	128.84	5.0	2382.6	791.3	128.84	128.84
7z* -md4g	**5.6**	67557.0	269.0	125.65	20.38	**5.7**	65563.0	261.9	124.83	20.09	—	—	—	—	—
zstd* -3 –long=31	4.0	**175.7**	**147.8**	**2.36**	**2.15**	4.1	**189.0**	**147.0**	**2.35**	**2.15**	4.2	**542.6**	442.7	**2.36**	**2.15**
zstd* -19 –long=31	5.0	9053.0	149.2	5.02	**2.15**	5.1	9006.0	151.0	5.02	**2.15**	**5.2**	24220.0	**367.8**	5.04	**2.15**

The best three results are marked with a number in parentheses.
7z and NAF failed to compress the largest dataset within the 100,000 seconds limit (denoted with "—").
MBGC1 in the default mode failed to compress the datasets due to critical errors.
[1] AGC converts bases to uppercase, resulting in lossy compression of the HGSVCp dataset.
* The results obtained with the general-purpose tools in the bottom four rows concern the scenario in which End-Of-Line (EOL) symbols were removed from the DNA strings in the input files prior to the experiment. The best results in columns are marked in bold.

orders of magnitude, decompression slowdown (see Table 7). The current MBGC2 version improves decompression speed for protein datasets ∼2.5 times on average (cf. Table 8).

In the experiments above, the speed of BSC and 7z was hampered by limiting the number of threads in order to maximize the compression ratio without exceeding the available system memory. Tables 11 – 13 provide benchmark scenarios where tools take advantage of comparable computational resources. Additionally, here we present the average CPU thread utilization given in percentage, shown in rows as "ccpu" for compression and "dcpu" for decompression. NAF is a single-threaded tool; therefore, its results do not change in all of these experiments. In Table 11 (resp. Table 12), BSC, zstd, Genozip, AGC, and MBGC2 are confined to use only 1 thread (resp. 6 threads) for compression and decompression. In the Table 12, 7z runs with our default configuration used in previous tests. As expected, compression time increased noticeably for the hampered tools. The exception in Table 12 is MBGC2 and zstd in the fast mode (i.e., `-3`) which modestly utilize CPU threads only up to 416% and 419%, respectively. All tools maintain a stable ratio, except for MBGC, which, in default mode, compresses noticeably more strongly with a single-thread limitation. The reason for this is that in MBGC, it is not possible to search for matches in genomes processed in parallel. Note that zstd, AGC and Genozip do not provide strict thread control, as the set number was occasionally exceeded. In the experiment presented in Table 13, all tools were executed in their standard configurations without imposing any limits on threads, and parameters of BSC and 7z were adjusted to ensure the best possible ratio. The acceleration of 7z compression is more pronounced than that of BSC. The impact of threads on decompression speed is small in general because most tools (except for BSC and Genozip) rarely use more than a few CPU threads. In rare cases, limiting the number of threads even boosts decompression.

MBGC2 allows for extraction of only selected files (matching a given set of patterns), but does not offer random access to the compressed collection. However, it takes less time to extract a genome near the beginning of the archive than to extract a genome near the end. On the other hand, AGC provides random access to individual genomes and is a more suitable tool to work in such a scenario, as it typically extracts a single file an order of magnitude faster than MBGC2. In Fig. 1 we show the running times required to decompress more than one file at a time for both tools. For small (resp. large) pathogen collections, MBGC2 was usually faster when >10% (resp. >2%) of the files were extracted. For human genome collections, AGC outperformed MBGC2, being ∼10–30 times faster at decompressing any portion of a dataset smaller than 10%. Note that AGC was unable to decompress >80,000 bacterial genomes (cf. mixed pathogens plot) with a single command due to an overly long list of arguments (all output files must be specified as command line arguments). AGC also failed to decompress half of HPRC dataset throwing an error `"Command terminated by signal 11"`.

MBGC2 can now extend an existing archive with new FASTA files without consuming disk space for temporary storage.

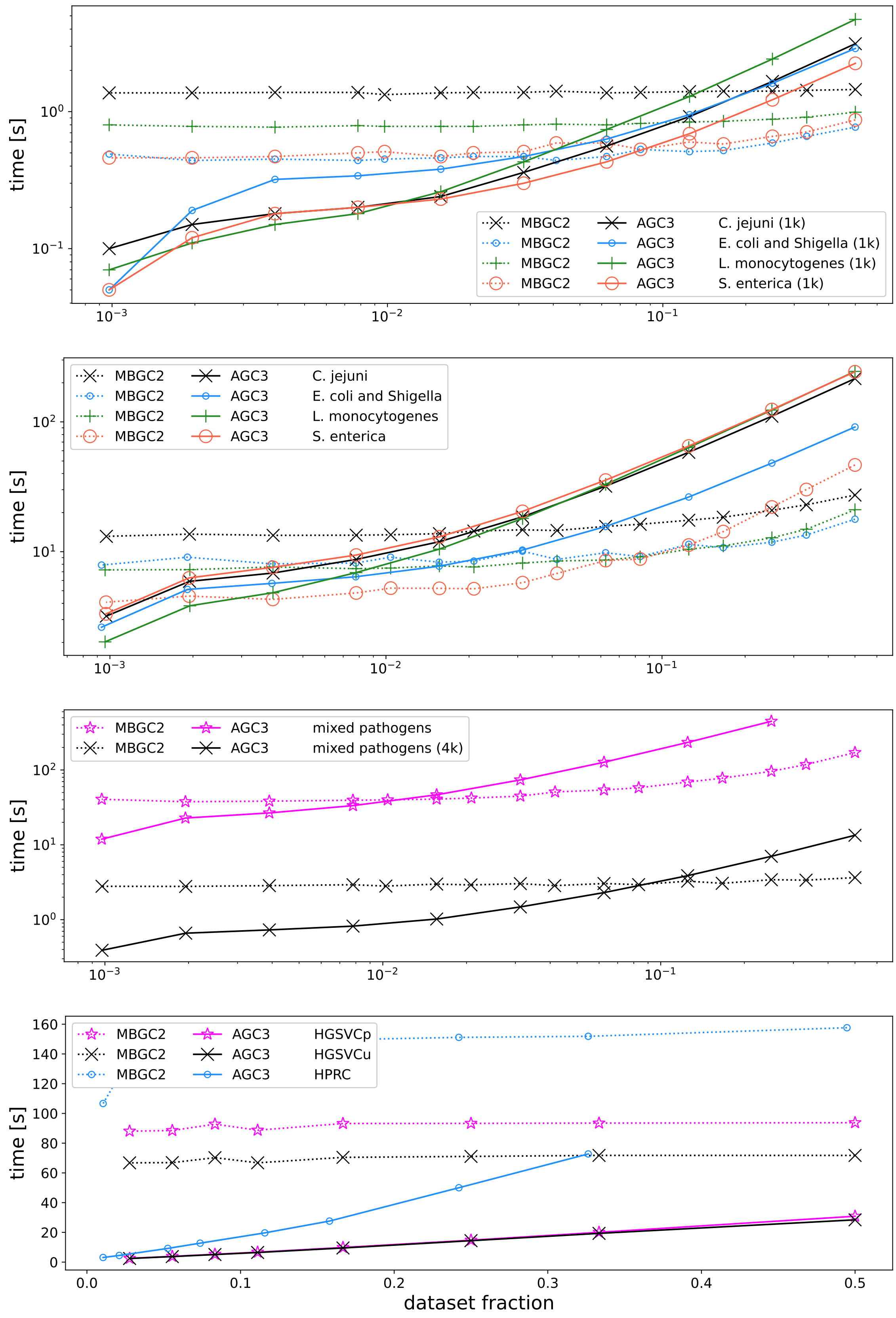

Figure 1: Time of a dataset fraction decompression. Files are selected by evenly sampling all content, e.g., when decompressing 0.125 fraction of a dataset files 1, 9, 17, 25, etc. are selected (according to the order of compression).

Table 6: Compression results – yeast collection.

	S. cerevisiae (39 genomes, 493.98 MB)					S. paradoxus (36 genomes, 436.43 MB)				
	ratio	ctime	dtime	cmem	dmem	ratio	ctime	dtime	cmem	dmem
GDC 2	(1)109.8	3.78	0.56	(2)0.52	(1)0.15	(3)80.7	20.52	0.83	(3)0.52	(1)0.18
BSC -p -b2047	12.1	9.75	2.72	2.54	2.48	10.2	8.73	2.70	2.25	2.20
7z -md4g	39.6	389.24	1.13	5.00	0.51	34.3	354.46	1.07	4.48	0.45
zstd -3 –long=31	4.2	(2)0.90	0.56	(3)0.56	0.50	3.8	(1)0.71	0.52	(2)0.50	0.44
zstd -19 –long=31	23.5	76.60	(3)0.38	1.55	0.50	17.3	77.98	(4)0.38	1.40	0.44
Genozip default	5.0	4.05	1.11	3.85	1.89	4.9	3.80	1.04	3.42	1.66
Genozip -b best	35.4	49.41	3.56	3.43	1.68	27.8	39.53	3.20	3.08	1.50
Jarvis3	49.9	28.38	22.87	(1)0.34	(5)0.34	43.9	25.26	22.50	(1)0.33	(5)0.33
NAF -3 –long=31	67.0	2.61	1.04	(5)0.64	0.49	43.2	2.33	0.92	(4)0.58	0.43
NAF -19 –long=31	77.0	26.94	1.03	0.97	0.49	43.2	29.84	0.93	0.92	0.43
AGC default	70.3	1.50	(2)0.32	(4)0.59	(4)0.33	43.4	(4)1.63	(1)0.28	0.80	(3)0.32
AGC -a	70.1	(5)1.49	(1)0.31	0.74	(3)0.32	42.8	1.71	(2)0.29	0.97	(3)0.32
AGC -a -b500 -s1500	60.0	1.59	(4)0.39	0.71	(2)0.21	39.9	(3)1.48	(3)0.34	(5)0.69	(2)0.23
MBGC1 default	86.6	(3)1.21	0.75	1.94	0.81	43.9	(5)1.64	1.06	1.94	0.75
MBGC1 max	(5)91.0	3.07	0.92	1.49	0.88	(5)61.4	3.00	1.09	1.42	0.76
MBGC2 default	(4)101.5	(1)0.88	(5)0.47	1.84	0.53	(4)76.4	(2)1.36	0.54	1.82	0.51
MBGC2 repo	(3)104.2	(4)1.45	0.51	1.94	0.68	(2)82.8	1.66	(5)0.49	1.93	0.63
MBGC2 max	(2)105.5	2.69	0.75	1.40	0.75	(1)83.6	2.82	0.81	1.42	0.74
BSC* -p -b2047	52.9	8.52	2.33	2.50	2.44	33.8	7.85	2.36	2.21	2.16
7z* -md4g	**101.9**	275.08	0.66	4.92	0.50	**84.3**	257.57	0.62	4.41	0.44
zstd* -3 –long=31	48.6	**1.41**	**0.34**	**0.55**	**0.49**	31.1	**1.15**	0.32	**0.49**	**0.43**
zstd* -19 –long=31	95.8	47.25	**0.34**	1.47	**0.49**	72.1	54.01	**0.31**	1.32	**0.43**

Table 7: Compression results for single-file inputs (single Multi-Multi-FASTA); RNA data

	SILVA 132 LSURef (0.61 GB)					SILVA 132 SSURef (3.28 GB)				
	ratio	ctime	dtime	cmem	dmem	ratio	ctime	dtime	cmem	dmem
BSC -p -b2047	36.71	11.33	3.71	3.14	3.06	(4)37.49	100.37	26.40	16.42	16.43
7z -md4g	39.51	148.79	1.13	6.58	0.63	32.77	951.62	6.39	33.85	3.39
zstd -3 –long=31	17.85	(1)2.51	(2)0.37	(2)0.70	0.62	15.51	(1)14.85	(3)2.09	(3)2.34	(5)2.15
zstd -19 –long=31	37.20	28.68	(1)0.34	2.05	0.62	32.44	167.61	(1)1.72	(5)4.75	(5)2.15
Genozip default	37.31	18.91	(5)1.07	9.49	1.43	33.97	111.00	(2)1.97	14.21	2.67
Genozip -b best	(1)51.98	207.56	1.93	8.09	1.34	(1)42.74	316.30	3.59	39.36	6.72
Jarvis3	10.90	77.49	23.41	(1)0.09	(1)0.09	9.69	415.61	99.23	(1)0.10	(1)0.09
NAF -3 –long=31	31.90	(2)2.68	(4)0.52	(3)0.74	(5)0.61	25.92	(3)16.69	(5)2.87	(2)2.31	2.45
NAF -19 –long=31	41.49	43.78	(3)0.50	(4)1.08	(5)0.61	34.97	421.16	(4)2.72	(4)2.66	2.45
AGC -a	12.71	40.42	2.20	1.66	1.47	11.56	7839.00	16.34	8.92	8.59
AGC -a -b500 -s1500	22.91	62.65	8.61	2.09	1.39	16.94	8201.00	66.14	9.14	7.66
AGC -a -s3000 -b10000	28.39	60.19	154.97	1.95	1.73	22.81	7820.00	1957.78	9.70	7.88
MBGC1 default	37.91	(3)2.90	2.24	1.37	0.67	31.45	(2)15.73	14.34	5.51	2.49
MBGC1 max	(4)45.54	5.43	2.24	1.23	0.99	34.06	44.17	14.60	5.41	4.93
MBGC2 default	(5)44.49	(4)2.95	1.61	(5)1.21	(2)0.50	(5)36.99	(4)20.79	10.23	5.41	(4)2.00
MBGC2 repo	(3)47.24	(5)5.02	1.64	1.27	(4)0.55	(2)37.83	(5)43.14	10.62	5.41	(3)1.99
MBGC2 max	(2)50.82	5.33	2.28	1.22	(3)0.52	(3)37.62	43.47	14.11	5.42	(2)1.83
BSC* -p -b2047	51.47	10.91	3.64	3.10	3.03	**54.59**	97.29	25.00	16.24	16.25
7z* -md4g	**52.22**	114.70	1.00	6.52	0.62	42.08	724.41	5.52	33.52	3.33
zstd* -3 –long=31	29.08	**2.40**	0.32	**0.69**	**0.61**	24.34	**15.06**	1.66	**2.32**	**2.15**
zstd* -19 –long=31	50.83	21.78	**0.30**	1.95	**0.61**	45.25	139.52	**1.46**	4.71	**2.15**

The best five results are marked with a number in parentheses.
* The results obtained with the general-purpose tools in the bottom four rows concern the scenario in which End-Of-Line (EOL) symbols were removed from the DNA strings in the input files prior to the experiment. The best results in columns are marked in bold.

Table 8: Compression results for single-file inputs (single Multi-Multi-FASTA); protein data

	GRCh38 peptides all (0.07 GB)					PDB (0.10 GB)					UniProtKB (Swiss-Prot) (0.28 GB)				
	ratio	ctime	dtime	cmem	dmem	ratio	ctime	dtime	cmem	dmem	ratio	ctime	dtime	cmem	dmem
BSC -p -b2047	5.37	$^{(3)}$1.74	0.47	0.39	0.38	5.87	$^{(3)}$2.31	0.60	0.51	0.50	3.32	$^{(3)}$6.88	2.28	1.45	1.41
7z -md4g	7.64	14.96	0.41	0.80	0.09	$^{(2)}$7.07	25.55	0.55	1.03	0.12	$^{(4)}$4.54	119.04	2.42	3.07	0.35
zstd -3 –long=31	6.00	$^{(1)}$0.27	$^{(1)}$0.07	$^{(1)}$0.11	$^{(1)}$0.08	3.91	$^{(1)}$0.28	$^{(1)}$0.11	$^{(1)}$0.13	$^{(1)}$0.10	3.65	$^{(1)}$0.69	$^{(1)}$0.29	$^{(2)}$0.35	$^{(1)}$0.28
zstd -19 –long=31	7.18	21.19	$^{(2)}$0.08	$^{(4)}$0.34	$^{(1)}$0.08	6.66	30.55	$^{(2)}$0.12	$^{(4)}$0.37	$^{(1)}$0.10	4.38	41.40	$^{(2)}$0.30	1.07	$^{(3)}$0.29
Genozip default	7.70	2.85	0.68	1.27	0.27	4.85	8.80	0.43	1.30	0.17	$^{(2)}$4.84	$^{(4)}$8.75	1.10	4.12	0.84
Genozip -b best	$^{(1)}$8.71	29.67	0.68	0.94	0.19	$^{(1)}$7.25	53.07	0.76	1.18	0.15	$^{(1)}$4.90	178.91	3.45	3.41	0.68
Jarvis3	4.54	7.50	2.12	$^{(3)}$0.27	0.27	3.50	8.90	2.69	$^{(3)}$0.27	0.27	3.12	29.25	10.88	$^{(1)}$0.28	$^{(1)}$0.28
NAF -3 –long=31	7.24	$^{(2)}$0.43	$^{(3)}$0.11	$^{(2)}$0.20	$^{(1)}$0.08	4.86	$^{(2)}$0.62	$^{(3)}$0.16	$^{(2)}$0.19	$^{(1)}$0.10	4.13	$^{(2)}$1.91	$^{(3)}$0.42	$^{(3)}$0.36	$^{(3)}$0.29
NAF -19 –long=31	7.81	19.95	$^{(4)}$0.13	0.54	$^{(1)}$0.08	6.47	36.24	$^{(4)}$0.18	0.54	$^{(1)}$0.10	$^{(3)}$4.55	94.08	$^{(4)}$0.47	$^{(4)}$0.71	$^{(3)}$0.29
MBGC1 default	$^{(4)}$7.88	2.66	2.45	0.65	0.67	6.64	4.12	4.13	0.69	0.66	4.01	18.30	24.72	1.45	1.56
MBGC1 max	$^{(2)}$8.05	4.08	3.64	0.39	0.43	6.71	5.52	4.87	0.56	0.59	4.02	35.79	30.96	1.13	1.24
MBGC2 default	7.71	2.33	1.10	0.73	0.39	6.83	$^{(4)}$2.78	2.20	0.85	0.56	4.17	25.75	4.26	2.02	1.15
MBGC2 repo	7.74	$^{(4)}$2.06	0.96	0.83	0.38	$^{(4)}$6.84	3.04	2.21	0.86	0.57	4.18	22.14	3.94	2.06	1.11
MBGC2 max	$^{(3)}$8.04	3.95	1.59	0.38	0.31	$^{(3)}$6.88	5.70	3.74	0.48	0.36	4.41	43.03	4.81	1.00	0.86
BSC* -p -b2047	5.96	1.64	0.46	0.38	0.38	6.12	2.25	0.59	0.51	0.50	3.45	6.76	2.27	1.43	1.40
7z* -md4g	**8.32**	11.58	0.40	0.80	0.09	**7.39**	24.31	0.54	1.02	0.12	**4.77**	116.39	2.33	3.05	0.34
zstd* -3 –long=31	6.63	**0.21**	**0.07**	**0.11**	**0.08**	4.39	**0.29**	**0.11**	**0.14**	**0.10**	3.80	**0.71**	**0.29**	**0.35**	**0.28**
zstd* -19 –long=31	7.77	19.89	0.08	0.34	**0.08**	7.00	30.34	**0.11**	0.37	**0.10**	4.58	42.01	**0.29**	1.07	**0.28**

The best four results are marked with a number in parentheses.
* The results obtained with the general-purpose tools in the bottom four rows concern the scenario in which End-Of-Line (EOL) symbols were removed from the DNA strings in the input files prior to the experiment. The best results in columns are marked in bold.

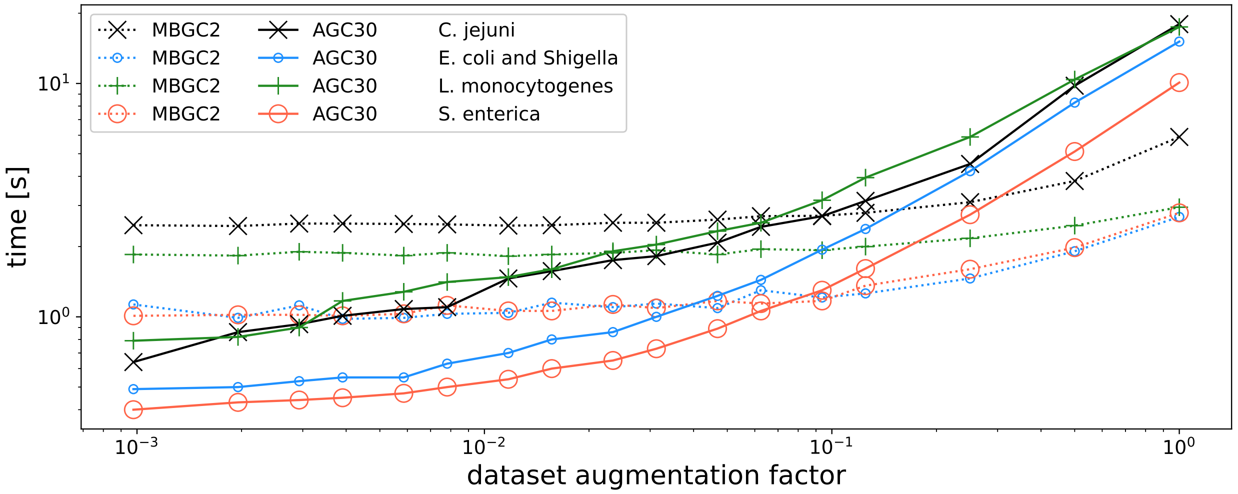

Figure 2: Time of appending an archive. The input archives for each dataset contain 1024 files. X axes indicate a number of files added relatively to the input size, e.g., 0.125 dataset augmentation factor means adding 128 files to an archive.

By organizing the archive for random access, AGC is faster when a single sample is added (cf. Fig. 2). For small pathogen collections, this operation takes up to 4 times more time for MBGC2. However, if samples are added in batches >10% of the collection size, MBGC2 takes the lead. Note that in this experiment we used the previous version of AGC tool - v3.0 - because the current version 3.2.1 failed to append more than a few files at once. Adding more than 4 files caused an error: `"Command terminated by signal 11"`.

4.1 Phylogenetic compression

Compression and decompression of massive datasets, such as the 661k collection, requires workstations equipped with large amounts of memory and disk space (cf. Table 4). In addition, with a multitude of files, archive management and access

Table 9: Compression results for single-file inputs (single Multi-Multi-FASTA); DNA data

	Influenza (1.43 GB)					Mitochondrion 1 (231.28 MB)					COVID (620k, 18.83 GB)				
	ratio	ctime	dtime	cmem	dmem	ratio	ctime	dtime	cmem	dmem	ratio	ctime	dtime	cmem	dmem
BSC -p -b2047	(4)67.58	26.06	6.67	7.33	7.16	5.03	(5)5.05	1.62	1.20	1.17	(2)885.41	140.70	30.20	94.17	94.20
7z -md4g	63.39	239.93	2.12	15.02	1.46	(5)6.63	177.26	1.46	2.36	0.27	555.19	1255.04	14.32	122.46	18.87
zstd -3 –long=31	35.38	(3)6.39	(2)0.69	(3)1.59	(5)1.43	3.51	(1)0.44	(2)0.31	(1)0.28	(3)0.24	354.28	(5)42.35	(2)7.92	(2)2.31	(5)2.15
zstd -19 –long=31	(5)65.43	52.23	(1)0.63	3.79	(5)1.43	5.59	64.95	(1)0.29	(5)0.77	(3)0.24	530.61	145.21	(1)7.69	(5)4.65	(5)2.15
Genozip default	(2)92.94	50.68	(5)1.36	17.63	3.03	5.04	(3)2.13	1.09	2.82	1.03	297.15	109.43	(5)10.13	7.22	3.77
Genozip -b best	(1)114.98	224.29	2.43	17.93	2.97	5.98	21.10	2.77	2.34	0.85	410.71	143.18	16.55	105.87	62.26
Jarvis3	41.86	77.50	29.94	(1)0.33	(1)0.33	(1)8.02	25.19	25.80	(2)0.33	0.33	274.85	1018.65	254.41	(1)0.30	(1)0.30
NAF -3 –long=31	59.58	(2)6.23	(4)1.11	(2)1.46	(5)1.43	4.45	(2)1.57	(3)0.35	(3)0.38	(1)0.23	412.55	58.46	(3)9.97	(2)2.31	2.24
NAF -19 –long=31	(3)74.74	121.01	(3)1.09	(4)1.81	(5)1.43	6.19	102.79	(3)0.35	(4)0.72	(1)0.23	519.14	127.59	(4)9.98	(4)2.65	2.24
AGC -a	10.18	913.09	5.90	3.85	2.77	4.84	5.65	0.75	1.03	0.52	501.63	1990.31	15.59	22.24	41.09
AGC -a -b500 -s1500	26.74	10315.00	14.77	9.03	3.71	3.48	5.72	0.73	1.29	0.67	(3)716.01	15805.00	27.56	24.59	22.53
AGC -a -s3000 -b10000	40.11	957.96	858.58	4.48	4.84	4.00	(4)3.23	(5)0.61	1.04	(5)0.26	(1)914.22	8434.00	186.57	22.15	41.57
MBGC1 default	45.24	(1)5.78	5.54	3.14	1.65	5.70	6.85	3.05	1.19	0.82	510.85	(3)15.42	13.76	21.34	2.18
MBGC1 max	52.58	12.60	5.16	2.60	2.31	6.01	13.61	5.02	1.10	0.86	529.40	43.76	25.24	21.46	28.60
MBGC2 default	53.69	(4)6.48	3.83	(5)2.48	(3)1.08	(4)7.02	6.41	2.19	1.15	0.71	590.52	(1)13.24	13.62	20.04	(2)0.42
MBGC2 repo	55.79	12.61	3.98	2.76	(4)1.35	(3)7.16	11.35	1.80	1.16	0.72	(5)594.50	(2)13.68	13.58	20.21	(3)0.51
MBGC2 max	60.71	(5)11.78	5.02	2.55	(2)0.96	(2)7.60	13.50	3.19	1.02	0.84	(4)617.29	(4)29.18	14.15	19.80	(4)0.56
BSC* -p -b2047	**87.02**	25.63	6.55	7.23	7.07	5.82	4.86	1.49	1.18	1.15	**1369.30**	137.53	29.99	92.85	92.88
7z* -md4g	69.77	215.29	2.01	14.86	1.44	**7.93**	160.04	1.29	2.34	0.26	605.34	1156.05	13.77	122.45	18.61
zstd* -3 –long=31	45.10	**6.47**	0.64	**1.57**	**1.42**	4.37	**0.50**	**0.28**	**0.28**	**0.23**	421.15	**39.95**	7.39	**2.31**	**2.15**
zstd* -19 –long=31	76.83	46.98	**0.61**	3.75	**1.42**	6.74	61.37	**0.28**	0.76	**0.23**	627.78	161.49	**7.38**	4.64	**2.15**

The best five results are marked with a number in parentheses.
* The results obtained with the general-purpose tools in the bottom four rows concern the scenario in which End-Of-Line (EOL) symbols were removed from the DNA strings in the input files prior to the experiment. The best results in columns are marked in bold.

to individual sequences become difficult. To mitigate these issues, the work (Břinda *et al.*, 2025) presents an effective approach in which larger collections are divided into smaller ones according to phylogenetic relationships between genomes. The effect of applying this method can be observed in the example of 4 × 1024 bacterial dataset presented in Table 2. Dividing the datasets into four species (i.e., C. jejuni, E. coli, L. monocyt., S. enterica) improves the compression ratio in the default mode by 5% (cf. Table 1). At the same time, peak memory consumption reduces by 42%. In (Břinda *et al.*, 2025) phylogenetic compression, implemented in the MiniPhy tool, was applied to the 661k collection using either xz or MBGC as a low-level compressor. First, the collection was divided into clusters by metagenomic classification. Then, too large clusters of oversampled pathogens (e.g., S. enterica and E. coli) were further split into batches to balance demand on processing resources. On the other hand, small clusters were merged and formed pseudo-clusters, called dustbins. Next, the samples in each cluster were reordered based on rapidly estimated compressive phylogenies and then compressed. The results[2] show that using the general-purpose compressor xz, this approach achieves a compression ratio of around 90. This is competitive with specialized compressors such as AGC and MBGC (cf. Table 4). Still, in addition to phylogenetic compression, it was crucial to preprocess FASTA by stripping EOL symbols within sequences. Furthermore, xz is slow in compression (despite rapid decompression) and requires two orders of magnitude more time than MBGC2. Experiments confirm that the phylogenetic approach has a positive impact on MBGC2 compression ratio, improving the performance of max mode by ∼6.8% (from 141.6 up to 151.2 – cf. Table 4 and (Břinda *et al.*, 2025)). Unlike the typical way of using MBGC, batches were converted to single FASTA files prior to compression. Hence, we performed additional tests with batches given as a collection of FASTA files, which resulted in boosting ratio even further up to 152.3. An attempt to compress files in batches according to lexical order caused a drastic decrease in the ratio to 138.3, which confirms that reordering of genomes plays a significant role in the phylogenetic compression. The largest decreases, in some cases exceeding 20%, were observed on dustbin batches. It should be noted that reordering usually takes several times longer than compression with MBGC2. We had also repacked the reordered batches using the default and repo modes, achieving ratios of 125.2 and 148.2, respectively. In the latter case, the gain exceeded 25% (cf. Table 4). The reason for this may be that matches between sequences in repo mode may refer more often to neighboring genomes (see Section 5).

[2]https://brinda.eu/mof/

5 Changes in MBGC2

In addition to several performance and functionality improvements of our tool, MBGC2 fixes the issues reported in (Deorowicz *et al.*, 2023) that prevented MBGC1 from successfully handling huge collections (see Table 4) and FASTA files (see Table 5).

The functions added to MBGC2 aim to increase tool usability in various scenarios:

- quick listing filenames & identifiers,
- more flexible partial decompression by specifying a list of file name patterns,
- complete or partial (by specifying a list of file name patterns) archive repacking (MBGC 2 is backward compatible and can handle decompression and repacking of MBGC 1 archives),
- appending MBGC2 archives with new files,
- decompression to gz archives,
- preserving the length of DNA lines in FASTA files (only if the line length is consistent throughout the file).

To demonstrate the performance of MBGC2, let us look at the average values of the default and max modes (given relative to MBGC 1.2.2) from experiments conducted on bacteria collections used in (Grabowski and Kowalski, 2022). MBGC2 improved compression ratio by 14% without sacrificing speed. In fact, the compression acceleration in the max (resp. default) mode is 55% (resp. 14%). The improvement is mainly due to the introduction of efficient techniques for encoding approximate matches between sequences and the reference buffer (MBGC1 only considered MEMs, i.e., maximal exact matches). The effect of each new technique is shown in Table 10. More details on those techniques are presented in Section 5.2. Additionally, in the max mode we applied RC redundancy elimination to the literals stream. What we mean here is reverse-complemented matches of sequences. An efficient search for such RC-matches is done using a hash table and sparse sampling technique (Grabowski and Bieniecki, 2019). However, a noticeable improvement (1–2 %) occurs only for the human genome collections.

Decompression in MBGC1 used multithreading to parallelize backend extraction (e.g., with LZMA or PPMd) of streams and by overlapping I/O operations with genome decoding, which was performed by a single thread. MBGC2 has been reengineered to use threads to decode different genomes and offers ∼40% faster decompression. Although each genome is compressed with respect to those preceding it, quite often there are no direct dependencies between that genome and several of its closest ones. This makes parallel decoding possible. In MBGC2, after the first decoding thread is started, the second thread determines the dependencies between genomes (more specifically, for each genome, it determines the farthest genome on which it depends). Then, additional decoding threads can be spawned. Multithreaded decoding requires more careful handling of the reference buffer. During compression, additional information about the length of the contribution of each genome to the buffer is recorded (in a separate stream). Moreover, additional separator characters were placed in the reference buffer to avoid matches that would cross the boundaries of areas related to two different genomes.

In MBGC1's default mode, the compression ratio was quite susceptible to the number of threads, e.g., in the extreme case of the small C. jejuni dataset, the compression loss was as much as ∼15% when the number of threads increased from 1 to (the default) 8. In MBGC2, this effect was reduced to <5% by implementing an encoding threads management strategy. This is accomplished by suspending the encoding thread if no matches covering at least one-eighth of a sufficiently long contig (i.e., contigs <1024 characters are not considered) are found. Once all preceding genomes except the last one have been encoded, the thread is resumed and begins re-encoding the genome against the supplemented reference buffer. The encoding threads management strategy is applied with tightened parameters (i.e., an encoding thread is suspended if $1/64^{th}$ of a contig is not covered with matches and waits until all preceding genomes are encoded) to the redesigned repo compression mode (only ∼2% worse ratio on average than the MBGC 2 max mode in ∼48% less time).

MBGC2 features smaller memory footprint (∼10% in compression, and ∼18% in decompression). One reason for this is the breakdown of MBGC products into more streams (e.g., mismatches, and gaps delta stream), which has reduced the size of the largest streams (i.e., matches offsets, and literals). Additionally, encoding improvements allowed the relaxing of parameter u, which determines the "growth rate" of the reference buffer. In MBGC2, a contig is appended to the buffer if the fraction of contig symbols not covered by matches exceeds 1/128 (i.e., parameter $u = 128$). In the case of MBGC1, this value was 1/192. To slim down the reference buffer even more, contig reverse-complements extend the buffer only if the mentioned fraction exceeds 1/8 (i.e., parameter $u_{RC} = 8$).

Table 10: Boosting compression ratio with 6 new MBGC2 techniques of encoding matches and their adjacent surroundings relative to the reference sequence and technique for elimination of RC redundancy in the literals stream (available only in MBGC2 max mode -m3). Change in ratio relative to MBGC2 max mode in percents.

	MBGC v2.0 -m3							v1.2.2
options	-R0	-b	-X	-G0	-g0	-G0 -x0	-x0 -g0 -R0	-c3
RC content in literals removal	—	✓	✓	✓	✓	✓	—	—
gaps delta encoding	✓	✓	✓	✓	—	✓	—	—
gap breaks filter	✓	—	✓	✓	—	—	—	—
encoding in gaps	✓	✓	✓	—	—	—	—	—
adjacent encoding	✓	✓	✓	✓	✓	—	—	—
mismatch encoding	✓	✓	—	✓	✓	—	—	—
C. jejuni (1,024 genomes)	-0.1	-3.6	-3.5	-0.5	-7.6	-20.5	-25.9	-25.0
E. coli (1,024 genomes)	0.0	-0.5	-0.6	-0.1	-1.8	-3.2	-4.8	-3.1
L. monocyt. (1,024 genomes)	-0.3	-4.2	-2.5	-0.6	-12.6	-18.8	-28.7	-26.9
S. enterica (1,024 genomes)	-0.1	0.2	-0.7	-0.1	-2.0	-4.6	-6.8	-7.0
mixed pathogens (4× 1,024)	-0.1	-3.1	-2.8	-0.5	-8.0	-17.7	-23.9	-23.0
C. jejuni (55,627 genomes)	-0.0	-7.9	-2.1	-0.2	-1.1	-14.4	-12.2	-10.3
E. coli (22,523 genomes)	-0.0	-6.0	-1.5	-0.3	-0.8	-9.7	-10.1	-4.7
L. monocyt. (36,448 genomes)	-0.1	-4.8	-1.5	-0.2	-0.7	-9.9	-10.2	-8.0
S. enterica (53,713 genomes)	-0.1	-5.6	-1.1	-0.2	-1.6	-7.7	-9.3	-3.3
mixed pathogens (168,311 genomes)	-0.0	-5.8	-1.7	-0.2	-1.3	-11.3	-10.3	-9.4
S. en. cluster (14,003 genomes)	-0.1	-0.3	-0.9	-0.3	-2.9	-3.4	-6.0	-4.0
S. cerevisiae (39 genomes)	0.0	-1.1	-1.0	-0.3	-8.6	-5.9	-13.8	-13.7
S. paradoxus (36 genomes)	0.0	-2.0	-2.3	-0.8	-11.4	-19.9	-27.6	-26.6
HGSVCp (36 genomes)	-1.2	-1.2	-1.4	-0.1	-4.4	-3.9	-9.9	-10.1
HGSVCu (36 genomes)	-1.0	-1.0	-1.7	-0.3	-4.3	-4.1	-9.5	-12.2
HPRC (95 genomes)	-2.0	-2.3	-2.1	-0.7	-5.3	-5.9	-13.4	-22.8
average ratio change	-0.3	-3.1	-1.7	-0.3	-4.7	-10.1	-13.9	-13.1

5.1 Backend compression

In MBGC2, we have retained the approach of compressing the by-products (streams) of our LZ77-like compression procedure at the last stage. The lzma library (`https://7-zip.org/sdk.html`) is used for all of them, and we rely on two fundamentally different algorithms it supports: LZMA (from the Lempel-Ziv'77 family) and PPMd (from the Prediction by Partial Matching family). PPMd makes use of two parameters: maximum memory usage (set to 192 MB in our application) and its maximum order. For LZMA, we use it with its maximum compression mode, with extra parameters set to improve the compression on periodical data (e.g., lp = 2, pb = 2, appropriate for 4-byte data items), and running 2 worker threads.

Two major new streams (described in the next section) needed handling at the backend. One to store the number of gaps between corresponding matches, and the other for mismatches flags. PPMd with a row of 8 (resp. 14) was used to compress the first (resp. the second) stream. Both of them were divided into 2 blocks compressed in parallel. In general, such a partitioning into blocks is not used, however, for small inputs, as the minimum length of a block is set to 2^{20} bytes. New techniques for encoding approximate matches have affected the content of the literals stream. This allowed, in its case, to speed up the PPMd encoder by reducing the order from 7 to 5. However, in the case of proteins dataset changes made in MBGC2 version had a bad effect on the literals stream compression ratio. This may be due to techniques used to encode mismatches and regions adjacent to matches, an approach that is more suitable for sets of nucleotide sequences. The current version of MBGC2, probes the small portion (up to 65536 symbols) of the reference genome sequences, and if fraction of non-DNA bases exceeds 10% it switches to the LZMA encoder, which results in a better ratio of encoded literals stream and significantly boosts overall decompression speed. Additionally, exclusive mismatches encoding matrix

is disabled and mismatched bases are stored directly.

The backend processes the streams in parallel, so the final compression or decompression time is determined by the most significant stream. Unfortunately, it cannot be indicated explicitly because it varies depending on the dataset being compressed. As the changes made in MBGC2 improved the compression ratio, we determined the settings experimentally to optimize the speed of the tool. For this purpose, we:

- increased the number of LZMA blocks (handled in parallel) for the match offsets stream from 2 to 3, so as not to hamper compression,
- increased the number of parallel blocks for the match lengths stream from 3 to 5, and switched from PPMd to LZMA algorithm for even faster decompression,
- applied processing in two parallel blocks for the sequences headers stream, which is important for the speed of handling such collections as influenza (a lot of short sequences with abundant headers).

There are a few other, minor data streams (e.g., responsible for sequence line lengths formatting or storing file names) as well. Their impact on the overall compression (and other aspects, like compression speed) is rather negligible, and for this reason, we omit presenting details of their handling.

5.2 Considerations on approximate matches encoding procedure

To help understand the internals of MBGC2, we have prepared an example (Fig. 3). It explains the basic MBGC routines such as exact matching using a sparse hash table, extending of reference buffer, and skip margin, by showing the steps, from creating REF from the first genome (G_1) to finding and expanding matches (left and right) and possibly updating REF together with the hash table. The example is too small to address the idea of *gaps delta encoding* (explained in the main paper) or *gap breaks filter*. The next figure 4 supplements the previous one with details of the techniques used to encode the area adjacent to a MEM.

In genomic data, there may be many MEMs in the approximate match area. We identify them by finding so-called *corresponding matches*, i.e., pairs of matches between which the distance in the sequence is identical to that in the reference. A pair of corresponding matches confirms that we are dealing with a long approximate match. Therefore, in the gaps between them, we do not impede adjacent encoding by the scoring routine and simply encode entire gaps using the exclusive mismatch encoding matrix. Due to the possibility of multiple indels, this procedure is not used if corresponding matches are separated by more than 2 gaps. Nevertheless, the overall gain in compression ratio is rather small and does not exceed 1% (cf. *encoding in gaps* in Table 10).

We note that MBGC2 does not handle indels explicitly. In preliminary experiments on bacteria, we tested the effect of successful detection and encoding of indels on the cost of storing match offsets in the archive. Considering indels that change the length of gaps between corresponding matches up to three bases, the overall gain in compression ratio was less than 0.2%. However, it is unclear how encoding indels would ultimately affect the speed and level of compression. Instead of directly encoding indels, MBGC2 uses mismatch encoding on both sides of the gap between consecutive matches and applies scoring to terminate the routine (e.g., when indel occurs).

References

K. Břinda, L. Lima, S. Pignotti, N. Quinones-Olvera, K. Salikhov, R. Chikhi, G. Kucherov, I. Gregory, Z. Iqbal, and M. Baym: Efficient and robust search of microbial genomes via phylogenetic compression. *Nature Methods*, **22**(4) 2025, pp. 692–697, https://doi.org/10.1038/s41592-025-02625-2.

S. Deorowicz, A. Danek, and H. Li: AGC: compact representation of assembled genomes with fast queries and updates. *Bioinformatics*, **39**(3) 2023, btad097, https://doi.org/10.1093/bioinformatics/btad097.

S. Grabowski, W. Bieniecki: copMEM: finding maximal exact matches via sampling both genomes. *Bioinformatics*, **35**(4) 2019, pp. 677–678, https://doi.org/10.1093/bioinformatics/bty670.

S. Grabowski, T. Kowalski: MBGC: Multiple Bacteria Genome Compressor. *GigaScience*, **11**, 2022, giab099, https://doi.org/10.1093/gigascience/giab099.

M. Sousa, A. Pinho, and D. Pratas: JARVIS3: an efficient encoder for genomic data. *Bioinformatics*, **40**, 2024, btae725, https://doi.org/10.1093/bioinformatics/btae725.

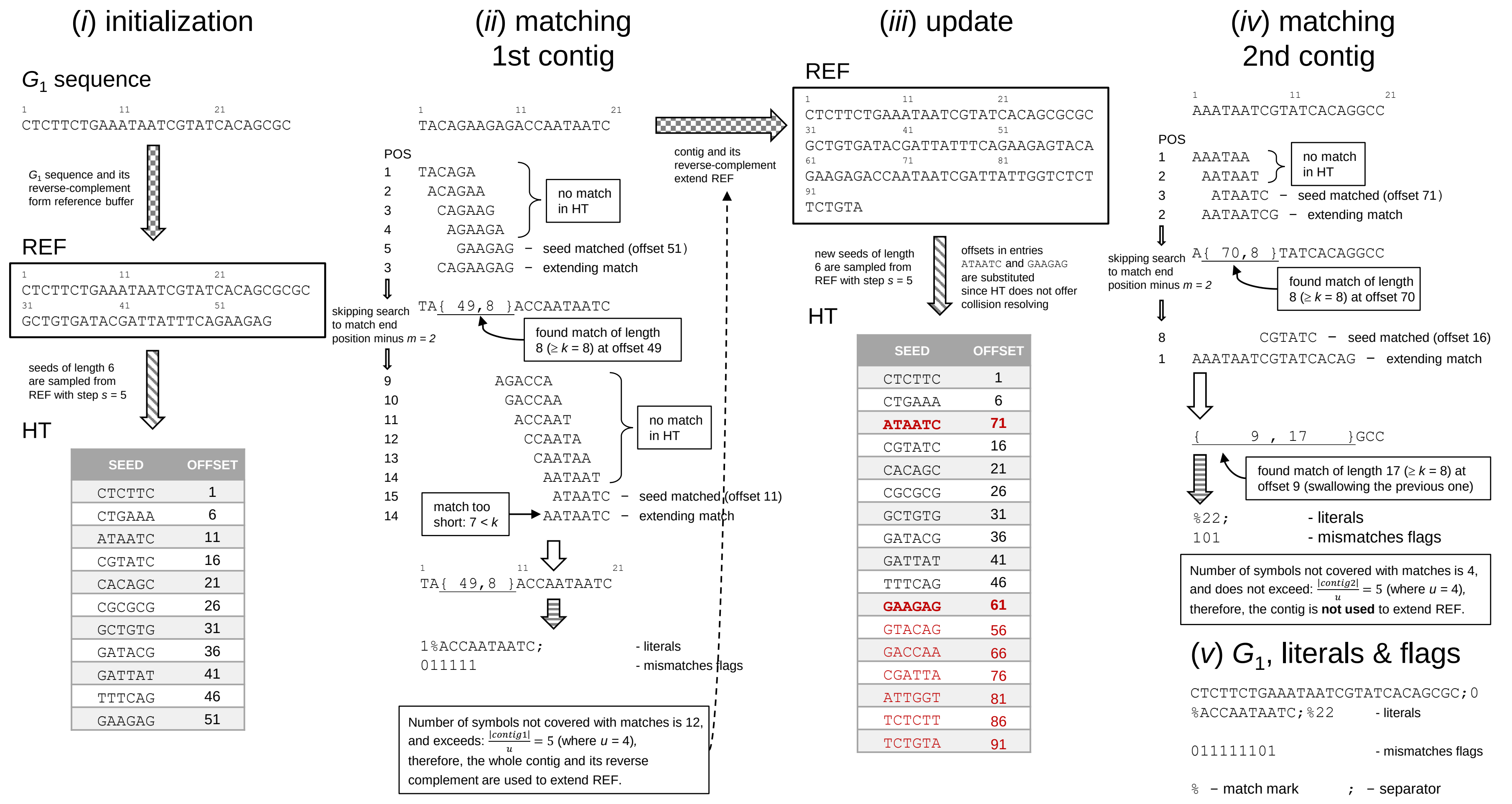

Figure 3: Steps of the MBGC2 compression, on a high level, for a toy example. The basic parameters used for this example are: seed length 6, $k = 8$, $m = 2$, $u = u_{RC} = 4$, $s = 5$. (i) REF is created from the first genome, G_1, of length 28 (REF is now the concatenation of G_1 and $rc(G_1)$, and thus twice longer), and the hash table HT is populated with seeds taken from REF. (ii) Substrings from the first contig are sought in HT, and the found matches are left- and right-extended. Note how the skip margin idea is applied. The space between matches is sent to the literals stream, but it is partially encoded relatively to the surrounding of the matches (adjacent encoding) with additional information written into the mismatches flags stream (the details are explained in Supplementary Figure 4). After the contig is processed, the decision is made if REF should be extended (affirmative here). (iii) REF is appropriately extended, and HT is updated; newer seeds overwrite older ones in case of a collision. The new seeds in HT are marked red, and additionally with a bold typeface when overwriting. (iv) Processing another contig. Note how the skip margin idea allows for swallowing the previously found match (thus reducing the number of output matches). The decision whether REF should be extended is negative this time. (v) The final stream of literals and matches, where matches are only marked with `%` symbol and contig separators are marked with `;` symbol, plus supplemental mismatches flags stream. The prefix of this stream of length 28 is G_1 genome.

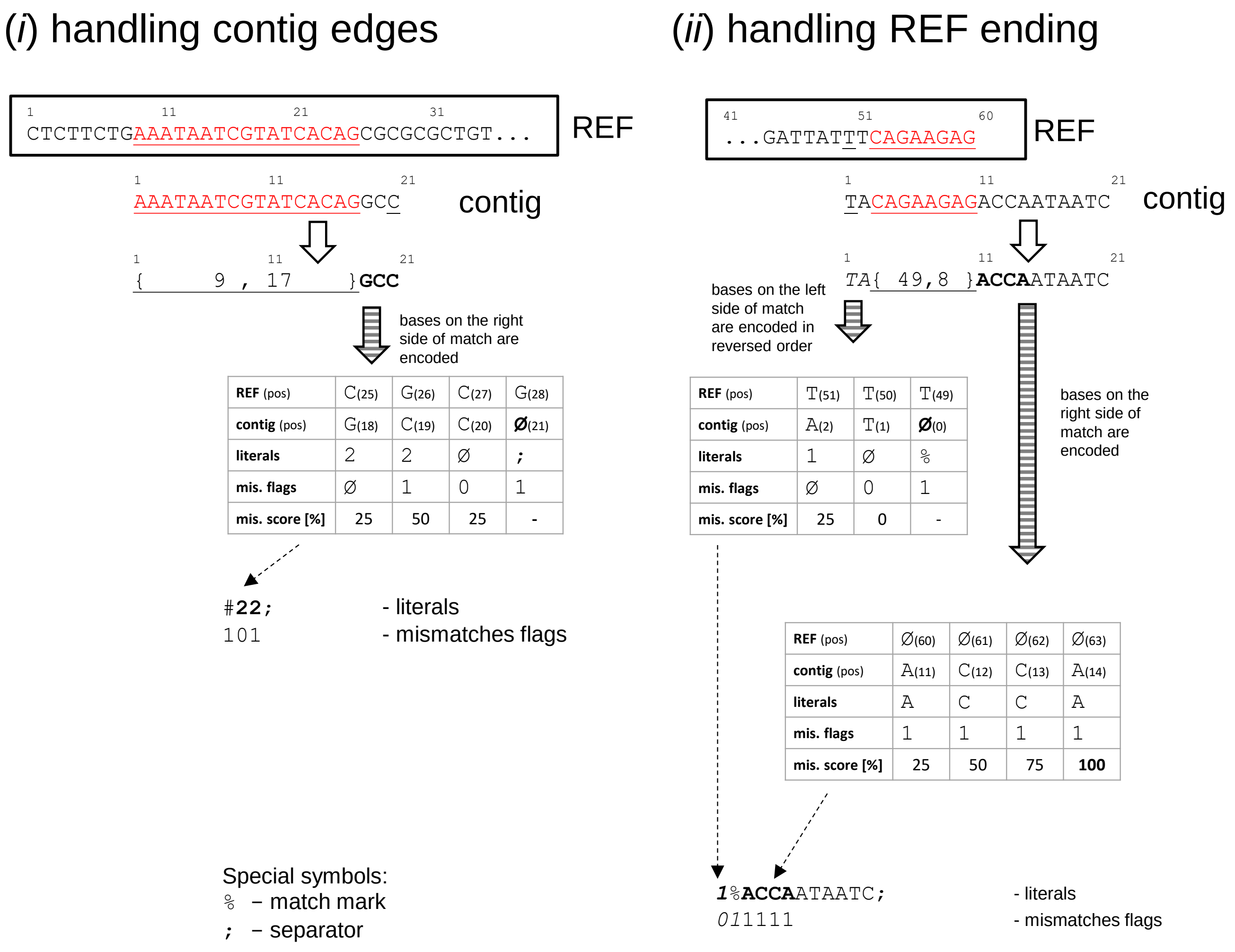

Figure 4: The encoding of mismatches adjacent to a match for a toy example. The parameters used for this example determine the behavior of the mismatch scoring routine: $x = 4$, $Y = 25\%$.
(i) The case from Figure 3(iv). 17 bases of contig prefix match REF at position 9. The encoding mismatches routine is applied only to the right side of a match. As base G directly succeeding the match is known to be a mismatch (otherwise it would be incorporated into the match), there is no need for a mismatch flag, and the base is encoded relatively to base C (26th REF position) according to exclusive mismatches encoding matrix into value 2 and pushed to literals stream. At this point, the initial matching score (25%) is set. The following bases are processed sequentially. Encoding of mismatches is terminated by reaching the end (or beginning) of the contig, a set flag is sent to the mismatches flags stream, and the contig separator to the literals stream.
(ii) The case from Figure 3(ii). Another special case. Encoding of mismatches reaches an uninitialized part of REF (the current end). Four bases are encoded as mismatches in order to terminate the mismatches routine (by reaching mismatches score limit). In literals stream simply contig bases are stored to avoid referring to an uninitialized REF region (during encoding and decoding).

Table 11: Compression results - single thread limit

		BSC -p -b2047	7z -md4g	zstd -3 –long=31	zstd -19 –long=31	Genozip default	Genozip -b best	NAF -19 –long=31	AGC best ratio[1]	MBGC2 default	MBGC2 max
	ratio	28.7	119.8	14.1	61.3	5.4	47.7	(3)214.6	172.0	(2)422.7	(1)456.8
bacteria	ctime	676.8	11112.0	(3)39.9	6304.0	1021.0	1411.2	298.6	241.3	(1)32.9	(2)35.4
mixed	dtime	193.4	18.9	(2)10.6	(1)9.4	447.8	361.2	36.2	69.9	(3)14.2	14.3
4 × 1024 gen.	cmem	10.74	40.81	2.30	3.53	(1)1.12	4.61	2.66	3.59	(2)1.90	(3)2.16
14.94 GB	dmem	10.74	4.30	2.15	2.15	(1)0.25	3.94	2.24	(2)0.27	(3)1.36	1.66
	ccpu	99	99	119	100	96	98	96	108	87	90
	dcpu	98	99	116	103	101	101	23	114	99	99
	ratio	45.4	619.4	27.5	191.2	5.4	65.6	2175.6	(3)3325.2	(2)7838.2	(1)7999.5
S. enterica	ctime	2985.1	47554.0	(3)112.6	20188.0	3722.0	4611.0	271.0	540.3	(1)60.3	(2)86.2
cluster	dtime	803.1	66.8	47.4	(3)42.5	1235.8	776.0	161.8	98.7	(1)41.5	(1)41.5
14,003 genomes	cmem	10.74	40.83	(2)2.30	3.52	(1)1.31	4.40	2.66	2.87	(3)2.42	2.43
67.12 GB	dmem	10.74	4.30	2.15	2.15	0.25	3.72	2.27	(2)0.17	(1)0.15	(3)0.18
	ccpu	99	99	122	100	96	97	82	114	78	66
	dcpu	98	99	100	90	102	103	22	132	99	99
	ratio	38.4	338.8	20.6	148.3	5.3	60.2	1024.3	(3)1314.5	(2)2237.5	(1)2252.6
S. enterica	ctime	218.0	3592.4	(3)8.7	1676.9	234.5	302.8	25.2	40.9	(2)5.1	(1)4.8
cluster part	dtime	61.5	5.5	(1)3.1	(1)3.1	53.8	20.5	11.8	6.7	(1)3.1	(1)3.1
1,024 genomes	cmem	10.75	40.81	2.30	3.53	(1)0.86	4.00	2.64	2.86	(2)1.13	(2)1.13
4.87 GB	dmem	10.74	4.30	2.15	2.15	0.20	3.61	2.16	(3)0.12	(1)0.06	(2)0.07
	ccpu	99	99	126	100	95	97	86	111	74	80
	dcpu	98	99	116	97	104	109	23	134	99	99
	ratio	4.4	—	3.3	4.2	4.5	4.7	5.2	(3)96.6	(2)109.5	(1)115.2
	ctime	6723.0	—	(1)526.0	79373.0	5177.0	9510.0	49906.0	1190.2	(2)697.9	(3)892.8
HGSVCu	dtime	2885.9	—	(2)160.8	(1)139.7	1197.3	1175.7	333.2	(3)179.3	181.2	184.8
36 genomes	cmem	10.74	—	(2)2.30	3.55	(1)0.44	3.75	(3)2.64	26.31	23.41	33.00
102.88 GB	dmem	10.74	—	(2)2.15	(2)2.15	(1)0.21	3.94	(2)2.15	4.73	15.32	16.25
	ccpu	99	—	115	100	95	97	99	110	96	97
	dcpu	99	—	100	101	104	103	46	127	99	99
	ratio	12.1	39.6	4.2	23.1	4.8	35.4	(3)77.0	70.3	(2)105.0	(1)105.5
	ctime	24.3	465.1	(1)2.2	356.3	27.0	50.5	26.9	9.7	(2)2.5	(3)3.1
S. cerevisiae	dtime	8.4	1.1	(2)0.6	(1)0.4	6.4	3.2	(3)1.0	1.3	(3)1.0	1.1
39 genomes	cmem	2.48	4.99	(2)0.54	0.87	(1)0.37	3.35	0.97	(3)0.79	1.28	1.38
493.98 MB	dmem	2.48	0.50	0.50	0.50	(2)0.15	1.68	0.49	(1)0.05	(3)0.21	0.32
	ccpu	99	99	117	100	95	97	96	104	96	97
	dcpu	97	97	130	140	103	98	33	119	97	98
	ratio	67.58	63.39	35.38	65.43	(2)87.95	(1)114.98	(3)74.74	40.11	59.46	60.71
	ctime	62.17	324.25	(1)5.23	329.75	520.84	568.02	121.01	1182.14	(2)14.97	(3)17.47
Influenza	dtime	18.57	2.10	(2)0.69	(1)0.64	4.88	4.79	(3)1.09	5120.00	5.99	6.17
817,587 seq.	cmem	7.15	15.02	(2)1.57	2.60	(1)1.50	7.39	(3)1.81	3.81	2.31	2.54
1.43 GB	dmem	7.16	1.44	1.43	1.43	(1)0.16	2.21	1.43	16.04	(2)0.74	(3)0.97
	ccpu	99	99	116	100	99	99	99	100	98	99
	dcpu	99	97	188	192	111	108	99	100	99	99
	ratio	(2)885.41	553.40	354.28	530.61	259.59	419.56	519.14	(1)914.22	612.04	(3)617.28
	ctime	772.41	3630.00	(2)37.01	813.34	1386.90	1367.05	127.59	8599.00	(1)36.99	(3)39.82
COVID	dtime	198.79	18.78	(1)8.06	(2)8.20	83.09	97.19	(3)9.98	2095.59	15.48	15.76
620,304 seq.	cmem	10.75	40.81	(2)2.29	3.52	(1)1.07	4.67	(3)2.65	24.58	19.70	19.80
18.83GB	dmem	10.75	4.30	2.15	2.15	(1)0.18	3.86	2.24	41.65	(2)0.44	(3)0.59
	ccpu	99	99	119	101	99	99	99	100	94	94
	dcpu	98	98	159	162	111	108	98	100	99	99

The best three results are marked with a number in parentheses.
7z failed to compress HGSVCu dataset within the 100,000 seconds limit (denoted with "—").
[1]AGC options adjusted for Influenza and COVID datasets are `-a -s3000 -b10000`, and `-a -s1500 -b500` for bacteria (mixed and S. enterica), otherwise AGC runs with its defaults.

Table 12: Compression results - 6 threads limit

		BSC -p -b2047	7z -md4g	zstd -3 –long=31	zstd -19 –long=31	Genozip default	Genozip -b best	NAF -19 –long=31	AGC best ratio[1]	MBGC2 default	MBGC2 max
	ratio	29.2	101.4	14.1	61.3	5.4	47.6	(3)214.6	172.0	(2)382.2	(1)456.7
bacteria	ctime	207.4	4676.0	(3)26.1	1600.5	237.5	285.3	298.6	87.5	(1)10.1	(2)22.6
mixed	dtime	50.5	13.1	10.4	(3)9.3	95.0	79.5	36.2	17.4	(1)4.6	(2)5.8
4 × 1024 gen.	cmem	64.40	122.58	(3)2.31	4.05	(1)2.01	22.36	2.66	3.72	2.90	(2)2.19
14.94 GB	dmem	64.42	15.09	2.15	2.15	(2)1.03	19.84	2.24	(1)0.36	(3)1.85	2.04
	ccpu	392	263	204	594	620	597	96	405	365	193
	dcpu	402	164	119	103	534	511	23	582	419	329
	ratio	45.4	339.9	27.5	191.7	5.4	65.9	2175.6	(3)3325.2	(2)7804.4	(1)7998.9
S. enterica	ctime	685.7	22830.0	(3)73.9	5361.0	895.5	914.9	271.0	198.7	(1)20.1	(2)32.4
cluster	dtime	164.8	49.9	45.2	42.2	296.0	160.6	161.8	(3)38.7	(1)19.9	(1)19.9
14,003 genomes	cmem	64.42	122.50	(2)2.32	4.01	(1)2.17	21.66	2.66	3.02	2.54	(3)2.50
67.12 GB	dmem	64.42	17.24	2.15	2.15	0.99	19.77	2.27	(1)0.24	(2)0.59	(2)0.59
	ccpu	533	226	202	598	624	634	82	384	273	178
	dcpu	515	152	104	90	486	570	22	581	289	280
	ratio	38.4	247.2	20.7	148.8	5.3	60.4	1024.3	(3)1314.5	(2)2229.0	(1)2252.7
S. enterica	ctime	102.7	2830.3	(3)5.2	449.1	60.1	77.9	25.2	14.6	(1)0.9	(2)2.6
cluster part	dtime	28.6	5.4	3.1	3.0	13.0	6.1	11.8	(3)2.7	(1)1.0	(2)1.1
1,024 genomes	cmem	24.35	47.07	2.31	4.00	(3)1.53	20.57	2.64	3.02	(1)1.14	(1)1.14
4.87 GB	dmem	21.54	4.89	2.15	2.15	0.96	16.07	2.16	(1)0.19	(2)0.48	(2)0.48
	ccpu	226	120	228	583	619	471	86	380	416	191
	dcpu	216	109	117	97	504	450	23	574	470	425
	ratio	4.4	5.0	3.3	4.1	4.5	4.7	5.2	(3)96.6	(2)101.7	(1)115.2
	ctime	1330.7	69605.0	(1)152.6	18983.0	1328.5	1981.8	49906.0	(3)241.2	(2)204.4	618.1
HGSVCu	dtime	553.8	282.1	159.5	139.5	290.6	237.0	333.2	(1)51.8	(2)85.2	(3)112.4
36 genomes	cmem	64.42	125.89	(2)2.32	4.15	(1)1.09	21.30	(3)2.64	24.20	41.42	38.56
102.88 GB	dmem	64.42	20.72	(2)2.15	(2)2.15	(1)0.97	19.35	(2)2.15	11.67	36.99	36.93
	ccpu	590	250	408	598	616	651	99	604	298	139
	dcpu	574	291	101	101	512	601	46	468	270	193
	ratio	12.1	39.6	4.2	23.0	4.8	35.4	(3)77.0	70.3	(2)101.1	(1)105.5
	ctime	11.0	389.2	(1)0.7	115.7	7.1	49.4	26.9	(3)2.3	(2)0.8	2.6
S. cerevisiae	dtime	3.2	1.1	0.6	(2)0.4	1.7	3.4	1.0	(1)0.3	(3)0.5	0.8
39 genomes	cmem	2.48	5.00	(1)0.55	1.36	1.02	3.44	(3)0.97	(2)0.64	1.89	1.40
493.98 MB	dmem	2.49	0.51	(3)0.50	(3)0.50	0.74	1.68	(2)0.49	(1)0.18	0.52	0.75
	ccpu	453	117	419	427	567	189	96	524	303	133
	dcpu	461	98	131	139	470	99	33	626	334	206
	ratio	67.58	63.39	35.38	65.43	(2)88.05	(1)114.98	(3)74.74	40.11	53.80	60.71
	ctime	29.89	239.93	(1)5.34	80.42	101.45	225.02	121.01	951.12	(2)6.35	(3)11.83
Influenza	dtime	7.99	2.12	(2)0.68	(1)0.63	1.51	2.42	(3)1.09	1164.39	3.82	5.00
817,587 seq.	cmem	7.16	15.02	(1)1.58	3.07	3.49	17.95	(2)1.81	4.08	(3)2.48	2.54
1.43 GB	dmem	7.16	1.46	1.43	1.43	(1)0.78	2.94	1.43	12.72	(3)1.08	(2)0.96
	ccpu	469	115	147	532	667	260	99	129	337	153
	dcpu	495	99	188	192	409	216	99	599	200	131
	ratio	(2)885.41	555.19	354.28	530.61	256.03	425.90	519.14	(1)914.22	592.77	(3)617.30
	ctime	201.46	1255.04	(3)36.91	194.23	268.39	281.21	127.59	8966.00	(1)14.41	(2)30.23
COVID	dtime	48.19	14.32	(2)7.97	(1)7.92	24.37	25.02	(3)9.98	397.35	13.49	14.13
620,304 seq.	cmem	64.40	122.46	(2)2.30	3.95	(1)1.88	22.67	(3)2.65	23.45	20.06	19.77
18.83GB	dmem	64.42	18.87	2.15	2.15	(3)0.93	20.52	2.24	41.58	(1)0.42	(2)0.56
	ccpu	441	251	137	561	663	637	99	101	275	124
	dcpu	431	150	160	166	446	496	98	586	122	116

The best three results are marked with a number in parentheses.

[1] AGC options adjusted for Influenza and COVID datasets are `-a -s3000 -b10000`, and `-a -s1500 -b500` for bacteria (mixed and S. enterica), otherwise AGC runs with its defaults.

Table 13: Compression results - no threads limit

		BSC -p -b512	7z -md512m	zstd -3 –long=31	zstd -19 –long=31	Genozip default	Genozip -b best	NAF -19 –long=31	AGC best ratio[1]	MBGC2 default	MBGC2 max
	ratio	18.8	61.3	14.1	61.4	6.3	47.6	(3)214.6	172.0	(2)378.1	(1)456.8
bacteria	ctime	116.4	1181.1	(3)28.6	810.8	88.2	151.7	298.6	73.2	(1)11.6	(2)22.4
mixed	dtime	28.5	10.7	10.2	(3)9.3	41.2	59.9	36.2	14.4	(1)4.9	(2)6.1
4 × 1024 gen.	cmem	74.71	75.44	(2)2.32	4.82	7.42	94.86	(3)2.66	4.77	3.30	(1)2.19
14.94 GB	dmem	74.81	15.19	2.15	2.15	4.22	49.72	2.24	(1)0.51	(2)1.90	(3)2.03
	ccpu	2514	1323	200	1367	2551	1441	96	587	410	206
	dcpu	1628	328	119	100	2342	1155	23	1023	423	330
	ratio	23.9	103.2	27.5	191.5	6.3	65.9	2175.6	(3)3325.2	(2)7804.9	(1)7990.6
S. enterica	ctime	509.9	5853.0	(3)84.9	2729.3	307.0	346.2	271.0	168.6	(1)19.9	(2)32.2
cluster	dtime	81.3	(3)44.1	74.7	50.4	112.7	82.2	161.8	48.6	(1)16.3	(1)16.3
14,003 genomes	cmem	75.28	75.37	(1)2.36	4.76	7.52	99.15	2.66	3.90	(3)2.59	(2)2.48
67.12 GB	dmem	75.27	15.21	2.15	2.15	4.27	62.92	2.27	(1)0.34	(2)0.58	(3)0.60
	ccpu	2616	1148	189	1388	2540	2267	82	477	298	259
	dcpu	2332	285	65	74	2040	2414	22	909	346	345
	ratio	21.7	87.3	20.6	148.1	6.1	60.4	1024.3	(3)1314.5	(2)2229.0	(1)2252.7
S. enterica	ctime	42.9	512.1	(3)6.4	238.7	20.7	47.0	25.2	12.2	(1)1.7	(2)2.8
cluster part	dtime	11.1	3.5	3.1	(3)3.0	4.6	4.7	11.8	3.4	(1)1.0	(2)1.1
1,024 genomes	cmem	24.35	48.83	(3)2.32	4.72	6.03	30.71	2.64	3.34	(2)1.38	(1)1.15
4.87 GB	dmem	24.20	4.93	2.15	2.15	4.10	16.22	2.16	(1)0.28	(2)0.48	(3)0.51
	ccpu	855	933	220	1276	2238	729	86	478	317	281
	dcpu	660	229	116	94	2005	614	23	967	507	473
	ratio	4.3	4.2	3.3	4.1	4.5	4.7	5.2	(3)96.6	(2)101.4	(1)115.2
	ctime	759.2	12829.0	(1)180.7	9408.0	502.6	645.5	49906.0	(2)194.0	(3)205.4	631.4
HGSVCu	dtime	205.4	118.3	154.9	157.3	134.7	175.8	333.2	(1)52.9	(2)82.1	(3)106.2
36 genomes	cmem	75.27	77.34	(1)2.35	(3)5.08	5.40	101.31	(2)2.64	24.14	46.41	38.48
102.88 GB	dmem	75.27	18.65	(1)2.15	(1)2.15	4.37	67.33	(1)2.15	16.53	37.00	36.94
	ccpu	2680	1443	361	1390	1906	2396	99	799	314	153
	dcpu	2548	1044	103	91	1439	1020	46	612	269	202
	ratio	12.1	39.6	4.2	23.5	5.0	35.4	(3)77.0	70.3	(2)101.5	(1)105.5
	ctime	11.0	389.2	(1)0.9	76.6	4.0	49.4	26.9	(3)1.5	(1)0.9	2.7
S. cerevisiae	dtime	2.7	1.1	0.6	(2)0.4	1.1	3.6	1.0	(1)0.3	(3)0.5	0.8
39 genomes	cmem	2.49	5.00	(1)0.56	1.55	3.85	3.43	(3)0.97	(2)0.59	1.84	1.40
493.98 MB	dmem	2.49	0.51	(3)0.50	(3)0.50	1.89	1.68	(2)0.49	(1)0.33	0.53	0.75
	ccpu	1750	117	356	687	1057	189	96	858	399	165
	dcpu	1167	98	129	135	818	98	33	737	440	262
	ratio	60.87	62.39	35.38	65.43	(2)92.94	(1)114.98	(3)74.74	40.11	53.69	60.71
	ctime	25.19	106.20	(1)6.39	52.23	50.68	224.29	121.01	957.96	(2)6.48	(3)11.78
Influenza	dtime	6.16	1.43	(2)0.69	(1)0.63	1.36	2.43	(3)1.09	858.58	3.83	5.02
817,587 seq.	cmem	7.16	14.52	(1)1.59	3.79	17.63	17.93	(2)1.81	4.48	(3)2.48	2.55
1.43 GB	dmem	7.17	1.46	(3)1.43	(3)1.43	3.03	2.97	(3)1.43	4.84	(2)1.08	(1)0.96
	ccpu	260	284	142	896	2010	260	99	132	375	162
	dcpu	273	154	186	191	716	217	99	1395	208	136
	ratio	(3)593.77	534.31	354.28	530.61	297.15	410.71	519.14	(1)914.22	590.52	(2)617.29
	ctime	128.68	283.29	(3)42.35	145.21	109.43	143.18	127.59	8434.00	(1)13.24	(2)29.18
COVID	dtime	23.63	13.12	(2)7.92	(1)7.69	10.13	16.55	(3)9.98	186.57	13.62	14.15
620,304 seq.	cmem	75.17	75.28	(1)2.31	(3)4.65	7.22	105.87	(2)2.65	22.15	20.04	19.80
18.83GB	dmem	75.29	15.07	(3)2.15	(3)2.15	3.77	62.26	2.24	41.57	(1)0.42	(2)0.56
	ccpu	2209	1249	134	798	2708	1890	99	101	321	134
	dcpu	2062	282	158	168	1160	1309	98	1316	124	118

The best three results are marked with a number in parentheses.

[1]AGC options adjusted for Influenza and COVID datasets are `-a -s3000 -b10000`, and `-a -s1500 -b500` for bacteria (mixed and S. enterica), otherwise AGC runs with its defaults.
