# Supplementary material for: MBGC2: Boosting compression via efficient encoding of approximate matches in genome collections
Source: Gigascience. 2026 Jan 21;15:giag008. doi: 10.1093/gigascience/giag008 (PMC12934354; doi:10.1093/gigascience/giag008)
Supplement: giag008_GIGA-D-25-00291_Original_Submission [file giag008_giga-d-25-00291_original_submission.pdf]

GigaScience

MBGC2: Boosting compression via efficient encoding of approximate matches in genome collections

--Manuscript Draft--

**Manuscript Number:**	GIGA-D-25-00291
**Full Title:**	MBGC2: Boosting compression via efficient encoding of approximate matches in genome collections
**Article Type:**	Technical Note
**Funding Information:**	Politechnika Lódzka (501/12-24-1-5418) — Mr. Tomasz Marek Kowalski
**Abstract:**	Background: FASTA is the primary format for representing DNA, RNA and protein sequences. While progress has been made in specialized FASTA collection compressors, they still struggle with practical limitations and inconsistent performance across different datasets, hindering effective storage and transfer of large genomic datasets.<br>Results: We present an enhanced version of the Multiple Bacteria Genome Compressor (MBGC), a high-throughput, in-memory algorithm for compressing genome collections.<br>It relies on information about maximum exact matches in the compressed set to identify possibly long approximate matches. It encodes them even when they partially overlap, boosting the compression ratio by an average of 14\% across bacterial datasets, while the reengineered multi-threaded decompression speeds up decompression compared to its predecessor by 39\%. The compression ratio improvement is even more pronounced on other collections, for H.\ Sapiens reaching 18\%, and up to 56\% for S.\ paradoxus.<br>MBGC2 performs consistently across diverse datasets and introduces practical features to ease data management such as archive appending, repacking, fast content listing and flexible decompression options. Benchmark tests on bacterial, viral, and human genome collections show that MBGC2 combines compression efficiency and processing speed.<br>Conclusions: MBGC2 addresses critical limitations in genome collection compression by delivering reliable performance, improved compression ratios, and enhanced usability features. The consistent efficiency across diverse genomic datasets makes it a versatile tool for managing the growing volume of genomic data in research and clinical settings.<br>The balance between compression ratio and speed positions MBGC2 as a practical solution for the storage and transfer of large genomic collections.
**Corresponding Author:**	Tomasz Marek Kowalski, Ph.D<br>Lodz University of Technology: Politechnika Lodzka<br>Łódź, Łódź POLAND
**Corresponding Author Secondary Information:**	
**Corresponding Author's Institution:**	Lodz University of Technology: Politechnika Lodzka
**Corresponding Author's Secondary Institution:**	
**First Author:**	Tomasz Marek Kowalski, Ph.D
**First Author Secondary Information:**	
**Order of Authors:**	Tomasz Marek Kowalski, Ph.D
**Order of Authors Secondary Information:**	
**Additional Information:**	
**Question**	**Response**
Are you submitting this manuscript to a	No

Powered by Editorial Manager® and ProduXion Manager® from Aries Systems Corporation

special series or article collection?	
**Experimental design and statistics**<br><br>Full details of the experimental design and statistical methods used should be given in the Methods section, as detailed in our Minimum Standards Reporting Checklist. Information essential to interpreting the data presented should be made available in the figure legends.<br><br>Have you included all the information requested in your manuscript?	Yes
**Resources**<br><br>A description of all resources used, including antibodies, cell lines, animals and software tools, with enough information to allow them to be uniquely identified, should be included in the Methods section. Authors are strongly encouraged to cite Research Resource Identifiers (RRIDs) for antibodies, model organisms and tools, where possible.<br><br>Have you included the information requested as detailed in our Minimum Standards Reporting Checklist?	Yes
**Availability of data and materials**<br><br>All datasets and code on which the conclusions of the paper rely must be either included in your submission or deposited in publicly available repositories (where available and ethically appropriate), referencing such data using a unique identifier in the references and in the "Availability of Data and Materials" section of your manuscript.<br><br>Have you have met the above requirement as detailed in our Minimum Standards Reporting Checklist?	Yes

Powered by Editorial Manager® and ProduXion Manager® from Aries Systems Corporation

GigaScience has policies and guidelines in place for the use of generative AI-writing tools such as ChatGPT. If you have used such writing tools to assist with writing the manuscript this must be declared and cited in the text. Authors should not list AI-writing tools and other AI-assisted technologies as an author or co-author and should acknowledge that they are fully responsible for text generated or refined by AI-writing tools.<p><br><br>A summary of use (particularly in the introduction or among methods) needs to be included at the end of the paper, and the outputs should also be included as a supplementary file hosted in GigaDB or other open repositories. Please read our guidelines for more information. <p><br><br>By submitting to GigaScience, you are aware of the journal's AI-writing tools policy, and if you have declared use of such tools below, you have acknowledged this where appropriate in your manuscript and have made a summary of use and outputs available. <p><br><b>AI-assisted writing tools have been used in the preparation of this manuscript?	No

Manuscript

Click here to access/download;Manuscript;2025_MBGC2_GigaScience.pdf

GigaScience, 2025, 1–10

doi: xx.xxxx/xxxx
Manuscript in Preparation
Technical Note

TECHNICAL NOTE

MBGC2: Boosting compression via efficient encoding of approximate matches in genome collections

Tomasz M. Kowalski[1,*]

[1]Institute of Applied Computer Science, Lodz University of Technology, Poland

[*]tomasz.kowalski@p.lodz.pl

Abstract

Background: FASTA is the primary format for representing DNA, RNA and protein sequences. While progress has been made in specialized FASTA collection compressors, they still struggle with practical limitations and inconsistent performance across different datasets, hindering effective storage and transfer of large genomic datasets.
Results: We present an enhanced version of the Multiple Bacteria Genome Compressor (MBGC), a high-throughput, in-memory algorithm for compressing genome collections. It relies on information about maximum exact matches in the compressed set to identify possibly long approximate matches. It encodes them even when they partially overlap, boosting the compression ratio by an average of 14% across bacterial datasets, while the reengineered multi-threaded decompression speeds up decompression compared to its predecessor by 39%. The compression ratio improvement is even more pronounced on other collections, for H. Sapiens reaching 18%, and up to 56% for S. paradoxus. MBGC2 performs consistently across diverse datasets and introduces practical features to ease data management such as archive appending, repacking, fast content listing and flexible decompression options. Benchmark tests on bacterial, viral, and human genome collections show that MBGC2 combines compression efficiency and processing speed.
Conclusions: MBGC2 addresses critical limitations in genome collection compression by delivering reliable performance, improved compression ratios, and enhanced usability features. The consistent efficiency across diverse genomic datasets makes it a versatile tool for managing the growing volume of genomic data in research and clinical settings. The balance between compression ratio and speed positions MBGC2 as a practical solution for the storage and transfer of large genomic collections.

Key words: software, data compression, multiple genome compression, FASTA

Introduction

The amount of genomic data is increasing at an exponential rate [1]. While tools for the bioinformatics domain are being rapidly developed, repositories still seem to be dominated by gzip format and tar archives for representing sets of files. Such an approach usually fails to exceed the 4-fold compression for DNA stream [2, 3]. Efficient and reliable compression tools could reduce storage requirements and speed up the transfer, analysis and processing of large collections of data.

Genomic information is a challenging subject for the development of compression methods. DNA sequences tend to be highly redundant, but contain many single-nucleotide variations. They also contain various structural rearrangements (e.g., inversions, translocations, fusions, and inverted repeats) [2, 4, 5]. Many other phenomena that occur can be mentioned, such as copy number variation and clusters of structural variants that are formed by cut-and-paste and copy-and-paste mechanisms [5, 6]. In addition, various factors (e.g., exogenous DNA contamination and environmental causes) add additional heterogeneity or artifacts to sequencing reads, affecting sequence quality and downstream analyses. Effective modeling and compression require techniques that are capable of handling such dynamic and imperfect genomic data [7, 8].

Modern genomic data compressors usually target specific data formats. Most often, these are FASTA files oriented to store complete or partial sequences from one or many individuals or strains, and FASTQ files, which have become the standard for storing output from high-throughput sequencing instruments. Compressors can

Compiled on: July 21, 2025.
Draft manuscript prepared by the author.

Key Points

- High compression performance across diverse genomic datasets from bacterial collections to human genomes
- Enhanced usability with flexible decompression options and archive, appending, repacking, and fast content listing capabilities.
- Compression ratio improved by 14% over MBGC1 on bacteria via carefully engineered approximate match encoding
- Multi-threaded decompression delivered 39% speed improvement over MBGC1 while maintaining memory efficiency

Table 1. Comparison of FASTA compressor features (the ✓ symbol indicates a supported function, — otherwise).

	NAF	Geno-zip	GDC 2	HRCM	AGC	MBGC 1 / 2	general-purpose tools (7z, zstd, bsc, pigz)
handles collection of genomes natively[1]	—	—	✓	✓	✓	✓ / ✓	— (except 7z)
compresses without explicit reference	✓	✓	—	—	—	✓ / ✓	✓
decompresses without reference	✓	✓	✓	—	✓	✓ / ✓	✓
preserves sequences line length[2]	✓	✓	✓	✓	—	— / ✓	✓
preserves bases letter case	✓	✓	✓	✓	—	✓ / ✓	✓
preserves original path structure	—	—	✓	—	—	✓ / ✓	— (except 7z)
handles single multi-FASTA file	✓	✓	—	—	✓	✓ / ✓	✓
decompresses selected FASTA files	—	—	✓	—	✓	— / ✓	— (except 7z)
random access to individual FASTA	—	—	—	—	✓	— / —	✓ (if not lumped[1])
can append genomes to archive	—	—	—	—	✓	— / ✓	— (except 7z)
compresses gzipped FASTA	—	✓	—	—	✓	✓ / ✓	—

[1]Some tools (i.e., NAF, BSC, pigz) require lumping genomes together to compress them into a single archive, e.g., using tar or mumu.pl script (available at https://github.com/KirillKryukov/mumu), or produce tar of multiple archives (Genozip).
[2]Some specialized genome compressors correctly preserve line length format in sequences only if it is consistent throughout a whole file (e.g., in case of NAF, GDC 2, MBGC2) or an individual sequence (e.g., HRCM).

be broadly divided into two groups depending on whether they use an external reference sequence for compression and decompression. Both approaches were already implemented in Biocompress, the first specialized genome compressor [9]. So-called reference-based (vertical) methods leverage the similarities in a compressed nucleotide stream to a selected reference, encoding only the differences to achieve high compression. Examples include tools targeting FASTQ datasets, such as LW-FQZip [10], and RBFQC [11] and tools for FASTA, such as DNAzip [12], GenomeZip [13], IDoComp [14], NRGC [15], RSS [16], HRCM [17], memRGC [18], LMSRGC [19], and many more [20]. Reference-free (horizontal) methods completely remove the requirement for an external reference sequence by exploiting intra-sequence redundancies (e.g., palindromes and statistical properties) and across sequences in the dataset. These tools commonly employ statistical or dictionary-based models, bit encoding, context modeling, run-length encoding, and Huffman coding. Notable examples are XM [21], MFCompress [22], Delimi-nate [23], DSRC2 [24], and Leon [25]. A recent trend in reference-free compression is the integration of machine learning models, such as neural networks, to further improve compression ratios. GeCo2 [26], GeCo3 [27], Jarvis3 [28], or HMG [29] exemplify this direction. Most reference-free tools are designed for standalone nucleotide sequences or assembled genomes in FASTA format (of the above, the exception is DSRC2 for FASTQ files). However, several tools are also capable of compressing sequencing data with quality streams in FASTQ, e.g., BEETL [30, 31], FQZComp [32], SPRING [33], NAF [34], and Jarvis3 [28]. Some compressors, such as Mstcom [35] and PgRC [36, 37], focus on compressing only the DNA sequence stream from FASTQ files, offering high efficiency for applications where quality scores are not required. It is worth noting that some implementations leverage hardware acceleration, such as CUDA-based solutions, to further speed up the compression of FASTQ data [38, 39].

Beyond compression, efficient indexing and search in genomic collections are essential for practical applications. Compressed indexes such as MuGI [40], JST [41], PgSA [42], BIGSI [43], and Block Trees [44] have expanded the capabilities for fast pattern queries, scalable indexing, and efficient analysis across large-scale genomic databases. The development of such tools is closely linked to the ideas behind advanced compressors, as both fields benefit from exploiting redundancy and structure in genomic data. Other solutions, like copMEM [45, 46] and bfMEM [47], provide robust and scalable maximal exact match (MEM) finding, which is crucial for comparative genomics and pan-genomic analyses. These methods also contribute to the implementation of efficient compression [36, 18]. A major recent development is the design of robust strategies for large-scale microbial genome searches. The approach by Břinda et al. [48] shows that taking advantage of phylogenetic relationships to reorder and compress data can enable efficient and robust search across millions of microbial genomes. Their pipeline allows BLAST-like queries against all sequenced bacteria using ordinary computer hardware, providing solutions that were previously unfeasible, e.g., for aligning genes, plasmids or entire sequencing experiments. The AllTheBacteria initiative [49] is pursuing the above strategy. It employs phylogenetic compression in collecting and curating over 2.4 M publicly assembled bacterial genomes into a searchable, and accessible resource. Further, beyond storage and search, compression-based features have found direct application in downstream analysis. Studies have shown that compression-derived metrics, such as Normalized Compression (NC) or Normalized Compression Distance (NCD), can be utilized for taxonomic classification and metagenomic analysis [50, 51]. Quantifying the similarity of sequences without explicit alignment provides alternative approach for clustering, classification, and detection of evolutionary relationships among complex microbial communities. In summary, the synergy between indexing and analytics and compression underlie many scalable and effective strategies for managing, querying, and interpreting genomic data at population and metagenomic scales [52, 53, 54].

In this work, we focus on tools developed specifically for compressing genomic collections in FASTA format. When compression addresses multiple genomes, a compressor can integrate and

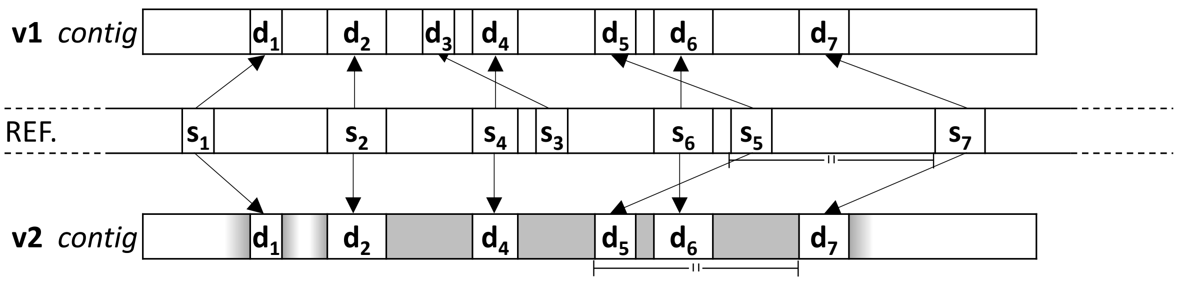

Figure 1. Toy example of contig encoding in MBGC1 and MBGC2. Regions encoded using *encoding in gaps* technique are highlighted using solid gray color (e.g., a region between 2nd and 4th match) . A gray gradient in the spaces between matches (e.g., around d_1) indicates the span of *adjacent encoding*.

advance ideas from both reference-based and reference-free approaches. Early solutions for compressing collections of genomes adapted LZ77 algorithm to leverage redundancy across multiple genomes. To this end, RLZ [55] relied on a single selected reference sequence. Subsequent tools either explored strategies to expand the set of reference sequences (e.g., GDC and its successor [56, 57]) or used second-order compression on already reference-compressed files (e.g., FRESCO [58]). Nevertheless, these tools assumed that genome collections are given as sets of complete chromosomes and processed the data chromosome by chromosome. In contrast, contemporary de novo assemblies typically yield sets of contigs (so called multi-FASTA format) of varying lengths, with unknown chromosomal origins for each contig. This shift increases the complexity of the compression problem.

While numerous tools, such as FASTA or general purpose compressors, can be utilized for compressing collections of assembled nucleotide sequences, they suffer from performance variability across different datasets. Their individual design and purpose make each solution suffer from certain drawbacks or limitations that impinge on their usability and versatility (cf. Table 1). Good result in compression of multi-FASTA collections can be achieved by relatively simple specialized tools. For instance, NAF [34] strips End-Of-Line (EOL) symbols before packing two nucleotides into a single byte and performs zstd compression on the backend. By enabling long distance matching in zstd, NAF can boost the ratio in the case of highly repetitive data [59]. The most recent advance in genomic collections compression is AGC [60]. Its method involves identifying so-called splitters, i.e., unique k-mers evenly distributed in the reference, which divide sequences into segments. Then, corresponding segments are encoded using the LZSS algorithm [61] and the zstd compressor. AGC is exceptional because it offers high compression performance along with advanced features such as fast access to any contig and archive updates.

Here, we present MBGC2, the successor to the MBGC tool [59], a novel compressor designed to provide consistent, high-performance compression of genome collections. It improves the reliability of its predecessor by fixing critical errors reported in [60] concerning decompression of huge bacterial collections (i.e., Blackwell dataset of ~661 K assemblies [62]) and compression of collections with large FASTA files (e.g., containing whole H. sapiens genomes). Achieving better compression ratios is made possible by carefully designed and engineered efficient approximate matches encoding, which was inspired by previous works [18, 20, 39]. Finally, MBGC2 uses multi-threaded decoding for significant speed gains, and introduces practical features such as archive listing, appending, repacking and flexible decompression options.

Methods overview and Implementation

MBGC2 and its predecessor are built upon the concept of relative compression, leveraging redundancy within genome collections. Similar to approaches employed in previous works [56, 63], in MBGC we utilized a reference buffer to capture shared patterns among the genomes. Specifically, MBGC uses the first genome in the collection as the initial reference. Subsequently, it identifies and stores both direct and reverse-complemented MEMs between the reference and each genome within the collection. The consideration of reverse complements is particularly suited to bacterial genomes. To further enhance compression efficiency, MBGC dynamically extends the buffer. Sequences that exhibit limited similarity to the existing reference, as determined by the exact matching criteria, are incorporated into the reference, effectively expanding the reference base and boosting the overall compression ratio. This dynamic approach, similar to techniques aimed at improving the quality of the reference [63], ensures that the buffer adequately captures the diversity within the genome collection and leads to more effective compression.

Unlike the original MBGC, which focused solely on MEMs, MBGC2 allows for a limited number of mismatches surrounding an identified MEM. This flexibility significantly improves the ability to capture similar regions even in the presence of minor variations between genomes. The contig coding example shown in Fig. 1 illustrates the evolution from MBGC1 to MBGC2. The subsequences $d_1, d_2, \ldots, d_7$ in a contig represent MEMs in the reference denoted as $s_1, s_2, \ldots, s_7$. Distances between s_2, s_4, and s_6 are equal to ones between d_2, d_4, and d_6, respectively. This applies also to matches with indexes 5 and 7. We denote all such groups of matches as *corresponding matches*, and non-empty regions between them are referred to as *gaps*. Note that, as in the example figure, groups of corresponding matches often overlap. In MBGC1, matches in the contig are replaced by a MATCH_MARK symbol (%), and the resulting sequence is appended to the literals stream. Information about the matches, i.e., lengths and beginning positions of $s_1, s_2, \ldots, s_7$ were sent to separate streams. In MBGC version 2, there are two additional streams for more efficient encoding of matches and their adjacent surroundings. The final result is obtained by extending the original encoding schema with the following techniques, which allow individual matches or sets of corresponding matches to be treated more broadly as an approximate match.

(a) Encoding in gaps – Bases within a gap are encoded relatively to the corresponding region in the reference. If corresponding bases are not equal (mismatch), a set flag is stored in the mismatches

MISMATCH →; rows: ACTUAL REFERENCE

	**A**	**C**	**G**	**T**	**N**
**A**	-	2	0	1	3
**C**	1	-	2	0	3
**G**	0	2	-	1	3
**T**	1	0	2	-	3
**N**	1	2	3	0	-

Figure 2. Mismatch encoding matrix.

flags stream, and the mismatch is encoded in the literals stream. Mismatched bases are mapped by *exclusive mismatch encoding matrix* (cf. Fig. 2) into values 0, 1, 2, and 3 and sent to the literals stream. This procedure aims to encode more likely point mutations as lower values. We experimented with a dynamic contextual approach (along the lines of the approach implemented in CURC [39]), but adjusting the matrix to the each genome did not yield a visible improvement in compression ratio and significantly complicated the compression and decompression process. Finally, we chose a static matrix favoring G↔A and C↔T transitions as well as higher overall AT content. Encoding using an exclusive mismatch matrix improves compression ratio by up to 3.6 % (cf. Table 2).

(b) Adjacent encoding – If a direct neighborhood of match d_i lies outside of a gap, a portion of the bases to the left and right of the match is encoded relatively to the neighborhood of s_i in the reference. The encoding is stopped if relative frequency of mismatches exceeds a certain level. By introducing the *adjacent encoding* technique, we attempt to encode the sequence characters surrounding the MEM relatively to the corresponding characters in the reference. Bases adjacent to the MEM are encoded using the same two streams as in the case of encoding in gaps. Since the similarity between the sequence and the reference can deteriorate unpredictably, we decided to use a *mismatch scoring routine* to terminate the encoding of adjacent mismatches. The following parameters determine the behavior of this routine:

- Y = 25% - initial score relative to the terminating threshold (i.e., 100%),
- x = 10 - maximum number of consecutive mismatches causing termination of encoding, which translates into 10% mismatch penalty as well as an equal bonus (for each matching base).

The scoring strategy with the above settings allows for longer approximate matches than the one proposed in memRGC [18], which was tailored to the genomes of *H. sapiens*. MEM expansion was terminated if the number of consecutive mismatching (resp. matching) bases was greater (resp. less) than 2 (resp. 3). The MBGC2 procedure for encoding the area adjacent to a MEM is explained on the toy example in Fig. 3 (note the non-standard value of the x parameter). 8 bases of contig starting from 11th position matched REF at position 21. Encoding mismatches routine starts from left side of a match. As base G directly preceding the match is known to be a mismatch (otherwise it would be incorporated into the MEM), there is no need for a mismatch flag, and the base is encoded relatively to base T (20th REF position) according to exclusive mismatch encoding matrix into value 2 and pushed to literals stream. At this point the initial matching score (25%) is set. The following bases (in the reversed order) are processed sequentially. If a contig base matches a corresponding REF base (e.g. 9th and 6th contig positions), 0 flag is sent to mismatches flags stream, and mismatches score diminishes (25% bonus). In case of a mismatch (e.g., 8th and 7th contig positions), mismatch flag is set, literals are extended with exclusively encoded mismatch value, and score is increased (25% penalty). Reaching 100% mismatches score (at position 3 in a contig) terminates encoding routine. Literals are appended with remaining bases AT (in the standard direction) and a match mark. Encoding of right side of a match is done analogous but in the standard direction. Notice, that the score cannot drop below 0 (contig position 20th and following). Finally, the remaining bases TCG and sequence mark are appended to literals stream. Tests have shown that completely abandoning the improved coding of bases within gaps and adjacent to a match leads to a ~10% average loss in ratio (cf. Table 2).

Table 2. Boosting compression ratio with the implemented novel techniques. Ratio relative to MBGC2 max mode (-m3) in percents (the ✓ symbol indicates an enabled technique, — otherwise).

options	MBGC v2.0 -m3 -b	-X	-g0	-G0x0	v1.2.2 -c3
gaps delta encoding	✓	✓	—	✓	—
gap breaks filter	—	✓	—	—	—
encoding in gaps	✓	✓	—	—	—
adjacent encoding	✓	✓	✓	—	—
mismatch exclusion	✓	—	✓	—	—
C. jejuni	-7.9	-2.1	-1.1	-14.4	-10.3
S. enterica	-5.6	-1.1	-1.6	-7.7	-3.3
168,311 bacteria	-5.8	-1.7	-1.3	-11.3	-9.4
C. jejuni 1024	-3.6	-3.5	-7.6	-20.5	-25.0
S. en. cluster	-0.3	-0.9	-2.9	-3.4	-4.0
S. cerevisiae	-1.1	-1.0	-8.6	-5.9	-13.7
S. paradoxus	-2.0	-2.3	-11.4	-19.9	-26.6
HGSVCu	-1.0	-1.7	-4.3	-4.1	-12.2
HPRC	-2.3	-2.1	-5.3	-5.9	-22.8

(c) Gap breaks filter – If matches adjacent to a short-enough match (<256 by default) are corresponding matches (e.g., d_3 between corresponding matches d_2 and d_4) then the match is ignored, and its bases are encoded with the encoding in gaps technique. Encoding bases in gaps in MBGC2 is now cheaper. We have observed that if a potential long gap is interrupted by a MEM that refers to a different reference region, it is sometimes beneficial to ignore it and rely on exclusive encoding of mismatches within the region of the match. Experimentally, we have determined that such MEMs should be discarded if they consist of less than ~256 bases. This operation yields ~6% compression ratio improvement for large collections of bacteria (cf. Table 2).

(d) Gaps delta encoding – Not every beginning position of s_i needs to be stored explicitly. An additional stream is used to indicate positions that can be determined on the basis of the corresponding matches relationship (i.e., s_4, s_6 and s_7). For this purpose, in MBGC2, we added *gaps delta* stream to store the number of *gaps* between the current match and its closest subsequent corresponding match, where a gap is a non-empty region between adjacent MEMs. This number is reduced by ignoring matches corresponding to other matches preceding the current match. MBGC2 detects corresponding matches within a contig if there are less than 64 other matches between them. If there is no corresponding match to the current match, a value of 0 is sent to the gaps delta stream. The last match cannot have a corresponding match following it, thus it is ignored. On average, encoding matches offsets with the help of gaps delta stream yields ~5% overall ratio improvement. Let us consider gaps delta encoding using the example given in Fig. 1). Beginning positions of matches s_1, s_2, s_4, s_5, s_6, s_7 (match with index 3 was rejected by gap breaks filter) are encoded now using two streams. The match offsets stream stores the positions of matches if they are not corresponding to the matches that precede them (i.e., s_1, s_2, and s_5) and the gap delta stream holds the values 0, 1, 2, 1, 0. The value for the 4th match is 2 because its corresponding 6th match is after 5th one. On the other, for seemingly similar situation for the 5th match we obtain gap delta 1, because s_6 is not stored in the first stream. Such encoding allows for determining the beginning positions of the s_4, s_6 and s_7 matches during decoding.

See Supplementary Materials for more details on adjacent encoding, the matching scoring routine (cf. Fig. 4), as well as considerations on encoding in gaps.

Finally, the resulting data streams representing approximate matches (offsets, lengths, gaps delta, mismatch flags), literals, header and filename data are compressed with LZMA and PPMd algorithms. The combination of these new techniques implemented in MBGC2 yields a substantial reduction in the overall size of genome collections (cf. Table 2).

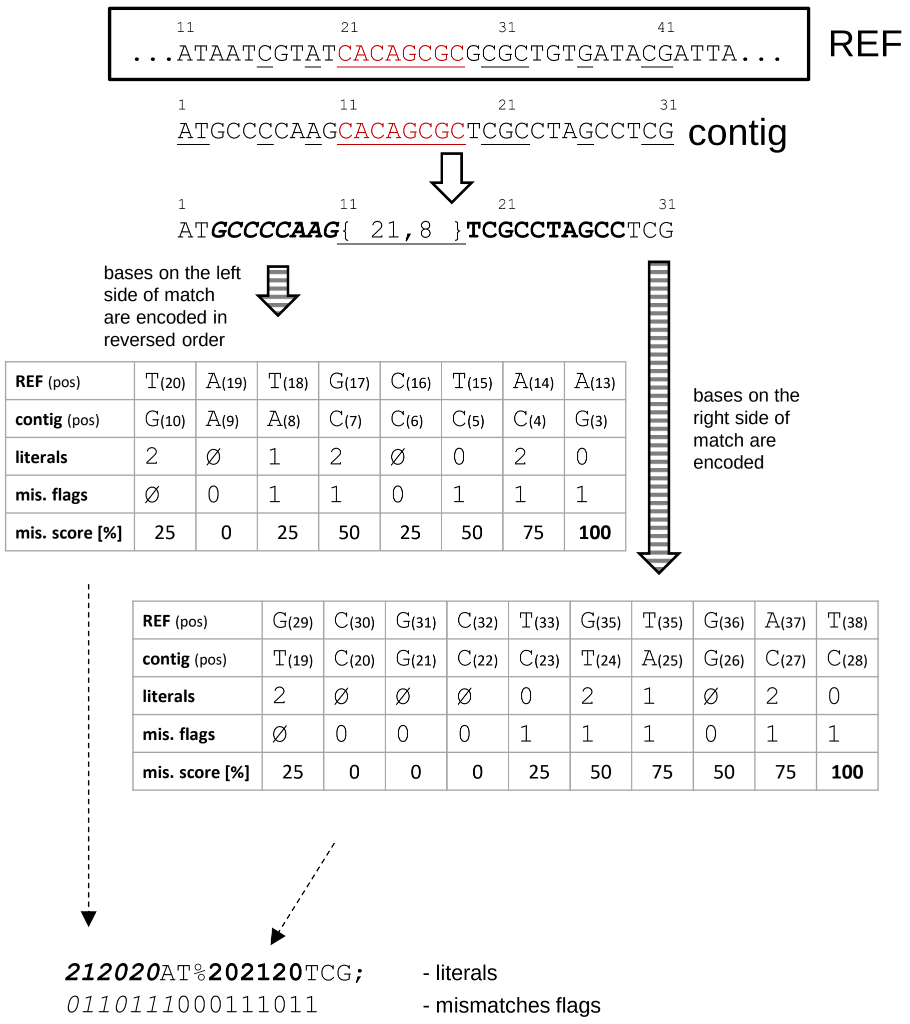

Figure 3. Toy example of encoding mismatches adjacent to a match. The maximum number of consecutive mismatches for the mismatch scoring routine is set to $x = 4$.

Results

The benchmark was run on a Linux workstation equipped with a 14-core Intel Core i9-10940X 3.3 GHz CPU, 128 GB of DDR4-RAM (CL 16, 2666 MHz) and an SSD (ADATA 4 TB M.2 PCIe Legend 960). The test collection consists of several real genome datasets, used in prior works on FASTA collection compression. MBGC1 uses 8 threads, NAF is single-threaded, and the rest of the tools use 28 threads. The detailed results of experiments on pathogens, *H. sapiens* genome collections, yeasts, and ribosomes are presented in Tables 3 – 6. The Supplementary Material provides more results (including proteins), details on datasets, and the test methodology. Compression ratios are given as a ratio of input to output sizes. Compression (resp. decompression) times, presented in rows or columns as "ctime" (resp. "dtime"), are given in seconds. Memory usage, "cmem" (resp. "dmem") for compression (resp. decompression) and dataset sizes are specified in GB, where G = 10^9. The best three results are marked with a number in parentheses. The pathogen datasets are mostly the same as in our previous work [59], while the set of competitors now comprises zstd, Genozip, NAF, AGC, and the previous public version of MBGC, v1.2. Genozip [64] is a popular commercial compressor in the bioinformatics community. It targets various formats, however the authors do not describe the details of reference-free FASTA file compression method.

The results confirm the dominance of MBGC variants on bacterial collections (cf. Table 3). The next best tool in terms of compression ratio is usually AGC, which, however, requires adjusting its internal parameters. As compared to the default mode of MBGC2, it is about 2 times weaker in ratio and slower by up to 17.0 (resp. 6.5) times in compression (resp. decompression). Of the other tools, only NAF remains a viable choice. AGC and MBGC2 are the only tools that can take advantage of redundancy in *H. sapiens* genome collections (cf. Table 4) comprised of large (more than 3 GB) FASTA files. Note that MBGC1's default mode could not compress such large files due to a critical error. Both tools show high compression efficiency, but twice faster decompression and the almost twofold reduction in memory usage make AGC the preferred option when using mid-range workstations or laptops with limited memory resources. In the experiment with two yeast genome collections (Table 5) we added results for GDC tool. It couldn't be executed on other datasets as it assumes that the genomes are given as sets of chromosomes. MBGC2 and GDC 2 are quite fast and superior in the compression ratio. The former in the slowest max mode is faster in compression by a factor of 1.4–7.6 while the latter needs 28 % less time to decompress for the *S. cerevisiae* dataset. AGC offers a slightly worse ratio (as well as NAF) but has a noticeable lead in decompression speed. In RNA compression ratio (cf. Table 6), MBGC2 is second only to the much slower Genozip variant by up to 15 %, with compression speeds only slightly weaker than the fast zstd and NAF variants. Unsatisfactory, however, is the inferior decompression performance, being up to ~6 times than zstd.

One way to evaluate compressor performance is by looking at the worst-case scenarios and comparing its results with the best in the category. The strongest indicator of MBGC2 is the compression ratio. The archive size for a collection of *Influenza* virus sequences (cf. Table 3) is just over 2 twice as large (2.15 to be exact) compared to the best competitor (namely Genozip), which, however, required >30 times longer to compress. The next best tool that offers attractive compression levels (within an order of magnitude) is AGC. Specifically, with adaptive mode (`-a` option designed for highly divergent species) enabled and settings suggested by the authors for the COVID virus collection, in the worst-case scenario (i.e., also with *Influenza* dataset) the compression ratio is 2.9 times worse against Genozip. Yet, this is paid for with as much as three orders of magnitude slower decompression compared to zstd. Tuning the AGC for decompression speed degrades either the compression speed (by up to three orders of magnitude) or the compression ratio (sometimes by more than one order of magnitude). In terms of speed, MBGC2 performs worst with protein sequences (cf. Table 8 in Supplementary Materials). It is slower in compression (resp. decompression) by a factor of 35 (resp. 88) to its fastest competitor (i.e., zstd). Excluding the protein collections, decompression is at most 7.7 times slower than the competition (for a small *Mitochondrion* dataset). Some general-purpose compressors can be tuned to provide high and stable speed (in both compression and decompression) across all datasets. In particular, zstd used with compression level 3 and long-distance matching enabled (`-long=31` option) is at worst only 4.4 (resp. 4) times slower in compression (resp. decompression) than the best competitor (cf. 168,311 pathogens and *S. Enterica* cluster results in Table 3). However, the high speed comes at a price of sometimes above two orders of magnitude smaller ratio when compared to the most ratio-efficient tool.

In summary, the presented experiments (including additional ones conducted on various DNA collections) show that MBGC2 is a tool offering a Pareto-optimal solution, while being a strong practical compromise in terms of reliable speed and high compression ratio.

Conclusion

MBGC 2.0 is a significantly enhanced version of the highly efficient MBGC tool for compressing collections of FASTA files. Our tool achieves high compression ratios while maintaining reasonably high compression and decompression speeds. Unlike previous methods that may be sensitive to the composition or diversity of the input data, MBGC2 consistently delivers strong performance across a wide range of genome collections. It improves the compression ratio of its previous version (v1.2) by 14% (across bacterial datasets), achieved through incorporating an encoding scheme that efficiently captures approximate matches between genomic sequences. Furthermore, MBGC2 has been reengineered to use threads to decode different genomes, resulting in around 39% speedup. The release offers several new features such as more flexible decompression options, quick listing archive contents, repacking, and adding new files to already existing archives. It also fixes essential issues of MBGC1, such as those concerning the processing of extra-large files and collections. All of these enhancements make MBGC2 a highly efficient, reliable, and easy-to-use tool for the management, storage, and transfer of genomic data of any size and type.

Availability of source code and requirements

- Project name: MBGC: Multiple Bacteria Genome Compressor
- Project home page: https://github.com/kowallus/mbgc
- Operating system(s): Linux, MacOS, Windows
- Programming language: C++
- Other requirements: C++17 standard or higher, cmake 3.5 or higher
- License: e.g. GNU GPL v3.0
- biotools ID: mbgc
- RRID:SCR_021875

Declarations

List of abbreviations

AGC: Assembled Genomes Compressor; BIGSI: Bacterial and Viral Genomic Sequence Index; BLAST: Basic Local Alignment Search Tool; BSC: Block Sorting Compressor; CURC: CUDA-based Reference-Free Read Compressor; DSRC2: DNA Sequence Read Compressor 2; EOL: End-Of-Line (character); GCC: GNU Compiler Collection; GDC 2: Genome Differential Compressor 2; GeCo2: Genomic Compressor 2; GeCo3: Genomic Compressor 3; HGSVC: Human Genome Structural Variation Consortium; HPRC: Human Pangenome Reference Consortium; HRCM: Hybrid Referential Compression Method; JST: Journaled String Tree; LW-FQZip: Light-Weight Reference-Based Compression of FASTQ Data; MBGC: Multiple Bacteria Genome Compressor; MEM: Maximal Exact Match; MFCompress: Multi-FASTA Compressor; MuGI: Multi-Genome Index; NAF: Nucleotide Archival Format; NCBI: National Center for Biotechnology Information; NRGC: Novel Referential Genome Compression; LZ: Lempel-Ziv; LZMA: Lempel-Ziv-Markov chain-Algorithm; PPMd: Prediction by Partial Matching (variant by Dmitry Shkarin); PgRC: Pseudogenome-based Read Compressor; PgSA: Pseudogenome Suffix Array; RBFQC: Referential-Based FASTQ Compressor; TAR: Tape Archive (file format); zstd: Zstandard.

Ethical Approval

Not applicable.

Consent for publication

Not applicable.

Competing Interests

The authors declare that they have no competing interests.

Funding

This work was partially supported by the Faculty of Electrical, Electronic, Computer, and Control Engineering, Lodz University of Technology, as a statutory activity.

References

1. Stephens ZD, Lee SY, Faghri F, Campbell RH, Zhai C, Efron MJ, et al. Big data: astronomical or genomical? PLoS biology 2015;13(7):e1002195.
2. Hosseini M, Pratas D, Pinho AJ. A survey on data compression methods for biological sequences. Information 2016;7(4):56.
3. Kryukov K, et al. Sequence Compression Benchmark (SCB) database—A comprehensive evaluation of reference-free compressors for FASTA-formatted sequences. GigaScience 2020;9(7):1–12.
4. Roeder GS, Fink GR. DNA rearrangements associated with a transposable element in yeast. Cell 1980;21(1):239–249.
5. Li Y, Roberts ND, Wala JA, Shapira O, Schumacher SE, Kumar K,

et al. Patterns of somatic structural variation in human cancer genomes. Nature 2020;578(7793):112–121.
6. Bohlin J, Pettersson JHO. Compression rates of microbial genomes are associated with genome size and base composition. Genomics Informatics 2024;22(1):16.
7. Zajac GJ, Fritsche LG, Weinstock JS, Dagenais SL, Lyons RH, Brummett CM, et al. Estimation of DNA contamination and its sources in genotyped samples. Genetic epidemiology 2019;43(8):980–995.
8. Slooten K. The analogy between DNA kinship and DNA mixture evaluation, with applications for the interpretation of likelihood ratios produced by possibly imperfect models. Forensic Science International: Genetics 2021;52:102449.
9. Grumbach S, Tahi F. Compression of DNA sequences. In: Proc. Data Compression Conference, IEEE; 1993. p. 340–350.
10. Zhang Y, Li L, Yang Y, Yang X, He S, Zhu Z. Light-weight reference-based compression of FASTQ data. BMC bioinformatics 2015;16:1–8.
11. Kumar S, Singh MP, Nayak SR, Khan AU, Jain AK, Singh P, et al. A new efficient referential genome compression technique for FastQ files. Functional & Integrative Genomics 2023;23(4):333.
12. Christley S, et al. Human genomes as email attachments. Bioinformatics 2009;25(2):274–275.
13. Pavlichin DS, et al. The human genome contracts again. Bioinformatics 2013;29(17):2199–2202.
14. Ochoa I, et al. iDoComp: a compression scheme for assembled genomes. Bioinformatics 2015;31(3):626–633.
15. Saha S, Rajasekaran S. NRGC: a novel referential genome compression algorithm. Bioinformatics 2016;32(22):3405–3412.
16. Cheng KO, Law NF, Siu WC. Clustering-based compression for population DNA sequences. IEEE/ACM transactions on computational biology and bioinformatics 2017;16(1):208–221.
17. Yao H, et al. HRCM: An Efficient Hybrid Referential Compression Method for Genomic Big Data. BioMed Research International;2019:3108950.
18. Liu Y, et al. Allowing mutations in maximal matches boosts genome compression performance. Bioinformatics 2020;36(18):4675–4681.
19. Lu Z, Guo L, Chen J, Wang R. Reference-based genome compression using the longest matched substrings with parallelization consideration. BMC bioinformatics 2023;24(1):369.
20. Kredens KV, Martins JV, Dordal OB, Ferrandin M, Herai RH, Scalabrin EE, et al. Vertical lossless genomic data compression tools for assembled genomes: A systematic literature review. Plos one 2020;15(5):e0232942.
21. Cao MD, et al. A simple statistical algorithm for biological sequence compression. In: Proc. Data Compression Conference, IEEE; 2007. p. 43–52.
22. Pinho AJ, Pratas D. MFCompress: a compression tool for FASTA and multi-FASTA data. Bioinformatics 2014;30(1):117–118.
23. Mohammed MH, et al. DELIMINATE—a fast and efficient method for loss-less compression of genomic sequences: Sequence analysis. Bioinformatics 2012;28(19):2527–2529.
24. Roguski Ł, Deorowicz S. DSRC 2—Industry-oriented compression of FASTQ files. Bioinformatics 2014;30(15):2213–2215.
25. Benoit G, Lemaitre C, Lavenier D, Drezen E, Dayris T, Uricaru R, et al. Reference-free compression of high throughput sequencing data with a probabilistic de Bruijn graph. BMC bioinformatics 2015;16:1–14.
26. Pratas D, et al. GeCo2: An optimized tool for lossless compression and analysis of DNA sequences 2019;p. 137–145.
27. Silva M, et al. Efficient DNA sequence compression with neural networks. GigaScience 2020;9(11). Giaa119.
28. Sousa MJP, et al. JARVIS3: an efficient encoder for genomic data. Bioinformatics 2024;40(12):btae725.
29. Nawaz MZ, Nawaz MS, Fournier-Viger P, Nawaz S, Lin JCW, Tseng VS. Efficient genome sequence compression via the fusion of MDL-based heuristics. Information Fusion 2025;120:103083.
30. Cox AJ, Bauer MJ, Jakobi T, Rosone G. Large-scale compression of genomic sequence databases with the Burrows–Wheeler transform. Bioinformatics 2012;28(11):1415–1419.
31. Janin L, Rosone G, Cox AJ. Adaptive reference-free compression of sequence quality scores. Bioinformatics 2014;30(1):24–30.
32. Bonfield JK, Mahoney MV. Compression of FASTQ and SAM format sequencing data. PloS one 2013;8(3):e59190.
33. Chandak S, Tatwawadi K, Ochoa I, Hernaez M, Weissman T. SPRING: a next-generation compressor for FASTQ data. Bioinformatics 2019;35(15):2674–2676.
34. Kryukov K, et al. Nucleotide Archival Format (NAF) enables efficient lossless reference-free compression of DNA sequences. Bioinformatics 2019;35(19):3826–3828.
35. Liu Y, Li J. Hamming-shifting graph of genomic short reads: Efficient construction and its application for compression. PLoS Computational Biology 2021;17(7):e1009229.
36. Kowalski TM, Grabowski S. PgRC: pseudogenome-based read compressor. Bioinformatics 2020;36(7):2082–2089.
37. Kowalski TM, Grabowski S. PgRC2: engineering the compression of sequencing reads. Bioinformatics 2025;41(3):btaf101.
38. Amich M, De Luca P, Fiscale S. Accelerated implementation of FQSqueezer novel genomic compression method. In: 2020 19th international symposium on parallel and distributed computing (ISPDC) IEEE; 2020. p. 158–163.
39. Xie S, He X, He S, Zhu Z. CURC: a CUDA-based reference-free read compressor. Bioinformatics 2022;38(12):3294–3296.
40. Danek A, et al. Indexes of Large Genome Collections on a PC. PLoS ONE 2014;9(10):1–12. https://dx.doi.org/10.1371/journal.pone.0109384.
41. Rahn R, et al. Journaled string tree—a scalable data structure for analyzing thousands of similar genomes on your laptop. Bioinformatics 2014;30(24):3499–3505.
42. Kowalski T, Grabowski S, Deorowicz S. Indexing arbitrary-length k-mers in sequencing reads. PloS one 2015;10(7):e0133198.
43. Bradley P, Den Bakker HC, Rocha EP, McVean G, Iqbal Z. Ultrafast search of all deposited bacterial and viral genomic data. Nature biotechnology 2019;37(2):152–159.
44. Belazzougui D, et al. Block Trees. Journal of Computer and System Sciences 2021;117:1–22.
45. Grabowski S, Bieniecki W. copMEM: finding maximal exact matches via sampling both genomes. Bioinformatics 2019;35(4):677–678. https://doi.org/10.1093/bioinformatics/bty670.
46. Grabowski S, Bieniecki W. copMEM2: robust and scalable maximum exact match finding. Bioinformatics 2023;39(5):btad313.
47. Liu Y, Zhang LY, Li J. Fast detection of maximal exact matches via fixed sampling of query K-mers and Bloom filtering of index K-mers. Bioinformatics 2019;35(22):4560–4567.
48. Břinda K, Lima L, Pignotti S, Quinones-Olvera N, Salikhov K, Chikhi R, et al. Efficient and robust search of microbial genomes via phylogenetic compression. Nature Methods 2025;22(4):692–697.
49. Hunt M, Lima L, Anderson D, Hawkey J, Shen W, Lees J, et al. AllTheBacteria-all bacterial genomes assembled, available and searchable. bioRxiv 2024;p. 2024–03.
50. Silva JM, Almeida JR. Enhancing metagenomic classification with compression-based features. Artificial Intelligence in Medicine 2024;156:102948.
51. Silva JM, Pratas D, Caetano T, Matos S. The complexity landscape of viral genomes. GigaScience 2022;11:giac079.
52. Gagie T, Puglisi S. Searching and Indexing Genomic Databases via Kernelization. Front Bioeng Biotechnol 2015;3. https://dx.doi.org/10.3389/fbioe.2015.00012.
53. Kuhnle A, et al. Efficient Construction of a Complete Index for Pan-Genomics Read Alignment. J Comput Biol 2020;27(4):500–513.

54. Sherman RM, Salzberg SL. Pan-genomics in the human genome era. Nature Reviews Genetics 2020;21:243–254.
55. Kuruppu S, et al. Relative Lempel-Ziv Compression of Genomes for Large-Scale Storage and Retrieval. In: Chávez E, Lonardi S, editors. String Processing and Information Retrieval - 17th International Symposium, SPIRE 2010, Los Cabos, Mexico, October 11-13, 2010. Proceedings, vol. 6393 of Lecture Notes in Computer Science Springer; 2010. p. 201–206.
56. Deorowicz S, Grabowski S. Robust relative compression of genomes with random access. Bioinformatics 2011;27(21):2979–2986.
57. Deorowicz S, et al. GDC 2: Compression of large collections of genomes. Sci Rep 2015;5:11565.
58. Wandelt S, Leser U. FRESCO: Referential compression of highly similar sequences. IEEE/ACM Transactions on Computational Biology and Bioinformatics 2013;10(5):1275–1288.
59. Grabowski S, Kowalski TM. MBGC: Multiple Bacteria Genome Compressor. GigaScience 2022 01;11:giab099.
60. Deorowicz S, et al. AGC: compact representation of assembled genomes with fast queries and updates. Bioinformatics 2023;39(3):btad097.
61. Storer JA, Szymanski TG. Data compression via textual substitution. Journal of the ACM (JACM) 1982;29(4):928–951.
62. Blackwell GA, Hunt M, Malone KM, Lima L, Horesh G, Alako BT, et al. Exploring bacterial diversity via a curated and searchable snapshot of archived DNA sequences. PLoS biology 2021;19(11):e3001421.
63. Kuruppu S, et al. Reference Sequence Construction for Relative Compression of Genomes. In: SPIRE, vol. 7024 of Lecture Notes in Computer Science Springer; 2011. p. 420–425.
64. Lan D, et al. Genozip: a universal extensible genomic data compressor. Bioinformatics 2021;37(16):2225–2230.

Table 3. Compression results – collections of pathogens.

		zstd -3 –long=31	Genozip best	NAF -3 –long=31	NAF -19 –long=31	AGC -a	AGC -a adjusted[1]	MBGC1 default	MBGC1 max	MBGC2 default	MBGC2 max
	ratio	12.4	44.3	137.1	176.6	27.2	228.4	416.2	(3)451.0	(2)458.6	(1)502.6
C. jejuni	ctime	(3)233.3	2152.7	440.1	2332.8	3117.5	804.8	(2)76.4	322.8	(1)64.5	259.0
55,627 genomes	dtime	116.5	1553.6	241.5	241.0	113.3	261.1	(3)49.0	70.0	(2)40.4	(1)39.9
98.38 GB	cmem	(2)2.35	105.52	(1)2.31	(3)2.66	5.09	6.21	8.50	6.18	7.51	6.36
	dmem	(2)2.15	63.86	2.70	(3)2.69	3.95	(1)0.78	5.65	5.06	4.20	4.93
S. enterica	ratio	27.5	65.9	1981.5	2175.6	100.0	3325.2	7516.8	(3)7671.2	(2)7793.1	(1)7994.1
cluster	ctime	84.9	346.2	200.3	271.0	458.0	168.6	(2)22.1	79.5	(1)19.7	(3)36.2
14,003 genomes	dtime	74.7	82.2	162.5	161.8	36.3	48.6	(2)19.1	19.3	(1)18.8	(2)19.1
67.12 GB	cmem	(2)2.36	99.15	(1)2.31	2.66	4.07	3.90	6.33	(3)2.46	5.57	(3)2.46
	dmem	2.15	62.92	2.27	2.27	0.99	(1)0.34	0.75	0.75	(2)0.59	(3)0.62
bacteria	ratio	15.8	48.5	369.0	434.0	36.1	628.2	1329.5	(3)1408.0	(2)1430.8	(1)1553.3
mixed	ctime	1009.2	28698.0	2101.6	6511.0	9540.0	3895.0	(2)280.3	1117.5	(1)228.5	(3)690.6
168,311 genomes	dtime	683.5	26590.0	1431.9	1428.5	545.1	1337.5	(3)274.7	307.1	(1)254.1	(2)262.9
587.26 GB	cmem	(2)2.36	130.06	(1)2.31	(3)2.66	6.70	9.15	21.05	13.07	19.45	12.79
	dmem	(2)2.15	65.07	(3)4.51	4.53	16.86	(1)1.61	9.11	9.50	8.09	8.82
	ratio	6.1	29.2	43.0	54.2	18.9	40.3	63.6	(3)73.0	(2)87.2	(1)97.3
C. jejuni	ctime	(3)5.0	61.5	8.7	121.6	52.5	17.7	(1)3.8	10.7	(2)4.7	9.8
1,024 genomes	dtime	(2)1.7	6.2	4.5	4.5	2.4	2.7	2.6	3.3	(1)1.6	(3)2.2
1.78 GB	cmem	1.96	12.23	1.91	2.25	3.04	2.65	1.63	(2)1.20	(3)1.30	(1)1.06
	dmem	1.79	5.99	1.76	1.76	(2)0.37	(1)0.31	1.26	1.18	(3)0.93	1.01
S. enterica	ratio	20.6	60.4	900.4	1024.3	62.3	1314.5	2174.6	(3)2197.3	(2)2228.6	(1)2252.3
cluster part	ctime	6.4	47.0	15.7	25.2	44.6	12.2	(1)2.5	6.0	(1)2.5	(3)2.8
1,024 genomes	dtime	3.1	4.7	11.8	11.8	2.7	3.4	(3)1.3	1.4	(1)1.0	(2)1.1
4.87 GB	cmem	2.32	30.71	2.30	2.64	4.03	3.34	2.06	(1)1.14	(3)1.33	(1)1.14
	dmem	2.15	16.22	2.16	2.16	(2)0.35	(1)0.28	0.62	0.62	(3)0.48	0.51
bacteria	ratio	14.1	47.6	177.1	214.6	11.2	172.0	301.0	(3)351.5	(2)379.4	(1)456.5
mixed	ctime	28.6	151.7	56.2	298.6	315.4	73.2	(1)11.6	33.1	(2)12.1	(3)23.6
4 × 1024 gen.	dtime	10.2	59.9	36.2	36.2	20.5	14.4	(3)7.3	8.2	(1)4.6	(2)5.7
14.94 GB	cmem	2.32	94.86	(3)2.31	2.66	4.44	4.77	3.86	(2)2.23	3.29	(1)2.19
	dmem	2.15	49.72	2.24	2.24	(2)1.77	(1)0.51	2.53	2.25	(3)1.89	2.06
	ratio	35.38	(1)114.98	59.58	(2)74.74	10.18	40.11	45.24	52.58	53.50	(3)60.68
Influenza	ctime	(3)6.39	224.29	(2)6.23	121.01	913.09	957.96	(1)5.78	12.60	6.46	11.75
817,587 seq.	dtime	(1)0.69	2.43	(3)1.11	(2)1.09	5.90	858.58	5.54	5.16	3.81	5.13
1.43 GB	cmem	(2)1.59	17.93	(1)1.46	(3)1.81	3.85	4.48	3.14	2.60	2.48	2.54
	dmem	(2)1.43	2.97	(2)1.43	(2)1.43	2.77	4.84	1.65	2.31	(1)1.08	2.21
	ratio	354.28	410.71	412.55	519.14	501.63	(1)914.22	510.85	529.40	(3)583.18	(2)617.17
COVID	ctime	42.35	143.18	58.46	127.59	1990.31	8434.00	(2)15.42	43.76	(1)12.83	(3)28.03
620,304 seq.	dtime	(1)7.92	16.55	(2)9.97	(3)9.98	15.59	186.57	13.76	25.24	13.20	15.61
18.83GB	cmem	(1)2.31	105.87	(1)2.31	(3)2.65	22.24	22.15	21.34	21.46	19.84	19.79
	dmem	(2)2.15	62.26	2.24	2.24	41.09	41.57	(3)2.18	28.60	(1)0.43	19.25

[1]AGC options adjusted for Influenza and COVID datasets are -s3000 -b10000, and -s1500 -b500 otherwise.

Table 4. Compression results – collections of *H. sapiens* genomes. NAF failed to compress the largest dataset within the 100,000 seconds limit (denoted with "—").

	HGSVCu (36 genomes, 102.88 GB)					HPRC (95 genomes, 290.13 GB)				
	ratio	ctime	dtime	cmem	dmem	ratio	ctime	dtime	cmem	dmem
zstd -3 –long=31	3.3	(1)180.7	154.9	(2)2.35	(1)2.15	3.4	(2)514.9	466.5	(2)2.35	(1)2.15
zstd -19 –long=31	4.2	9408.0	157.3	5.08	(1)2.15	4.4	25549.0	410.0	(3)5.10	(1)2.15
Genozip default	4.5	502.6	134.7	5.40	4.37	4.8	1355.5	548.9	5.54	3.62
Genozip -b best	4.7	645.5	175.8	101.31	67.33	4.9	1554.5	445.4	99.34	66.68
NAF -3 –long=31	4.2	740.0	337.2	(1)2.30	(1)2.15	4.1	2246.5	1018.8	(1)2.31	(1)2.15
NAF -19 –long=31	5.2	49906.0	333.2	(3)2.64	(1)2.15	—	—	—	—	—
AGC default	96.6	(2)194.0	(1)52.9	24.14	16.53	(2)201.3	(3)539.0	(1)157.3	27.69	23.33
MBGC1 max	(3)101.2	634.0	141.0	40.72	33.88	160.6	1611.3	398.6	42.49	40.58
MBGC2 default	(2)101.3	(3)212.6	(2)111.1	47.99	37.05	(3)181.3	(1)295.3	(2)274.1	54.57	43.36
MBGC2 max	(1)115.2	624.4	(3)128.1	38.55	36.93	(1)208.0	1517.5	(3)294.3	42.37	29.72

Table 5. Compression results – collections of yeast genomes.

	S. cerevisiae (39 genomes, totalling 493.98 MB)					S. paradoxus (36 genomes, totalling 436.43 MB)				
	ratio	ctime	dtime	cmem	dmem	ratio	ctime	dtime	cmem	dmem
GDC 2	(1)109.8	3.78	0.56	(1)0.52	(1)0.15	(2)80.7	20.52	0.83	(2)0.52	(1)0.18
zstd -3 –long=31	4.2	(1)0.90	0.56	(2)0.56	0.50	3.8	(1)0.71	(3)0.52	(1)0.50	0.44
zstd -19 –long=31	23.5	76.60	(2)0.38	1.55	0.50	17.3	77.98	(2)0.38	1.40	0.44
Genozip default	5.0	4.05	1.11	3.85	1.89	4.9	3.80	1.04	3.42	1.66
Genozip -b best	35.4	49.41	3.56	3.43	1.68	27.8	39.53	3.20	3.08	1.50
NAF -3 –long=31	67.0	2.61	1.04	0.64	(3)0.49	43.2	2.33	0.92	(3)0.58	(3)0.43
NAF -19 –long=31	77.0	26.94	1.03	0.97	(3)0.49	43.2	29.84	0.93	0.92	(3)0.43
AGC default	70.3	1.50	(1)0.32	(3)0.59	(2)0.33	43.4	(3)1.63	(1)0.28	0.80	(2)0.32
MBGC1 default	86.6	(3)1.21	0.75	1.94	0.81	43.9	1.64	1.06	1.94	0.75
MBGC1 max	91.0	3.07	0.92	1.49	0.88	61.4	3.00	1.09	1.42	0.76
MBGC2 default	(3)101.9	(2)0.97	(3)0.48	1.83	0.53	(3)77.5	(2)1.37	0.53	1.76	0.49
MBGC2 max	(2)105.5	2.64	0.78	1.40	0.78	(1)83.6	2.70	0.83	1.42	0.77

Table 6. Compression results – collections of RNA.

	SILVA 132 LSURef (610.3 MB)					SILVA 132 SSURef (3.28 GB)				
	ratio	ctime	dtime	cmem	dmem	ratio	ctime	dtime	cmem	dmem
zstd -3 –long=31	17.85	(1)2.51	(2)0.37	(1)0.70	0.62	15.51	(1)14.85	(3)2.09	(2)2.34	(2)2.15
zstd -19 –long=31	37.20	28.68	(1)0.34	2.05	0.62	32.44	167.61	(1)1.72	4.75	(2)2.15
Genozip default	37.31	18.91	1.07	9.49	1.43	33.97	111.00	(2)1.97	14.21	2.67
Genozip -b best	(1)51.98	207.56	1.93	8.09	1.34	(1)42.74	316.30	3.59	39.36	6.72
NAF -3 –long=31	31.90	(2)2.68	0.52	(2)0.74	(2)0.61	25.92	(3)16.69	2.87	(1)2.31	2.45
NAF -19 –long=31	41.49	43.78	(3)0.50	(3)1.08	(2)0.61	34.97	421.16	2.72	(3)2.66	2.45
AGC -a	12.71	40.42	2.20	1.66	1.47	11.56	7839.00	16.34	8.92	8.59
AGC -a -s3000 -b10000	28.39	60.19	154.97	1.95	1.73	22.81	7820.00	1957.78	9.70	7.88
MBGC1 default	37.91	(3)2.90	2.24	1.37	0.67	31.45	(2)15.73	14.34	5.51	2.49
MBGC1 max	(3)45.54	5.43	2.24	1.23	0.99	34.06	44.17	14.60	5.41	4.93
MBGC2 default	44.47	2.92	1.59	1.21	(1)0.50	(3)36.94	20.85	10.09	5.41	(1)2.00
MBGC2 max	(2)50.82	5.31	2.32	1.22	0.95	(2)37.62	43.53	14.36	5.42	4.91

Click here to access/download
Supplementary Material
MBGC2_supplementary.pdf
