# Supplementary material for: MBGC2: Boosting compression via efficient encoding of approximate matches in genome collections
Source: Gigascience. 2026 Jan 21;15:giag008. doi: 10.1093/gigascience/giag008 (PMC12934354; doi:10.1093/gigascience/giag008)
Supplement: giag008_Authors_Response_To_Reviewer_Comments_original_submission [file giag008_authors_response_to_reviewer_comments_original_submission.pdf]

Author's Response To Reviewer Comments

We are grateful for reported issues and suggestions concerning the usability of the MBGC tool.
Together with some other improvements, we decided to address them where it was possible, and have released a new version 2.1. The changes in the new version include:
* lossy compression and overriding existing files now require explicit request
* addressed a compression ratio regression for proteins introduced in version 2.0
* improved protein archiving increases decompression speed ~2.5 times
* improved compression of small collections in a single multi-FASTA file
* lossless handling of malformed headers
* decompression of gz archives with multiple concatenated streams
* reduced memory usage in decompressing "max" mode single-file archives

Results of experiments concerning MBGC2 are updated to the newest version (v2.1) and replaced in Tables 3-6 in the main paper and in Tables 1-3, and 5-9 in supplementary material. Unfortunately, results in Table 4 of the supplementary material remain unchanged (version MBGC 2.0), since the test platform was not available.
Results in Table 2 of the main paper and Table 10 of the supplementary materials remain the same (no changes to compression ratio for the selected datasets).

All the changes in the text are marked red in the manuscript.

Reviewer #1: The authors developed a software for compression of genomic data in the FASTA format. The uses of FASTA are very broad, from files containing a small number of long chromosomes of animals and plants, to files containing millions of short sequences representing variations of a bacterial or viral genome. FASTA also supports both nucleotide and amino acid sequences. The authors' software attempts to advance the compression field, specifically focused on FASTA files containing a large number of reasonably similar nucleotide sequences, where the improvement compared to existing method is essentially in better recognizing similarities between sequences and better encoding similarities and differences.

I commend the authors for tackling this specific problem which has not received as much attention in the genomic compression field compared to FASTQ and BAM compression.

I cloned the software from github, compiled it, and ran it on my Windows laptop, under WSL2. I tested a number of different FASTA files - including a file of PacBio coronavirus consensus sequences, a full human genome (GRCh38), a FASTA file I downloaded from NCBI ages ago and I already forgot what the data is, but it contains short 51bp sequences, and another coronavirus file, this time with official sequences. I compressed each with mbgc and genozip for comparison. The purpose was not run a formal benchmark, neither was it to test on a representative variety of data, rather the goal was to just to get an informal general sense of the areas were the software performs well and its limitations.

- On the human genome data, mgbc was very significantly slower, but compressed significantly better than Genozip (464MB vs 637MB). However, it crashed when trying to compress the .gz file and agreed to work only after manually gunzipping it.

RESPONSE:
Fixed in v2.1. Thank you.

- On the official coronavirus sequences it performed much worse than Genozip (6 MB fasta -> 485K for mbgc vs 76K for genozip)

RESPONSE:

Indeed, MBGC works better with large collections of files, rather than individual and small files.
In v2.1 we slightly modified single-file processing (initial reference selection) and decided to apply a sequential matching strategy in cases where the file is too small to gain speed from multithreading (i.e., below ~8MB). Changes improved the compression for the above file to 19KB.

- On the NCBI data mbgc segfaulted

RESPONSE:
This NCBI dataset contains lines starting with the ';' symbol, which are currently not supported by MBGC. Now, MBGC in v2.1 informs a user about an error with parsing the file.

- On the pacbio viral consensus data it worked well and compressed approximately 30% better than Genozip.

The manuscript itself is generally well written. I have three comments:
1. Before publishing, I recommend investing in extensive quality assurance - download a wide range of fasta files from many different sources - the FASTA format is less well-defined that it might seem and many files out there have all kinds of quirks. There are too many bugs at the moment, which need to be ironed out.

RESPONSE:
Thank you for the suggestion. Indeed, QA is an important and laborious process. Ensuring complete reliability in handling all available FASTA files is a very difficult, if not impossible, task. Nevertheless, we constantly strive to achieve this. To this end, in the recent release of MBGC (v2.1), we already resolved some of the issues we detected (e.g., excessive memory usage in appending many large genomes to an existing archive) and some of those you pointed out.

2. The compression is not lossless - the recovered file after decompression is of different size than the original file. This is big drawback, as users routinely use md5sum to verify that the data is recovered correctly after decompression. Ideally, this should be fixed, but if its by design and cannot be fixed, the manuscript needs to explain this.

RESPONSE:
Indeed, although in all main and supplementary experiments, the compression was lossless, MBGC v2.0 implicitly applied lossy compression to some older or non-regular FASTA (e.g., with varying line length in sequences or with windows EOL symbols convention).
To fix this, in the new release (v2.1), we decided to allow lossy mode only with explicit user permission (-L option). We also managed to enable lossless compression in some cases, e.g., for irregular headers (although we still only accept those that start with the symbol '>').
The README.md file and version v2.1 release information provide clarifications regarding MBGC limitations in supporting lossless compression of FASTA files.
Please note that in Table 1 notes, we already pointed out some limitations: "Some specialized genome compressors correctly preserve line length format in sequences only if it is consistent throughout a whole file (e.g., in case of NAF, GDC~2, MBGC2) or an individual sequence (e.g., HRCM)."
In the last paragraph of the introduction, we added the sentence: "MBGC2 compresses the vast majority of FASTA files losslessly, and in the case of obsolete or irregular files, it allows for lossy compression."

3. There are clearly some types of data on which this software does very well and represents an improvement vs existing options, but there are also types of data in which it doesn't do as well as existing software. It would be good if the authors are able to generalize from their tests (beyond the few files in the benchmark) and inform readers regarding the types of data they can expect this software to compress excellently.

RESPONSE:
Now the first paragraph of the Conclusion section has been augmented to clarify when a user can expect better, slightly worse, or unsatisfactory performance from MBGC2.

Thank you for all your comments, and we are grateful for pointing out the bugs in our tool.

Reviewer #2: The manuscript presents MBGC2, an enhanced version of the MBGC genome compressor. MBGC2 introduces approximate-match encoding, faster multi-threaded decompression, and new usability improvements. Benchmarking demonstrates clear gains in compression ratio and decompression speed compared to MBGC1.

Major comments:

1) The context regarding recent genome compressors, particularly GDC2 and Genozip, needs improvement. The authors should explicitly state how MBGC2 differs from these tools, clearly positioning MBGC2's novel encoding approach, especially concerning approximate matches.

RESPONSE:
GDC2 first uses a hashtable to search for the exact matches between sequences and the reference. To avoid some HT lookups and speed up matching, it checks if the bases surrounding a match are possibly SNPs or short indels. During the second-level factoring, some of the mismatched literals with their surrounding are matched to sequences encoded during the first stage of the processing. This achieves an effect similar to both versions of MBGC, where matches do not only apply to the reference, but also occur between all compressed sequences.
Next, the remaining literals, containing probable mismatches or indels, are contextually encoded by GDC2, where the context is the recently encoded symbol. No technique even remotely similar to MBGC2 adjacent encoding using the exclusive encoding mismatch matrix is used.
The more recent tool, AGC, uses a novel approach based on so-called segment splitters and does not take advantage of mismatches, both in matching segments and in encoding them.
To highlight the above differences, we added the following to the Conclusions section:
"Compared to recent FASTA file compression tools such as GDC~2, HRCM, NAF, or AGC that encode mismatches as literals, MBGC2 employs the exclusive mismatch encoding matrix that maps mismatches relative to the reference into 2-bit codes, prioritizing evolutionary transitions (G$\leftrightarrow$A, C$\leftrightarrow$T)."
We also added two sentences to the description of the Gaps delta encoding technique to provide context and clearly show the difference between GDC2 and MBGC2 in match position encoding. Namely:
"Due to the similarity of genomic sequences, neighbouring exact matches are likely to be mapped to corresponding locations in the reference. Specialized compressors exploit this to reduce the storage cost of their positions by employing delta encoding (e.g., GDC~2~\citep{DDN2015} and HRCM~\citep{YJLLHW2019}."
Regarding the Genozip, please note the sentence in the Results section: "It targets various formats, however the authors do not describe the details of reference-free FASTA file compression method". Moreover, although the source code of Genozip is available on Github, the authors applied a license that prohibits reverse engineering.

2) Benchmark comparisons use varying computational resources, particularly thread and memory usage (e.g., MBGC1 using 8 threads, NAF being single-threaded, others using up to 28 threads). Provide at least one benchmark scenario where all tools use identical computational resources.

RESPONSE:
We would like to emphasize that in the main experiments, none of the tools were limited in any way. NAF does not support multithreading, and the limit on MBGC1 results from its implementation (default settings). We have also supplemented the missing information about the default AGC multithreading

setting (resulting from the number of processor cores of our workstation). In the revised Results section, we now have:
"MBGC1 uses 8 threads, AGC 14 threads, NAF is single-threaded, and the rest of the tools use 28 threads. MBGC1 and AGC limit multithreading by their design."
Nevertheless, we added experiments to the supplement in which the number of threads is limited to 1 and 6, and an experiment in which 7z and BSC can use all processor threads (Tables 11-13). In all cited studies, we did not encounter experiments on compressors in which available memory was actively limited. Unfortunately, most of the tested tools do not allow for setting an explicit memory bound. Finally, we decided to report only the peak usage, allowing readers to assess resource requirements. We hope you find this sufficient.

Clearly justify your choices regarding datasets and parameters.

RESPONSE
At the beginning of the Results section, we presented the basic premise behind the selection of datasets: "The test collection consists of several real genome datasets, used in prior works on FASTA collection compression". Most of the datasets come from the cited Sequence Compression Benchmark (Kryukov K, 2020) and the paper on AGC (Deorowicz S, 2023). In our opinion, this is a sufficiently diverse selection for the purposes of evaluating FASTA collection compressors.
We expanded the discussion of selected tools and their settings in the 2nd paragraph of Results section. We hope that the motivations for choosing the parameters for the tools are now clear.

3) While MBGC2 claims broad applicability, benchmarks clearly show performance limitations with protein datasets. This limitation should be explicitly qualified throughout the manuscript, particularly in the abstract and conclusions.

RESPONSE:
Now the first paragraph of the Conclusion section has been augmented to clarify when a user can expect better, slightly worse, or unsatisfactory performance from MBGC2.
In the abstract, we added the sentence:
"The tool supports working with single genomes or amino acid collections, but does not guarantee such high performance in these cases".
Please note that in the newest release (v2.1) we managed to significantly improve the performance on protein collections (increased decompression speed ~2.5 times together with improved compression ratio).

Minor comments:

- Key parameters for approximate match encoding (mismatch matrix, scoring strategy) should be clearly defined earlier or highlighted prominently in the main manuscript.

RESPONSE:
In the last paragraph of the introduction, we added the sentence:
"The techniques we have introduced include dynamic determination of approximate match boundaries using a mismatch scoring routine and a simple yet effective static exclusive mismatch encoding matrix."
The extended conclusion also mentions the exclusive mismatch encoding matrix in the context of competitors approach to encode mismatches.

- Clarify benchmark metrics: explicitly indicate if reported memory usage is peak or average and consider reporting CPU-seconds or utilization.

RESPONSE:
We extended a sentence in the 1st paragraph of the Results section:
"Peak memory usage, i.e., maximum resident set size of the process during its lifetime}

, ``cmem'' (resp. ``dmem'') for compression (resp. decompression) and dataset sizes are specified in GB, where $\textrm{G} = 10^{9}$."
We reported CPU utilization in the new experiments concerning imposing limits on threads usage (Tables 11-13 of the supplementary). This should allow for a better assessment of tools' scalability (in terms of speed and memory utilization).

- Correct typographical inconsistencies in species names (e.g., consistently use "H. sapiens" instead of "H. Sapiens").

RESPONSE:
Fixed.

The manuscript already provides significant improvements over MBGC, showing strong engineering and comprehensive benchmarking. However, addressing the stated issues regarding mentioned will strength the manuscript. I recommend a Major Revision.

Thank you for all your comments

Reviewer #3: The paper presents an enhanced version of Multiple Bacteria Genome Compressor (MBGC), i.e., MBGC 2, for compressing collections of FASTA files. Compared to the previous version, MBGC2 improves both the compression ratio and efficiency. Its effectiveness was demonstrated through the comparison experiments with other FASTA collection compressor like Genozip, NAR, AGC, zstd, and the original MBGC1. The manuscript is well organized and clearly written, yet I have some concerns on the experiment studies as follows:

1. As reviewed in the first section, there are many FASTA compressor. The reason of selecting the current compared methods in the experiments should be explained.

RESPONSE:
We expanded the discussion of selected tools in the 2nd paragraph of Results section and explained issues related to other FASTA compressors we tested in the beginning of the 4th section of supplementary materials. We hope that the motivations for the selection of competitors are now clear.

2. The most recently published phylogenetic compression method in [48] is closely related to this work. It would be interesting to see comparison between MBGC2 and method proposed in [48].

RESPONSE:
Thank you for the suggestion. The work [48] already presented experiments involving MBGC2. We added the subsection 4.1 in the supplementary, where we summarize their findings in the area of compression and explore further how the presented approach boosts ratio of MBGC2 in different compression modes.
We also added a sentence in the Result section of the main paper: "Furthermore, supplementary material (Subsection 4.1) presents results concerning boosting compression through leveraging phylogenetic relationships [48]."

3. As stated in Page 5, the compared methods used different number of threads. It might be unfair to compared them in terms of time/space efficiency. Would it be possible to compared the methods with the same number of threads or computation resources?

RESPONSE:
We would like to emphasize that in the main experiments, none of the tools were limited in any way. NAF

does not support multithreading, and the limit on MBGC1 results from its implementation (default settings). We have also supplemented the missing information about the default AGC multithreading setting (resulting from the number of processor cores of our workstation). In the revised Results section, we now have: "MBGC1 uses 8 threads, AGC 14 threads, NAF is single-threaded, and the rest of the tools use 28 threads.
MBGC1 and AGC limit multithreading by their design."
Nevertheless, we decided to add experiments to the supplement in which the number of threads is limited to 1 and 6, and an experiment in which 7z and BSC can use all processor threads (Tables 11-13). Their results additionally report CPU utilization, which should allow for a better assessment of tools' scalability (in terms of speed and memory utilization).

Thank you for all your comments.

Editorial Manager®

Cookie Preference Center

We use cookies which are necessary to make our site work. We may also use additional cookies to analyze, improve and personalize our content and your digital experience. For more information, see our Cookie Policy.

You may choose not to allow some types of cookies. However, blocking some types may impact your experience of our site and the services we are able to offer. See the different category headings below to find out more or change your settings.

Allow all

Manage Consent Preferences

Strictly Necessary Cookies

Always active

These cookies are necessary for the website to function and cannot be switched off in our systems. They are usually only set in response to actions made by you which amount to a request for services, such as setting your privacy preferences, logging in or filling in forms. You can set your browser to block or alert you about these cookies, but some parts of the site will not then work.

Functional Cookies

These cookies enable the website to provide enhanced functionality and personalisation. They may be set by us or by third party providers whose services we have added to our pages. If you do not allow these cookies then some or all of these services may not function properly.

Performance Cookies

These cookies allow us to count visits and traffic sources so we can measure and improve the performance of our site. They help us to know which pages are the most and least popular and see how visitors move around the site.

Targeting Cookies

These cookies may be set through our site by our advertising partners. They may be used by those companies to build a profile of your interests and show you relevant adverts on other sites. If you do not allow these cookies, you will experience less targeted advertising.

Cookie List

Clear

Apply Cancel
Consent Leg.Interest

Confirm my choices

Powered by onetrust
