# Supplementary material for: MBGC2: Boosting compression via efficient encoding of approximate matches in genome collections
Source: Gigascience. 2026 Jan 21;15:giag008. doi: 10.1093/gigascience/giag008 (PMC12934354; doi:10.1093/gigascience/giag008)
Supplement: giag008_Authors_Response_To_Reviewer_Comments_Revision_1 [file giag008_authors_response_to_reviewer_comments_revision_1.pdf]

Author's Response To Reviewer Comments

Below are the responses to reviewer comments.

Reviewer #2: The revision addresses almost all of the earlier concerns. Only three small issues remain that should be corrected for clarity and internal consistency.

1) The final line still states that MBGC2 is suitable for "genomic data of any size and type," which contradicts the limitations you clearly outline earlier in the same paragraph, namely reduced performance on single-file inputs, very small collections, and proteins. Please align the closing sentence with those stated limitations.

RESPONSE:
Thank you for this remark. We modified the closing sentence:
"All of these enhancements make MBGC2 a highly efficient, reliable, and easy-to-use tool for the management, storage, and transfer large collections of genomes stored in multiple FASTA files."
to clearly exclude single-file inputs, very small collections, and proteins.

2) Because MBGC2 can operate in either mode, the manuscript should contain one explicit sentence in the Methods or Results clarifying that all benchmarks were run in lossless mode.

RESPONSE:
We added the sentence in the second paragraph of the Results section:
"Except for AGC, and MBGC1, which do not preserve the sequences line length, all tools performed lossless compression in all presented experiments."

3) The main text briefly notes that supplementary material contains imposed-thread-limit experiments, but it doesn't point readers to the specific tables. A short reference to Supplementary Tables would make these results easy to find.

RESPONSE:
We added the references:
"The supplementary material provides more results (including Table~8 with protein datasets and Tables~11~and~12 presenting experiments with imposed limits on the number of threads), details on datasets, and the test methodology."

Once these are addressed, the manuscript should be ready for acceptance.

Reviewer #3: My previous concerns have been well addressed.

We decided for a minor modification in the supplementary material to acknowledge the tool that implements phylogenetic compression pipeline:
"In ~\citep{Brinda2025} phylogenetic compression, implemented in the MiniPhy tool, was applied to the 661k collection using either xz or MBGC as a low-level compressor."
We are grateful for all your comments.

Cookie Preference Center

We use cookies which are necessary to make our site work. We may also use additional cookies to analyze, improve and personalize our content and your digital experience. For more information, see our Cookie Policy.

You may choose not to allow some types of cookies. However, blocking some types may impact your experience of our site and the services we are able to offer. See the different category headings below to find out more or change your settings.

Allow all

Manage Consent Preferences

Strictly Necessary Cookies

Always active

These cookies are necessary for the website to function and cannot be switched off in our systems. They are usually only set in response to actions made by you which amount to a request for services, such as setting your privacy preferences, logging in or filling in forms. You can set your browser to block or alert you about these cookies, but some parts of the site will not then work.

Functional Cookies

These cookies enable the website to provide enhanced functionality and personalisation. They may be set by us or by third party providers whose services we have added to our pages. If you do not allow these cookies then some or all of these services may not function properly.

Performance Cookies

These cookies allow us to count visits and traffic sources so we can measure and improve the performance of our site. They help us to know which pages are the most and least popular and see how visitors move around the site.

Targeting Cookies

These cookies may be set through our site by our advertising partners. They may be used by those companies to build a profile of your interests and show you relevant adverts on other sites. If you do not allow these cookies, you will experience less targeted advertising.

Cookie List

Clear

Apply Cancel
Consent Leg.Interest

Confirm my choices

Powered by onetrust
