# Supplementary material for: MBGC2: Boosting compression via efficient encoding of approximate matches in genome collections
Source: Gigascience. 2026 Jan 21;15:giag008. doi: 10.1093/gigascience/giag008 (PMC12934354; doi:10.1093/gigascience/giag008)
Supplement: giag008_Reviewer_1_Report_original_submission — Divon Lan -- 9/7/2025 [file giag008_reviewer_1_report_original_submission.pdf]

Reviewer Report

Title: MBGC2: Boosting compression via efficient encoding of approximate matches in genome collections

Version: Original Submission **Date:** 9/7/2025

Reviewer name: Divon Lan

Reviewer Comments to Author:

The authors developed a software for compression of genomic data in the FASTA format. The uses of FASTA are very broad, from files containing a small number of long chromosomes of animals and plants, to files containing millions of short sequences representing variations of a bacterial or viral genome. FASTA also supports both nucleotide and amino acid sequences. The authors' software attempts to advance the compression field, specifically focused on FASTA files containing a large number of reasonably similar nucleotide sequences, where the improvement compared to existing method is essentially in better recognizing similarities between sequences and better encoding similarities and differences.I commend the authors for tackling this specific problem which has not received as much attention in the genomic compression field compared to FASTQ and BAM compression.I cloned the software from github, compiled it, and ran it on my Windows laptop, under WSL2. I tested a number of different FASTA files - including a file of PacBio coronavirus consensus sequences, a full human genome (GRCh38), a FASTA file I downloaded from NCBI ages ago and I already forgot what the data is, but it contains short 51bp sequences, and another coronavirus file, this time with official sequences. I compressed each with mbgc and genozip for comparison. The purpose was not run a formal benchmark, neither was it to test on a representative variety of data, rather the goal was to just to get an informal general sense of the areas were the software performs well and its limitations.- On the human genome data, mgbc was very significantly slower, but compressed significantly better than Genozip (464MB vs 637MB). However, it crashed when trying to compress the .gz file and agreed to work only after manually gunzipping it.- On the official coronavirus sequences it performed much worse than Genozip (6 MB fasta -> 485K for mbgc vs 76K for genozip)- On the NCBI data mbgc segfaulted- On the pacbio viral consensus data it worked well and compressed approximately 30% better than Genozip.The manuscript itself is generally well written. I have three comments:1. Before publishing, I recommend investing in extensive quality assurance - download a wide range of fasta files from many different sources - the FASTA format is less well-defined that it might seem and many files out there have all kinds of quirks. There are too many bugs at the moment, which need to be ironed out.2. The compression is not lossless - the recovered file after decompression is of different size than the original file. This is big drawback, as users routinely use md5sum to verify that the data is recovered correctly after decompression. Ideally, this should be fixed, but if its by design and cannot be fixed, the manuscript needs to explain this.3. There are clearly some types of data on which this software does very well and represents an improvement vs existing options, but there are also types of data in which it doesn't do as well as existing software. It would be good if the authors are able to generalize from their tests (beyond the few

files in the benchmark) and inform readers regarding the types of data they can expect this software to compress excellently.

Level of Interest

Please indicate how interesting you found the manuscript: Choose an item.

Quality of Written English

Please indicate the quality of language in the manuscript: Choose an item.

Declaration of Competing Interests

Please complete a declaration of competing interests, considering the following questions:

- Have you in the past five years received reimbursements, fees, funding, or salary from an organisation that may in any way gain or lose financially from the publication of this manuscript, either now or in the future?
- Do you hold any stocks or shares in an organisation that may in any way gain or lose financially from the publication of this manuscript, either now or in the future?
- Do you hold or are you currently applying for any patents relating to the content of the manuscript?
- Have you received reimbursements, fees, funding, or salary from an organization that holds or has applied for patents relating to the content of the manuscript?
- Do you have any other financial competing interests?
- Do you have any non-financial competing interests in relation to this paper?

If you can answer no to all of the above, write 'I declare that I have no competing interests' below. If your reply is yes to any, please give details below.

I do not have competing interests according to your defintion. However, for full disclosure, I will mention that I am the author of Genozip, a commerical universal genomic compression software. However, FASTA compression is not our area of focus either technically or commercially.

I agree to the open peer review policy of the journal. I understand that my name will be included on my report to the authors and, if the manuscript is accepted for publication, my named report including any attachments I upload will be posted on the website along with the authors' responses. I agree for my report to be made available under an Open Access Creative Commons CC-BY license (http://creativecommons.org/licenses/by/4.0/). I understand that any comments which I do not wish to be included in my named report can be included as confidential comments to the editors, which will not be published.

Choose an item.

To further support our reviewers, we have joined with Publons, where you can gain additional credit to further highlight your hard work (see: https://publons.com/journal/530/gigascience). On publication of this paper, your review will be automatically added to Publons, you can then choose whether or not to claim your Publons credit. I understand this statement.

Yes Choose an item.
