# Supplementary material for: MBGC2: Boosting compression via efficient encoding of approximate matches in genome collections
Source: Gigascience. 2026 Jan 21;15:giag008. doi: 10.1093/gigascience/giag008 (PMC12934354; doi:10.1093/gigascience/giag008)
Supplement: giag008_Reviewer_2_Report_original_submission — Jorge Miguel Ferreira da Silva -- 9/10/2025 [file giag008_reviewer_2_report_original_submission.pdf]

Reviewer Report

Title: MBGC2: Boosting compression via efficient encoding of approximate matches in genome collections

Version: Original Submission **Date:** 9/10/2025

Reviewer name: Jorge Miguel Ferreira da Silva

Reviewer Comments to Author:

The manuscript presents MBGC2, an enhanced version of the MBGC genome compressor. MBGC2 introduces approximate-match encoding, faster multi-threaded decompression, and new usability improvements. Benchmarking demonstrates clear gains in compression ratio and decompression speed compared to MBGC1.Major comments:1) The context regarding recent genome compressors, particularly GDC2 and Genozip, needs improvement. The authors should explicitly state how MBGC2 differs from these tools, clearly positioning MBGC2's novel encoding approach, especially concerning approximate matches.2) Benchmark comparisons use varying computational resources, particularly thread and memory usage (e.g., MBGC1 using 8 threads, NAF being single-threaded, others using up to 28 threads). Provide at least one benchmark scenario where all tools use identical computational resources. Clearly justify your choices regarding datasets and parameters.3) While MBGC2 claims broad applicability, benchmarks clearly show performance limitations with protein datasets. This limitation should be explicitly qualified throughout the manuscript, particularly in the abstract and conclusions.Minor comments:- Key parameters for approximate match encoding (mismatch matrix, scoring strategy) should be clearly defined earlier or highlighted prominently in the main manuscript.- Clarify benchmark metrics: explicitly indicate if reported memory usage is peak or average and consider reporting CPU-seconds or utilization.- Correct typographical inconsistencies in species names (e.g., consistently use "H. sapiens" instead of "H. Sapiens").The manuscript already provides significant improvements over MBGC, showing strong engineering and comprehensive benchmarking. However, addressing the stated issues regarding mentioned will strength the manuscript. I recommend a Major Revision.

Level of Interest

Please indicate how interesting you found the manuscript: Choose an item.

Quality of Written English

Please indicate the quality of language in the manuscript: Choose an item.

Declaration of Competing Interests

Please complete a declaration of competing interests, considering the following questions:

- Have you in the past five years received reimbursements, fees, funding, or salary from an organisation that may in any way gain or lose financially from the publication of this manuscript, either now or in the future?
- Do you hold any stocks or shares in an organisation that may in any way gain or lose financially from the publication of this manuscript, either now or in the future?
- Do you hold or are you currently applying for any patents relating to the content of the manuscript?
- Have you received reimbursements, fees, funding, or salary from an organization that holds or has applied for patents relating to the content of the manuscript?
- Do you have any other financial competing interests?
- Do you have any non-financial competing interests in relation to this paper?

If you can answer no to all of the above, write 'I declare that I have no competing interests' below. If your reply is yes to any, please give details below.

I declare that I have no competing interests.

I agree to the open peer review policy of the journal. I understand that my name will be included on my report to the authors and, if the manuscript is accepted for publication, my named report including any attachments I upload will be posted on the website along with the authors' responses. I agree for my report to be made available under an Open Access Creative Commons CC-BY license (http://creativecommons.org/licenses/by/4.0/). I understand that any comments which I do not wish to be included in my named report can be included as confidential comments to the editors, which will not be published.

Choose an item.

To further support our reviewers, we have joined with Publons, where you can gain additional credit to further highlight your hard work (see: https://publons.com/journal/530/gigascience). On publication of this paper, your review will be automatically added to Publons, you can then choose whether or not to claim your Publons credit. I understand this statement.

Yes Choose an item.
